# HGA: *denovo* genome assembly method for bacterial genomes using high coverage short sequencing reads

**DOI:** 10.1186/s12864-016-2515-7

**Published:** 2016-03-05

**Authors:** Anas A. Al-okaily

**Affiliations:** Computer Science & Engineering Department, University of Connecticut, Storrs, 06269 CT USA

**Keywords:** Computational genomic, De novo genome assembly, Contigs assembly

## Abstract

**Background:**

Current high-throughput sequencing technologies generate large numbers of relatively short and error-prone reads, making the *de novo* assembly problem challenging. Although high quality assemblies can be obtained by assembling multiple paired-end libraries with both short and long insert sizes, the latter are costly to generate. Recently, GAGE-B study showed that a remarkably good assembly quality can be obtained for bacterial genomes by state-of-the-art assemblers run on a single short-insert library with very high coverage.

**Results:**

In this paper, we introduce a novel *hierarchical genome assembly* (HGA) methodology that takes further advantage of such very high coverage by independently assembling disjoint subsets of reads, combining assemblies of the subsets, and finally re-assembling the combined contigs along with the original reads.

**Conclusions:**

We empirically evaluated this methodology for 8 leading assemblers using 7 GAGE-B bacterial datasets consisting of 100 bp Illumina HiSeq and 250 bp Illumina MiSeq reads, with coverage ranging from 100x– ∼200x. The results show that for all evaluated datasets and using most evaluated assemblers (that were used to assemble the disjoint subsets), HGA leads to a significant improvement in the quality of the assembly based on N50 and corrected N50 metrics.

**Electronic supplementary material:**

The online version of this article (doi:10.1186/s12864-016-2515-7) contains supplementary material, which is available to authorized users.

## Background

*De novo* genome assembly is one of the fundamental problems in bioinformatics. Interest in the problem has been renewed in the past decade due to the advent of *next-generation sequencing* (NGS) technologies, which generate large numbers of short (100–400 bp) reads with relative low sequencing error rates. There are three main approaches for *de novo* genome assembly, the greedy strategy, the string overlap graph, and the de Bruijn graph. In the greedy approach, the assembly algorithm works by selecting seed reads and greedily extending them with the maximum overlapping reads until no more overlap is possible. This approach was adopted by some early assemblers such as SSAKE [1], SHARCGS [2], and VCAKE [3]. Unfortunately the greedy approach doesn’t take into account ambiguities induced by repeats and sequencing errors, resulting in a large number of mis-assembly errors.

The string overlap graph approach is based on building a graph with reads as nodes and edges connecting every pair of nodes so that the corresponding reads overlap given a minimum overlap length. Building the overlap graph involves a compute-intensive all-against-all pairwise comparison step. After constructing the graph, the reads layout is computed and the consensus sequence is determined using multiple sequence alignment. This approach was implemented in assemblers such as Newbler [4], SGA [5], and CABOG [6] is more efficient for long reads such as those generated by Sanger and 454 sequencing.

The third approach, based on the de Bruijn graph model [7], is by far the most commonly used in assemblers targeted at NGS data, including ABySS [8], ALLPATHS-LG [9], Euler-USR [10], MaSuRCA [11], SoapDenovo2 [12], SPAdes [13] and Velvet [14]. Building the de Bruijn graph starts by collecting all substrings of length *k* (referred to as *k*-mers) of all reads; then building a graph with *k*-mers as nodes and edges connecting two *k*-mers *a* and *b* if the suffix of length (*k*−1) of *a* matches the prefix of length *k*−1 of *b* and the *k*+1-mer obtained by overlapping *a* and *b* appears in the reads. The de Bruijn graph can be built in linear time but storing it requires very large amounts of memory, typically much larger than the string overlap graph. After building the de Bruijn graph, each assembler uses several heuristics to simplify graph structures such as cycles and bulges which mainly induced by repeats in the genome, and bubbles and tips which mainly induced by sequencing errors and heterozygous sites. Lastly, assemblers select a set of simple paths in the de Bruijn graph that would eventually form the contigs. For further details on algorithms for NGS genome assembly, the reader is directed to [15–17].

Despite the large number of assemblers that were developed, genome assembly from NGS reads remains challenging. In particular, recent benchmarking efforts [18, 19] have shown that the performance of existing assemblers is highly variable from dataset to dataset and significantly degrades with the complexity of the genome. For large genomes, the highest quality assemblies are currently obtained by jointly assembling multiple paired-end libraries generated with a wide range of insert sizes using algorithms such as ALLPATHS-LG [9]. However, sequencing libraries with long insert sizes are costly to generate. Recently, the GAGE-B study [20] showed that for bacterial genomes a comparable assembly quality can be obtained by running state-of-the-art assemblers MaSuRCA [11] and SPAdes [13] on a single short-insert library with very high coverage (100–300 ×). In contrast, other assemblers including ABySS [8], CABOG [6], SoapDenovo2 [12], SGA [5], and Velvet [14] appeared less able to take advantage of such high sequencing depth, with nearly flat N50 contig length above 100 × (see Figure 1 in [20]).

In this paper, we introduce a novel *hierarchical genome assembly* (HGA) methodology designed to take advantage of such a high coverage by independently assembling disjoint subsets of reads, combining the assemblies of the subsets, and finally re-assembling the combined contigs along with the original reads. Empirical evaluation of this methodology for 8 leading assemblers using 7 GAGE-B bacterial datasets consisting of 100 bp Illumina HiSeq and 250 bp Illumina MiSeq reads shows that HGA leads to a significant improvement in assembly quality, based on N50 and corrected N50 metrics, for *all* evaluated datasets using *most* evaluated assemblers (that were used to assemble the disjoint subsets).

Our contribution in this paper is to resolve the assembly problem using different approaches. It’s summarized in the following observations. Firstly, the complexity of the graph, in terms of tips, bubbles, bulges, cycles, and false branching; *will be less* using lower converges and short kmer than higher coverage and short kmer. In addition, *resolving these complexities will be more efficient and the resulting contigs will have less errors*, despite the fact that these contigs will be mostly shorter. So, in order to resolve this, we first split the whole reads into several partitions to have lower coverage in each partition.

Secondly, by using low coverage, the resultant contigs will be mostly shorter than a higher coverage and have more gaps. In order to resolve this, we merge or assemble all contigs that produced from the assemblies of each partition along with all the reads again. This would recover any gaps due to low coverage assembly. Moreover, assembling contigs that were produced from all partitions will add support in selecting the common contigs (more likely to be true contigs) and in filtering out any redundant or false (partial or full) contigs. Moreover, the more corrected contigs we input to the re-assembly step the better assembly we expect.

Due to the nature of the process of partitioning the reads and then assembling the results, as well the fact that we may have several levels of partitioning especially for large genomes. This led us to the notion of “Hierarchical genome assembly”.

## Methods

In our study, we used four Illumina MiSeq dataset and three Illumina HiSeq datasets which were used in the GAGE-B paper [20].

■■■ **Input data:** The datasets are for four Bacterial genomes. Table 1 shows the descriptions of the genomes and the datasets.

**Table 1:** Descriptions of the bacterial genomes and sequence reads that were used in this paper. All data sets are paired-end reads

Dataset	Genome	GC content	Sequencing	Read	Fragment	Avg.	#
	size (Mb)	(%)	technology	length (bp)	length (bp)	Coverage	Proteins
Bacillus cereus ATCC 10987	5.4	35	MiSeq	250	600	100x	6,014
Mycobacterium abscessus 6G	5.1	64	HiSeq	100	335	115x	4,992
Mycobacterium abscessus 6G	5.1	64	MiSeq	250	335	100x	4,992
Rhodobacter sphaeroides 2.4.1	4.6	69	HisSeq	101	220	210x	4,474
Rhodobacter sphaeroides 2.4.1	4.6	69	MiSeq	251	540	100x	4,474
Vibrio cholerae CO1032(5)	4.0	48	HiSeq	100	335	110x	3,693
Vibrio cholerae CO1032(5)	4.0	48	MiSeq	250	335	100x	3,693
  As referred by GAGE-B, the data can be downloaded from the Sequence Read Archive at NIH’s National Center for Biotechnology Information using the following SRR accession numbers. *R. sphaeroides* MiSeq: SRR522246, HiSeq: SRR522244. *M. abscessus* MiSeq: SRR768269, HiSeq: SRR315382. *V. cholerae* MiSeq: SRR769320, HiSeq: SRR227312. *B. cereus* MiSeq data were downloaded from the Illumina website. GAGE-B then down-sampled the data to collect 250 × coverage with HiSeq data and 100 × coverage with MiSeq data. After that, they cleaned the raw data by removing adapter sequences and trimming the reads based on q10 quality. Both the raw (down-sampled) and the cleaned dataset are available at GAGE-B website http://ccb.jhu.edu/gage_b, and they are the datasets that were considered in this paper.
  All tested genomes in this paper have multiple chromosomes and/or plasmids. *V. cholerae* has two chromosomes, *B. cereus* and *M. abscessus* have one chromosome and one plasmid, and *R. sphaeroides* has two chromosomes and five plasmids. In order to compute the correctness of assemblies, we used the following strains as reference genomes: *B. cereus* ATCC 10987 (GenBank accession numbers NC_003909, NC_005707), *M. abscessus* ATCC 19977 (NC_010394, NC_010397), *R. sphaeroides* 2.4.1 (NC_007488, NC_007489, NC_007490, NC_007493, NC_007494, NC_009007, NC_009008), and *V. cholerae 01 biovar eltor str. N16961* (NC_002505, NC_002506).
■■■ **Assemblers:** We tested our method using eight open source genome assemblers, that were also tested in GAGE-B:
  Abyss v1.5.1, Cabog v7.0, Mira v4.0.2 [21], MaSuRCA v2.2.1, SGA v0.10.13, SoapDenovo2 v2.04, SPAdes v3.0.0, and Velvet v1.2.10.
  In order to describe the methods, we will use metrics that were used in QUAST tool [22] as it has been a common and accurate tool in evaluating and analyzing assembles’ results. Namely, we will use the following metrics *Number of contigs*, *N50*, *NA50*, *NG50*, *NGA50*, *Genome fraction (%)*, *Duplication ratio*, *Global misassemblies*, *Local misassemblies*, *# mismatches per 100 kbp (MP100K)*, *# indels per 100 kbp (IP100K)*, and *# Unaligned length*. For the descriptions of these metrics we refer the reader to QUAST [22], as well, we added their descriptions into Additional file 1.

### Hierarchical genome assembly

**Fig. 1:**
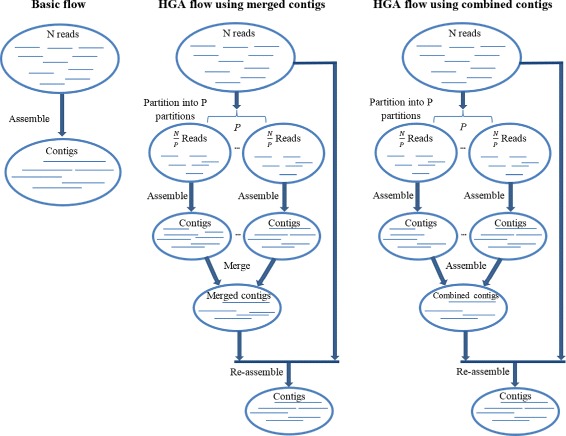
HGA flows: Flow diagrams represent the basic assembly flow and the hierarchical assembly flows. The basic flow represents the assembly of all reads in the dataset together. HGA flow using merged contigs represent the flow of partitioning the reads in the dataset into *p* disjoint partitions, then assembling each partition independently. After that the contigs of each partition’s assembly will be merged together. Lastly re-assemble the merged contigs with the whole reads. The only difference between HGA flow using merged contigs and HGA flow using combined contigs is that instead of merging the contigs of all partitions’ assemblies, they will be combined (assembled) together. In this paper, we used Velvet to assemble the contigs

Hierarchical genome assembly method includes the following steps. Firstly, all reads are partitioned into *p* disjoint partitions where *p* > 1. Then each partition is assembled independently. After assembling all partitions sequentially or in parallel, all the partitions’ assemblies will be assembled together to form *combined contigs*, or merged together to form *merged contigs*. Lastly, the merged contigs or the combined contigs will be re-assembled with the whole reads again. Figure 1 depicts a diagram of these steps.

This method will be mainly compared to the *basic assembly* process, as it’s shown in Fig. 1; that involves assembling the whole reads together and then output the assembly results. We denote the basic assembly as **B(kmer, c)** where *kmer* is the kmer length used in the assembly and *c* is the coverage of the reads.

#### Partitioning step

The main motivation of partitioning the reads set into smaller partitions is to gain lower coverage data. So that we expect to obtain a graph with less complexity, as a result, resolving the assembly’s ambiguities will be more efficient. It’s true that at higher coverage we may get longer contigs than lower coverage, but mostly these contigs will have more errors, in terms of global and local misassemblies, MP100K, IP100K, and unaligned contigs. In order to show this experimentally, we added into Tables S3–S9 (Additional file 1) a row that present the average values over all the partitions for each metric, so it can be compared with the basic flow results. The results show that, for most assemblers and for most genomes, the average values of local misassemblies, global misassemblies, MP100K, and IP100K over all the partitions are less than the values for the basic flow (the flow that is without partitioning the reads dataset), specially for HiSeq datasets where we have more and shorter reads, hence more graph complexities.

It’s *critical* for the steps of combining the contigs (contigs assembly) and the re-assembly step, to have contigs that are longer and with less errors in terms of local misassemblies, global misassemblies, MP100K, and IP100K. The results of these steps will be more efficient and accurate when they are given contigs that are more corrected and longer.

The first step of the method is to partition the reads set, by splitting the whole reads set (*N* reads) into *p* disjoint partitions. So, each partition has $\frac {N}{p}$ reads. After performing several experiments on finding how many partitions to produce, we observed that there is no constraints on how many partitions to produce, as long as we have ≥10*x* coverage for each of the partitions. So, in general, the partition’s coverage is the only constraint to be considered for this step, not how many partitions to be produced.

#### Contigs assembly (combining) step

After assembling all the partitions, we merge all partitions’ assemblies together to form the merged contigs, or assemble (combine) them together forming the combined contigs. Initially, we assembled the contigs of the partitions using minimus2 [23], but after the analysis of several experiments we found that minimus2 is actually misleading in combining contigs. Despite of the improvement in term of NA50 of minimus2’s results, there was *significant* increase in both *duplication ratio* and *misassemblies events*. This occurs because the input data are not short reads, but instead they are long reads (contigs). So, as minimus2 compute the pairwise alignment between all the reads (which are here contigs), this would mean that if we have two (or more) contigs that are true (aligned) but *not contiguous in the reference* and they share an x-mer (which may be repeat), then minimus2 will output these contigs as one contig. Moreover, one of these contigs may most likely again share x-mer truly or falsely with other contigs, as a result, this will lead to output the same contigs for multiple times which will eventually increase the duplication ratio. In addition, assembling true and not contiguous contigs will increase the number of global or local misassemblies. Hence, the improvement in the results of NA50 is mostly false positive.

It’s clear that string graph assemblers such as SGA would not work effectively on assembling contigs. Some contigs may start overlapping in the middle of other contigs and this is not covered by string graph by definition, as it computes the overlap at the ends of the reads (contigs in this case). So, we switched to assemblers that use the de Bruijn graph. Among all assemblers that are based on de Bruijn grapg and which take contigs as input data, we tested which assembler has the best contigs-only assembly results; we found Velvet is the best choice. Moreover, after several experiments of varying the kmer length used in running Velvet to assemble the contigs, kmer value of 31 as well as providing the expected coverage of the input data to Velvet to be as same as the number of partitions, led to the best contigs assembly results. The results of running Velvet as contigs assembler and the comparisons with the results of minimus2 were provided in Additional file 1: Table S11.

We denote the results of combining contigs as **C(kmer, p, c)**; where *p* is the number of partitions, *c* is the coverage of each partition, and *kmer* as the used kmer in the assembly of each partition. While we denote the resultant contigs from merging the partitions’ contigs as **M(kmer, p, c)**.

#### Re-assembly step

In this step, the merged or combined contigs will be reassembled with all the reads again. To accomplish this, we had to select an assembler that takes long sequences (contigs) as an input in assembling contigs, as well the assembler preferably should be based on de Bruign graphs. For these reasons SPAdes and Velvet were the convenient candidates. After testing both of them on two genomes, SPAdes produced better re-assembly results than Velvet; details of the tests and the comparison results are provided in Additional file 1: Tables S12 & S13. Hence, all re-assembly results in this paper were performed using SPAdes assembler. We denote this step as **HGA(kmer, contigs)**, where *kmer* is the kmer length that was used in the reassembly process, *contigs* is whether the merged contigs or the combined contigs.

So, in general, we have the following flows: B(kmer). HGA Preprocessing flows: M(kmer, p, c) and C(kmer, p, c)). HGA re-assembly flows HGA(kmer, M(kmer, p, c)) and HGA(kmer, C(kmer, p, c)).

Reassembling the reads with long sequences (contigs) has several advantages. Firstly, these contigs were produced not from assembling all the reads together but from combined or merged contigs of the assembly of different partitions. So, they are more corrected and refined in terms of errors, much longer, also they are not redundant contigs meaning that they were not already produced from the assembly of the *same* whole reads. Instead they were assembled from several disjoint subsets, hence they are structurally different as well as they were produced from a subset of the *same* reads that they will re-assemble with. Moreover, we experienced that re-assembling contigs, which were produced from some reads dataset, again with the same dataset will not improve the assembly and even it deteriorate the new assembly. Secondly, reassembling long sequences (contigs) may lead to have different connected components to get connected. Finally, during the path finding process this may increase the chances of selecting the true paths by traversing the longest path which induced from having long sequences in the input data.

To further explore and justify the advantages of the re-assembly step we performed a simple test on a real dataset of *M. abscessus* bacteria (the dataset is used in this study and is described in the next section). We assembled the real HiSeq dataset of *M. abscessus* along with the genome of *M. abscessus* itself, using SPAdes assembler and a range of kmer lengths of 21, 31,.., 91. The NA50 result of the assembly at kmer =91 was 99 % of the length of the genome of *M. abscessus*. This indicates that the assembly of contigs with the reads will be computationally indeed effective and could lead to an optimal assembly, but the more correctness of these contigs the better the re-assembly results.

## Results

Before of all, some assemblers namely Abyss, Mira, MaSuRCA, SGA, SPAdes, and Velvet are newer versions than the versions used in GAGE-B. For this we made the following. Firstly, the assemblies’ results reported in GAGE-B were compared in this paper. Secondly, as these assemblies were using older versions, we did run the basic flow using the new versions of all assemblers using kmer lengths (or overlap lengths for SGA) of 21, 31, …, 91 for HiSeq datasets, and 21, 31, …, 101 for MiSeq data sets. Then, we reported the assembly that has the highest N50. Moreover, MIRA and CABOG assemblers take no kmer or overlap values as parameters, so their single assembly is reported rather than the maximum assembly results over the kmer (or the overlap value) range.

Each dataset was assembled by each assembler as paired end reads. We followed the run commands for each assembler that were provided in the GAGE-B’s Supplementary document, which are also in Additional file 1. For the basic assembly flow, we used for all assemblers, except for MIRA and CABOG, kmer lengths (or overlap lengths for SGA) of 21, 31,…, 91 for HiSeq data sets, and 21, 31,…, 101 for MiSeq data sets. For HGA flows, we split the reads into 2, 4 and 8 partitions, we didn’t split the reads more than that because the coverage of each partition will be very low (<10*x*) to assemble. Then, we assembled each partition independently using the same kmers set that were used in the basic flow (note that this step can be done in parallel). For instance, we split the reads data sets into 4 partitions then we assembled each partition using kmer lengths = 21, 31, …91 for HiSeq data sets, and 21, 31, …101 for MiSeq data sets.

During the next step of combining the contigs, at first we merged the contigs of all partitions together to form the *merged contigs*. As well, we combined (assembled) them to form the *combined contigs* using Velvet with kmer value of 31 and specifying to Velvet the expected coverage to be the number of partitions. For the re-assembly step, we used Velvet and SPAdes for this purpose as they take long sequences as inputs. The re-assembly results using SPAdes outperformed Velvet’s results. So, all runs on this paper were using SPAdes in the re-assembly step.

For experimental purposes and for the HiSeq datasets we have the following combinations (1, 21), (1, 31)…(1, 91), (2, 21), (2, 31)…(2, 91), (4, 21), (4, 31)…(4, 91), (8, 21), (8, 31)…(8, 91); for each combination we run the re-assembly step using kmer lengths = 21, 31,…91 with the merged or combined contigs which were produced from the combinations. Similarly, we have the following combinations (1, 21), (1, 31)…(1, 101), (2, 21), (2, 31)…(2, 101), (4, 21), (4, 31)…(4, 101), (8, 21), (8, 31)…(8, 101); for each combination we run the re-assembly step using kmer lengths = 21, 31,…101 with the merged or combined contigs which were produced from the combinations.

### Assemblies results

**Fig. 2:**
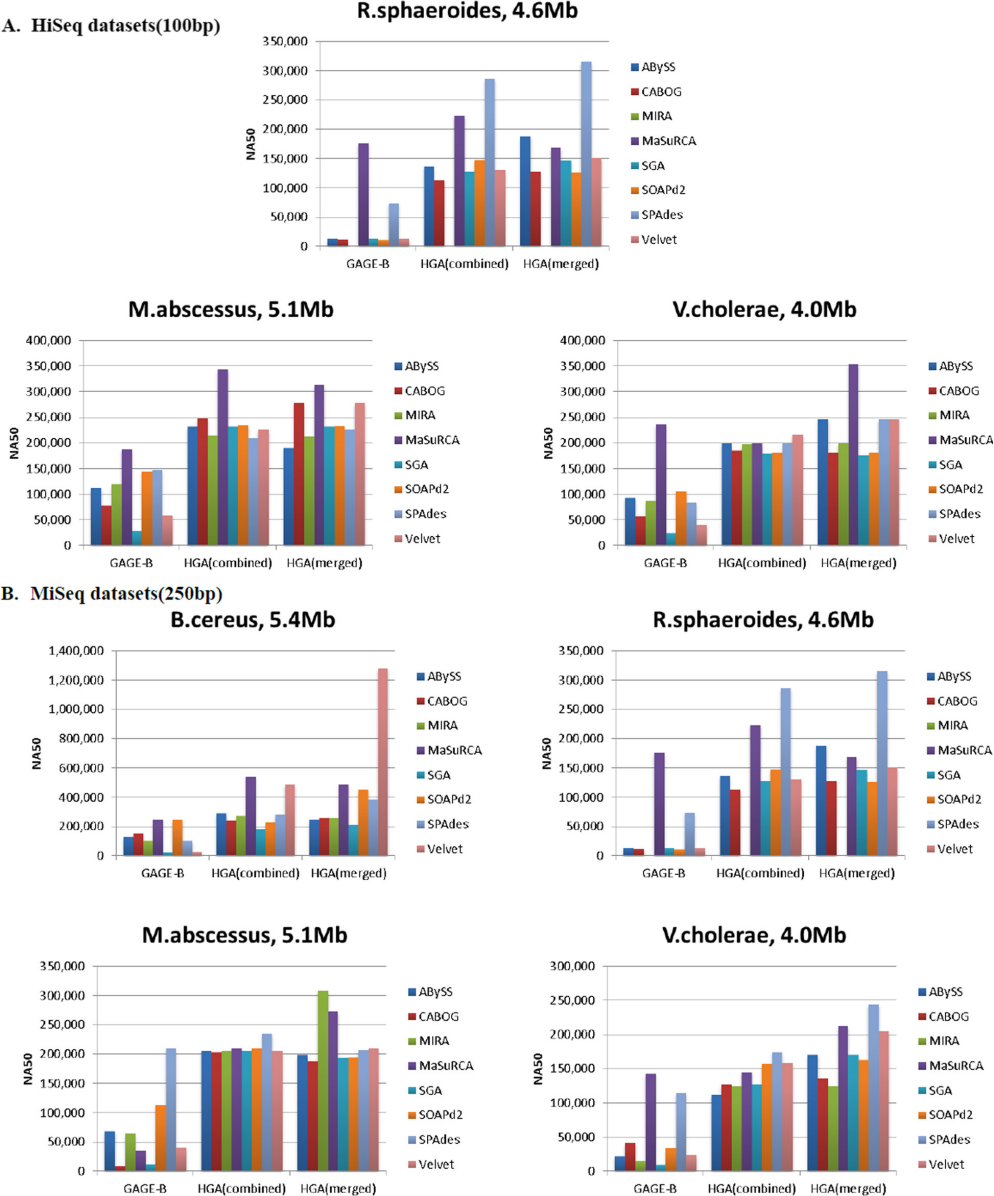
NA50 Results: NA50 (corrected N50) correspondent to the assembly with the highest N50 results for GAGE-B and HGA assemblies

Figure 2 shows the results of the assemblies using both flows of HGA method compared to GAGE-B’s best reported results. We compared the results based on the corrected N50 (NA50) correspondent to the *highest N50* result, which is a common metric that has been used to evaluate the accuracy of the assembly outputs for *de novo* genome assembly. GAGE-B reported the evaluation results of their assemblies, but as QUAST had a newer version and as the genes GFF files may be updated, we downloaded the assemblies from GAGE-B website and assessed them along with the assemblies of HGA using the same versions of QUAST and the latest genes GFF files, for consistency.

HGA method has two main flows, HGA(kmer, M(kmer, p, c)) and HGA(kmer, C(kmer, p, c)) as illustrated in Fig. 2. We abbreviated them as HGA(merged) and HGA(combined), respectively. The full results of the main flows, preprocessing flows, and GAGE-B results for different metrics are available in Additional file 1: Tables S3–S9.

Note that the results using the same assembler are always improved using HGA method. As well as the maximum result of HGA flows across different genomes is larger than the maximum of GAGE-B’s reported results. Mostly SPAdes, MaSuRCA and Velvet have the best HGA assembly over the rest assemblers. We can see that the results using HGA were improved significantly for both HiSeq and MiSeq datasets. For *R. sphaeroides* HiSeq datasets, it improved from 177 Kbp (maximum of GAGE-B results) to 315 Kbp (maximum of HGA results). Likewise, it improved from 189 Kbp to 344 Kbp for *M. abscessus*. As well from 236 to 354 Kbp for *V. cholerae*.

MiSeq datasets showed a considerable improvement. *B. cereus* results improved from 247 to 1,276 Kbp, *R. sphaeroides* from 143 to 246 Kbp. The improvement for *M. abscessus* was from 210 to 309 Kbp. lastly, *V. cholerae* results increased from 247 to 356 Kbp. Moreover as a result of these improvements the number of founded Proteins did increase, which are plotted in Additional file 1: Figure S1.

How does HGA method contributes in improving the assembly process is described in the following explanations. Assembling the whole reads together using long kmer size, which is effective in resolving repeats in the genome, will decrease the overlapping between reads due to errors in the reads; hence, the connected components in the graph will be more and eventually the resultant contigs will be shorter. Now, let’s assume that such an assembly involves correct contigs (with no errors); then, firstly such contigs will connect different connected components. Secondly reads will overlap with such contigs. Thirdly, at a branching point, the branch that is connected with contigs will have more weights, hence this will aid assemblers in selecting the true path during the path finding process. In conclusion, this will improve the overall assembly process by firstly assembling repeats regions more correctly; and secondly, producing longer contigs. The problem now is to produce contigs that are correct or as correct as possible. Furthermore, the process is also true in a reverse way meaning if the contigs are not highly corrected but they already assembled repeats regions then assembling such contigs with the whole reads using short kmer size will also improve the assembly results as is shown in some results in Tables S3–S9 (Additional file 1); since repeats were already resolved in the inputted contigs and performing the assembly using short kmer size will be more feasible to correct errors in the reads/contigs.

In order to produce contigs that are most corrected, note that without even partitioning the whole reads, assembling the whole reads using short kmer, then re-assemble the resultant contigs with the whole reads again led to improve the assembly results. As an example, Table S5 (Additional file 1) shows that the result of assembling 1 partition (the whole reads) using kmer size 21, then re-assembling the resultant contigs with the whole reads using kmer size 41, was better than 2, 4, and 8 partitions, and even it was the highest compared to all other methods and assemblers. Moreover, beside the fact that short kmer sizes is better in correcting the reads’ errors than longer kmers sizes, we observed experimentally Tables S3–S9 (Additional file 1) that contigs which were produced from lower coverage have less errors in terms of local misassemblies, global misassemblies, MP100K, and IP100K in most assemblies. So we decided to apply such an approach as an attempt, along using short kmer size, to produce more corrected contigs to be inputted to the re-assembly step.

Partitioning the whole reads has mainly two advantages. Firstly, when building a graph using a set of reads, then all errors in all reads will create error-related complexities (such as false branching, tips, and bubbles) in the graph. Now, building a graph using an *x* partitions of the reads set, although all errors in the reads will still induce error-related complexities, but in this case they will be distributed into *x* graphs. Hence, in each of the x graphs we will have less error-related complexities compared to the graph that is built using the whole reads set. This will aid the assembler to apply their algorithms to resolve error-related complexities more feasibly in each graph, ultimately contigs that result from all partitions will contain less errors. Note that complexities that usually are induced by e.g. repeats or SNPs will however still be presented.

Secondly, with a single partition’s assembly, assemblers are compelled to produce contigs of coverage of 1x, in order to avoid redundancy in the assembly result. So at a branching point (node in the graph with indegree =1 and outdegree ≥ 1), assemblers must traverse single and the *best* possible branch, given that it might be heuristically false positive, hence false contig production. Now, when partition the whole reads into multiple partitions, firstly the resultant contigs of all partitions’ assemblies will be of coverage *P*x not only 1x, where P is the number of partitions. Secondly, it’s highly possible that, at the same branching point, different branches will be traversed in the assembly of different partitions. Hence, as more branches will be traversed, the true branch is more likely to be traversed, hence a true contig are more likely to be produced. Thus, the resultant contigs of all partitions will carry more possibilities of the true contigs. This latter analysis also explains the reason that the re-assembly process using merged contigs showed better results than the combined contigs (that is of 1x coverage) as well better than the re-assembly using the contigs that were produced from 1 partition.

### Multi-kmers assembly

Some assemblers use multi-kmers for assembly such as SPAdes. Firstly, SPAdes’ results that were provided by GAGE-B were already *optimized* over multiple kmers. Moreover, we did run SPAdes using two kmers, kmer that were used in the preprocessing step and kmer that were used in the re-assembly step; in order to test if there is an improvement using multi-kmer assembly by using the same kmers that were used in the steps of HGA method. Some results showed slight improvements over the single kmer assembly, but weren’t even close to the improvements made by HGA methods, even some results were worse. This can be explained as the complexity of the de Bruijn graph using multiple kmers would not be reduced and even it may increase compared to the complexity of de Bruijn graph of a single kmer. Also, it’s hard to find the combination of kmer sizes that will lead to the best assembly, as this process involve costly enumeration process and running trial for each tested combination. HGA methods utilizes the use of multi-kmers, not *collectivity*, but in phased manner.

### Impacts of the contigs correctness & length in the re-assembly process

The correctness and the length of the contigs that are re-assembled with the whole reads are critical to improve HGA assembly results. To investigate that and as an example, we could notice, Tables S3–S9 (Additional file 1), that MaSuRCA HiSeq assemblies were ones of the highest in terms of the metric MP100K compared to the other assembler. But in terms of local and global misassemblies MaSuRCA is one of the assemblers that had the lowest. Moreover, as an evaluation and for the re-assembly step, the lower MP100K and IP100K would produce better results than lower local or global misassemblies; since MP100K and IP100K are measured per 100 kbp. As a result, for a genome of size 5 Mbp and MP100K = 5, this will induce 250 mismatches, where local or global misassemblies values are usually in average of tens. The MP100K for MaSuRCA’s HiSeq assembly, basic flow for of *R. sphaeroides* is 47.5 (∼2,090 mismatches = genome size x 10 x genome fraction of the assembly x MP100K); for *M. abscessus* is 21.2 (1,080); and for *V. cholerae* is 23.8 (930). Such a large number of mismatches in the contigs will induce less overlaps between the reads and these contigs during the re-assembly process. These values were reduced by the partition step to 22.8 (1,000), 6.7 (340), and 14.8 (570), respectively; and were further reduced/increased in the combining step (assembling the contigs using velvet) to 18.3 (800), 5.6 (280), and 17.2 (670) respectively. This explains why the re-assembly process was better using the combined contigs rather than the merged contigs for MaSuRCA’s HGA results; except for *V. cholerae* which correlates with the fact that the combining step didn’t decrease the errors and it actually increased them. It’s worth to mention that although the HiSeq contigs (combined and merged) by MaSuRCA showed more errors in terms of MP100K, these contigs were more contiguous (longer) compared to the other assemblers’ resultant contigs. As the longer contigs that get re-assembled using HGA method the better HGA assembly results. HGA results using MaSuRCA were among the best results for MiSeq assemblies and the best for two HiSeq assemblies *M. abscessus* and *V. cholerae*.

In conclusion, this analysis suggests that resolving the causes of MP100K and IP100K more carefully at least during the steps, before the re-assembly process, namely partitions assembly step and/or the contigs combining; will lead to improve the results of the re-assembly step hence the overall assembly results.

### Testing error-free reads

As noted in the Discussion section, the contigs in the partitioning and combining steps were expected to obtain as much as possible correction/refining before re-assembling them again with all the reads. This explains the improvement in the HGA results. To further justify that we simulate reads with no errors from *M. abscessus* genome with the same coverage of the real dataset, then we run HGA methods on those reads. The assemblies results of HGA flow of combining contigs and HGA using combined contigs showed no improvement over the basic flow, while it wasn’t the same case with the real dataset which involves errors in the reads and the improvements were much more significant.

This indicates that HGA methods were able to correct more errors in the reads than the basic flow. Moreover, even with error free reads, HGA using merged contigs showed an improvement over all the other HGA methods and the basic flow. This again supports the point that assembling contigs at a higher coverage with the whole reads is better than combining contigs first and then assemble the combined contigs (usually coverage of 1x) with the whole reads. The test results were provided in Additional file 1: Table S10.

### Partitions-kmer to re-assembly-kmer relations

**Fig. 3:**
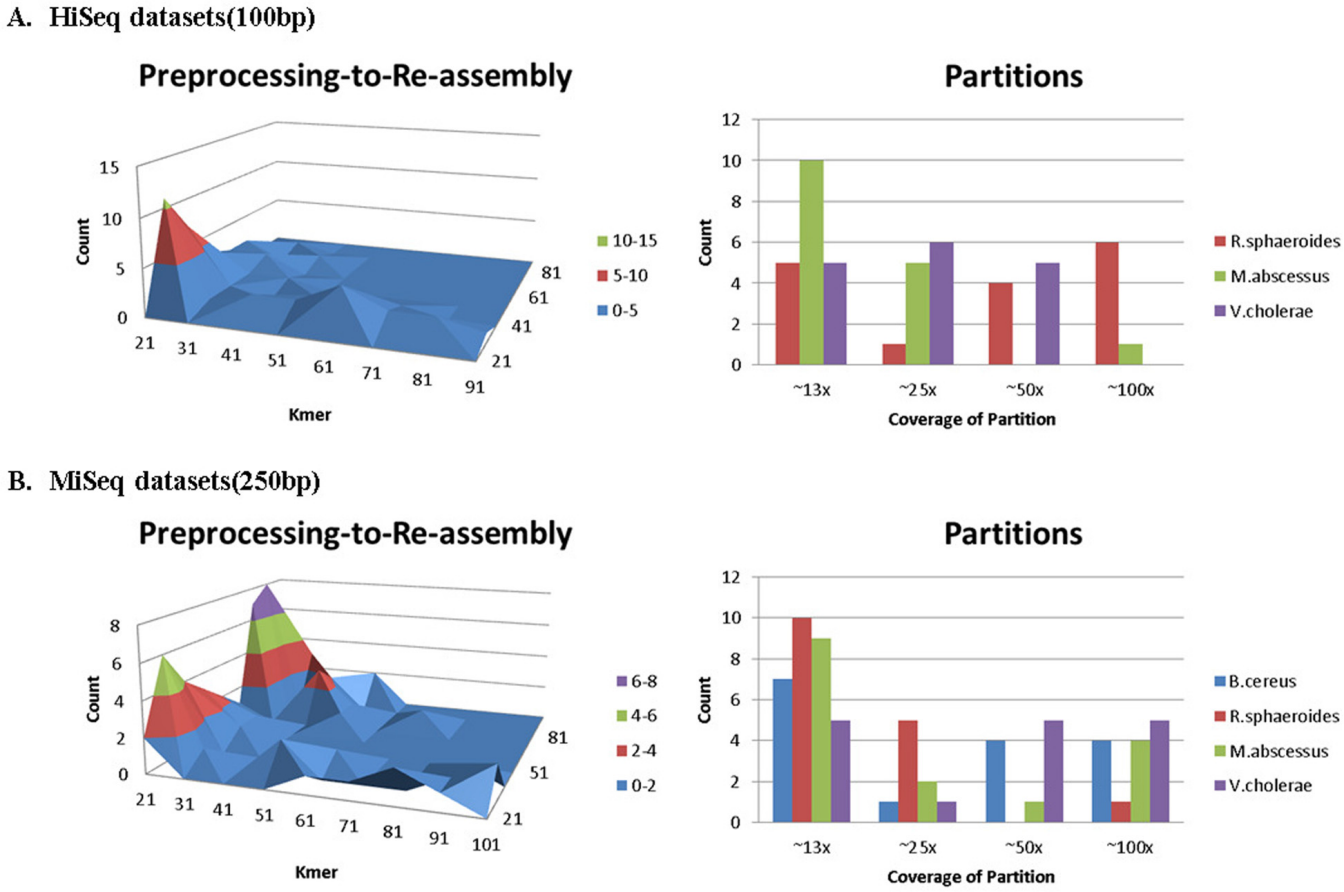
Partitions-kmer to Re-assembly-kmer Relations: As there are 16 highest results (2 flows for 8 assemblers); we built two plots, the first plot is a surface chart that shows the count of combination of kmers (preprocessing kmer to re-assembly kmer) that were applied by the 16 highest results. As well the second plot shows the counts of partitions that were applied by the 16 highest results

As HGA method involves two assembly steps, each assembly step use same or different kmer size, kmer that is used in the preprocessing step and kmer in the re-assembly step. Moreover, as we tested all combination of the two kmers then we selected the assemblies of the highest N50 results (8 highest assemblies for 8 assemblers for both combined and merged flows), we plotted in Fig. 3 out of these 16 assemblies how many times each kmer were used during the preprocessing step and during the re-assembly step, in order to observe correlations.

We could observe that for most of the best assembly results on MiSeq data, using short kmers (21 and 31) in the preprocessing step and long kmers (61, 71, and 81) in the re-assembly step, showed better assembly results. While, for HiSeq data, short kmers (21 and 31) in the preprocessing step and medium kmers (31, 41, and 51) in the re-assembly step, showed higher assembly results. Figure 3 shows the plots of the kmer sizes that were used in the preprocessing and the re-assembly steps, as well how many times each kmer were observed. It’s clear to notice that 21 and 31 were dominant during the preprocessing step. This can be justified as the assembly process using short kmer sizes leads to more corrected contigs and as these contigs are inputs to the next step (re-assembly step); the more corrected contigs, the more likely to lead to better assembly results. For the re-assembly step, medium to long kmers were dominant, this can be explained because with such lengths of kmers, resolving the complexities induced from repeats is prior, specially when the corrected contigs collaborate in reducing the complexities/computation that usually induced from errors. So, the assemblies using such a combination were mostly the highest in N50 results.

Adopting short kmer as a stereotype in the preprocessing step and medium-long kmer in the re-assembly step might help in eliminating the common confusion of what kmer length (short or long) to apply in the assembly. In addition, it helps to avoid the need to run some preprocessing tools like KmerGenie [24] that is used to find the best kmer size to be used in the assembly process, or trying several assembly trials then selecting the best assembly results, or the need to run assembly using multiple kmers which cost more space and time and showed no significant improvement compared to HGA method.

Lastly, we plotted also the most frequent number of partitions that were used among the highest assemblies results as shown in Fig. 3. We concluded that more partitioning (less coverage) was most frequent. This can be explained, again, as with low coverage the graph is less complex, hence more corrected contigs to be inputted into the reassembly step.

## Discussion

We first denote the following notations. *R* as the length of the reference genome, *L* as the length of the input reads, *C* as the expected genome coverage by the reads, *K* as the size of the kmer, and *Ideal case* as the case where there is no errors in the sequencing reads, the length of any repeat in reference genome is > L, and the reads were sequenced uniformly.

■■■ *Analysis 1*: In the ideal case and when *C* for instance ≥ 100 ×, building the de Bruijn graph using short kmer size or long kmer will lead to the same and optimal assembly; the only difference would be that by using large kmer the assembly will need less memory and will be faster. For a non-ideal case, reads have errors and this induces tips, bubbles, and false branching. Repeats in the genome with length less than *L* will induce cycles, bulges, false branching, and also tips and bubbles. Non-uniform coverage results in varying kmer counts and reduces overlapping between the reads, hence this will create gaps and increases the number of components in the graph.
  Now, there is a tradeoff between using long or short kmer sizes. Long kmer size leads to less false branching, better resolution of repeats, and less cycles; but less error correction and detection and less overlapping between the reads (more components and gaps). On the other hand, short kmer sizes outputs the opposite namely, more false branching, more cycles, and less repeats resolution; but errors correction and detection will be more effective and the graph would have less components and gaps.
■■■ *Analysis 2*: When we have reads of high coverage, the chances of not covering some regions of the genome will be less and the resultant contigs from these reads will be longer but will lead to a more complex graph than a graph built from lower coverage reads. Hence, the algorithms of errors correction and path finding that are applied by the assemblers will be less effective at high coverage. This is the main motivation behind partitioning the whole reads to low coverages reads sets. In order to resolve the effects of having more gaps in the assemblies of the partitions compared to the assembly of the whole reads, re-assembling the contigs with the whole reads will resolve such an issue and utilize the higher coverage of the original whole reads set.

## Conclusion

By using HGA method, there is a significant improvement in the assembly quality, based on N50 and corrected N50 metrics, using different assemblers on different genomes. HGA applies hierarchical approach where the assembly process starts with lower coverage assembly to get more corrected but shorter contigs results, then assembling these contigs with all reads again to gain longer contigs results.

Moreover, HGA eliminates the hardness and the trade-off of using short, long, or multiple kmer lengths, where short kmers are better to correct the reads and long kmers are better to resolve the repeats. For HiSeq dataset (reads lengths of ∼100 bp) and for coverage of ∼100x, partitioning the datasets into disjoint partitions each of coverage of ∼12x and ∼25x. Then, assembling each partition using kmer sizes of 21–31. Next, merge or combine (assemble) all partitions’ contigs together. After that, re-assembling the resultant contigs with the whole reads using kmer values of 31–51; led to significantly better assembly results. For MiSeq dataset (reads lengths of ∼250 bp) and for same coverage of ∼100x, following the same settings as HiSeq’s but with re-assembling the resultant contigs with the whole reads using kmer values of 61–81 led to significant results.

## Availability and requirements

**Project name:** HGA v1.0.0.**Project access:**https://github.com/aalokaily/Hierarchical-Genome-Assembly-HGABJW60577Assembly-HGA.**Operating system:** Tested on Linux/Ubuntu OS, AMD Opteron(tm) 2.4 GH, 256 GB Memory, 64 core. Programming language: Python 2.7.6.**Other requirements:** SPAdes v3.0.0, Velvet v1.2.10. Velvet must be installed as indicated in Additional file 1.**License:** GNU GENERAL PUBLIC LICENSE.**Any restrictions to use by non-academics:** None.

## Additional file

Additional file 1 Supplementary tables, experiments, and descriptions. The file contains a detailed results of the assembly of the genomes and assembler which were used in this research. Also, the results of some experiments that were performed to explain the method and the improvement showed in the manuscript. In addition, the file includes a descriptions of the datasets and the metrics which were considered in the manuscript. Lastly, the commands that were used by the different assemblers to run the assemblies. (DOCX 600 kb)

## Abbreviations

HGA: hierarchical genome assembly
NGS: next-generation sequencing
MP100K: # mismatches per 100 kbp
IP100K: # indels per 100 kbp
