# Supplementary material for: HGA: denovo genome assembly method for bacterial genomes using high coverage short sequencing reads
Source: BMC Genomics. 2016 Mar 5;17:193. doi: 10.1186/s12864-016-2515-7 (PMC4779561; doi:10.1186/s12864-016-2515-7)
Supplement: Additional file 1 — Supplementary tables, experiments, and descriptions. The file contains a detailed results of the assembly of the genomes and assembler which were used in this research. Also, the results of some experiments that were performed to explain the method and the improvement showed in the manuscript. In addition, the file includes a descriptions of the datasets and the metrics which were considered in the manuscript. Lastly, the commands that were used by the different assemblers to run the assemblies. (DOCX 600 kb) [file 12864_2016_2515_MOESM1_ESM.docx]

**Supplementary Tables, Figures, and Text**

**Supplementary Table 1:** Additional dataset statistics.

|  | **Mean insert size** | **Std. Dev. of insert size** | **Genome size (bp)** | **Bases covered (bp)** | **% Covered** |
| --- | --- | --- | --- | --- | --- |
| **B. cereus MiSeq** | 600 | 60 | 5,432,652 | 5,431,532 | 99.98 |
| **M. abscessus HiSeq** | 335 | 35 | 5,090,401 | 5,060,999 | 99.42 |
| **M. abscessus MiSeq** | 335 | 35 | 5,090,401 | 5,021,173 | 98.64 |
| **R. sphaeroides HiSeq** | 220 | 25 | 4,565,960 | 4,564,190 | 99.96 |
| **R. sphaeroides MiSeq** | 540 | 60 | 4,565,960 | 4,560,486 | 99.88 |
| **V. cholera HiSeq** | 335 | 35 | 4,033,464 | 4,009,169 | 99.40 |
| **V. cholerae MiSeq** | 335 | 35 | 4,033,464 | 3,879,892 | 96.19 |

**Supplementary Table 2:** Datasets chosen, cleaned or raw, for each genome and each assembler. As reported in the GAGE-B study, some assemblers produced better results using the cleaned data than using the raw data, and others not; so we followed their recommendations on which datasets to use for each assembler and for each genome.

|  | **ABySS** | **CABOG** | **MIRA** | **MaSuRCA** | **SGA** | **SOAPdenovo** | **SPAdes** | **Velvet** |
| --- | --- | --- | --- | --- | --- | --- | --- | --- |
| **B. cereus -MiSeq** | raw | clean | raw | raw | raw | raw | clean | raw |
| **M. abscessus -HiSeq** | clean | clean | raw | raw | clean | clean | clean | clean |
| **M. abscessus -MiSeq** | clean | clean | clean | clean | clean | raw | clean | raw |
| **R.sphaeroides - HiSeq** | raw | raw | raw | raw | raw | raw | clean | raw |
| **R.sphaeroides - MiSeq** | raw | raw | raw | raw | clean | clean | clean | clean |
| **V. cholerae -HiSeq** | clean | clean | raw | raw | clean | clean | clean | clean |
| **V. cholerae -MiSeq** | clean | raw | clean | clean | clean | raw | clean | clean |


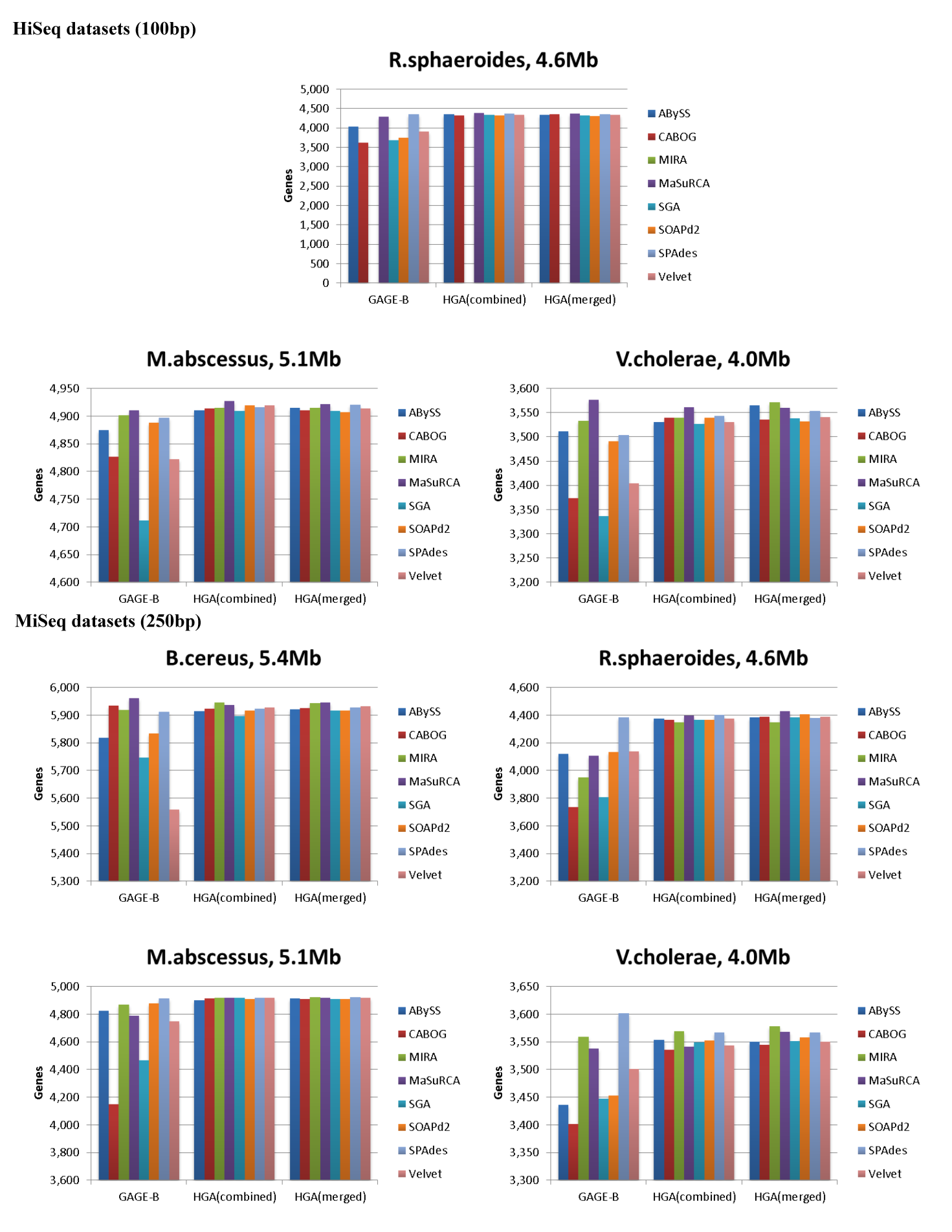


Supplementary Figure 1: Number of genes identified by QUAST in CAGE-B and HGA assemblies

# Detailed results for all assemblies

Firstly, we state the description of QUAST’s metrics that are used in the paper and here also:

**Number of contigs** is the number of contigs in the assembly. **N50** is the length for which the collection of all contigs of that contig’s length or longer covers at least 50% of the total length of contigs in the assembly; **NA50** is computed by firstly obtaining blocks, which resulted from breaking the contigs at misassemblies events (after aligning the contigs); after that compute the NA50 out of the blocks that were aligned to the reference, similarly of computing N50. **NG50** and **NGA50** are computed like N50 and NA50 respectively, but not out of the total length of contigs in the assembly, instead out of the reference length, here the reference genome is known in advance. **Genome fraction (%)** which is the percentage out of the reference’s length, if bases that aligned to the reference. **Duplication ratio** which is the total number of aligned bases in the assembly divided by the total number of aligned bases in the reference; if the assembly contains many contigs that cover the same regions of the reference, the duplication ratio will be larger than 1.

In order to assess and evaluate the graph complexity as well as comparing different assemblies’ results, we used the following metrics. **Global misassemblies** which is the number of positions in the contigs that satisfy one of the following criteria: the left flanking sequence aligns over 1 kbp away from the right flanking sequence on the reference, flanking sequences overlap on more than 1 kbp, or flanking sequences align to different strands or different chromosomes.

**Local misassemblies** which is the number of positions in the contigs that satisfy one of the following criteria: Two or more distinct alignments cover the breakpoint, the gap between left and right flanking sequences is less than 1 kbp, or the left and right flanking sequences both are on the same strand of the same chromosome of the reference genome. Global misassemblies indicates false assembly in the long ranges of the reference and caused by assembling regions that are far away from each other. This may mostly occur in the graph by false connection between two or more of connected components or false branching in the same connected component, due mainly to long repeats. Local misassemblies in the other hand, indicate false assembly between close regions and this occur in the same connected component, due mainly to errors. More global and local misassemblies indicate more complexity in the graph whether within connected components or among connected components.

In addition we used **# mismatches per 100 kbp,** and **# indels per 100 kbp**. These metrics assess the ability and efficiency of the assembler to correct errors (mismatches or indels) after assembling the reads. Last metric is **# Unaligned length** which is the total length of contigs (or parts of contigs) that fail to align to the reference genome. This metric evaluate the length of false assemblies results.

For more details on the metrics reported in Tables 3-13 please see <http://quast.bioinf.spbau.ru/manual.html#sec3.1>

Tables 3-9 highlight the best results as the following: for each dataset, the best results for each metric for the results of *contigs assembly* in the results that were reported by GAGE-B are colored in blue, similarly the best results for each metric for the results of *contigs assembly* in the results of HGA methods are colored in blue; the best results for each metric overall results of GAGE-B and HGA methods are in bold. Same highlighting tactic are applied for the *scaffolding results*, but instead of blue the highlights were in green.

Also, Tables 3-9 present QUAST results for the following assembly flows:

- *B(k):* gives the results of running the corresponding assembler using kmer size *k*.
- *AP(k,p,cov)*: gives the *average* value for each metric over *p* assemblies obtained by independently running the corresponding assembler with kmer size *k* on *p* disjoint parts, each with average read coverage *cov* (equal to 1/p of the original reads coverage).
- *C(k, p, cov):* the results of combining (using Velvet with kmer size 31) the assemblies obtained by independently running the corresponding assembler with kmer size *k* on *p* disjoint parts, each with average read coverage *cov*.
- *M(k, p, cov)*: is the result of only merging the contigs obtained by independently running the corresponding assembler with kmer size *k* on *p* disjoint parts, each with average read coverage *cov*.
- *HGA(k1, C(k2, p, cov)):* the results of re-assembling (using SPAdes assembler) the whole reads using kmer size *k1*, with the contigs obtained from the *C(k2, p, cov)* flow.
- *HGA(k1, M(k2, p, cov))*: the results of re-assembling (using SPAdes assembler) the whole reads using kmer size *k1*, with the contigs obtained from the *M(k2, p, cov)* flow.
- GAGE-B(k) gives the results obtained from the assemblies that we downloaded from <http://ccb.jhu.edu/gage_b>, where *k* is the kmer size used for assembly.

For each HGA flow the reported assemblies’ results were selected based on the highest N50 assembly over all combinations of kmers sizes as described in the main paper. The reported value of *k* for GAGE-B flows represents the kmer size(s) used in the construction of the de Bruijn graph by ABySS, MaSuRCA, SOAPD2, SPAdes, and Velvet, respectively the minimum overlap length used by SGA, similarly optimized to maximizing N50 as described in the GAGE-B paper.

**Supplementary Table 3:** 250bp MiSeq read assemblies of *B. cereus*, with reference genome size of 5,432,652bp and 6,014 genes. For metric and assembly flow descriptions see beginning of section.

| **Assembler** | **Type** | **Flow** | **N50** | **NA50** | **NG50** | **NGA50** | **Genome** | **Duplica-** | **#** | **# mis-** | **Local mis-** | **# mismatches** | **# indels** | **Unaligned** | **#** |
| --- | --- | --- | --- | --- | --- | --- | --- | --- | --- | --- | --- | --- | --- | --- | --- |
|  |  |  |  |  |  |  | **fraction** | **tion ratio** | **contigs** | **assemblies** | **assemblies** | **per 100KB** | **per 100KB** | **length** | **Genes** |
| **ABySS** | **Basic flow** | B(81, 100x) | 215,752 | 215,752 | 215,752 | 215,752 | 99.2 | 1.016 | 64 | 4 | 55 | 6.5 | 5.4 | 0 | 5,878 |
|  | **HGA Preprocessing** | AP(91, 2, 50x) | 257,449 | 257,161 | 257,449 | 257,161 | 99.1 | 1.024 | 68 | 5 | 110 | 6.5 | 5.4 | 4,138 | 5,843 |
|  |  | C(91, 4, 25x) | 111,188 | 110,839 | 111,188 | 110,694 | 98.2 | 1.017 | 149 | 0 | 112 | 96.7 | 11.8 | 2,309 | 5,711 |
|  | **HGA re-assembly Contigs** | HGA(101, C(51, 4, 25x)) | 287,730 | 287,730 | 287,730 | 287,730 | 98.7 | 1.001 | 79 | 3 | 27 | 48.6 | 6.9 | 3,325 | 5,914 |
|  |  | HGA(81, M(41, 8, 12x)) | 515,336 | 246,654 | 515,336 | 246,654 | 98.9 | 1.002 | 61 | 5 | 25 | 16.8 | 4.2 | 3,939 | 5,922 |
|  | **GAGE Contigs** | GAGE-B(49) | 130,570 | 130,570 | 130,570 | 130,570 | 98.6 | 1.006 | 115 | 2 | 25 | 6.7 | 4.5 | 2,548 | 5,819 |
|  | **HGA re-assembly scaffolds** | HGA(101, C(51, 4, 25x)) | 309,910 | 287,730 | 309,910 | 287,730 | 98.7 | **1.001** | 76 | 3 | 30 | 48.6 | 6.9 | 3,325 | 0 |
|  |  | HGA(81, M(41, 8, 12x)) | 515,336 | 381,539 | 515,336 | 381,539 | 98.9 | 1.002 | 55 | 6 | 27 | 17.0 | 4.2 | 3,939 | 0 |
|  | **GAGE scaffolds** | GAGE-B(49) | 135,613 | 135,296 | 135,613 | 135,296 | 98.6 | 1.006 | 102 | 3 | 29 | 6.7 | 4.6 | 2,548 | 0 |
| **CABOG** | **Basic flow** | B(21, 100x) | 53,412 | 53,412 | 53,412 | 53,412 | 99.0 | 1.005 | 174 | 1 | 9 | 6.0 | 2.6 | 0 | 5,803 |
|  | **HGA Preprocessing** | AP(21, 2, 50x) | 38,529 | 38,529 | 37,388 | 37,388 | 98.8 | 1.005 | 228 | 1 | 10 | 4.9 | 2.6 | 1,071 | 5,752 |
|  |  | C(21, 4, 25x) | 52,734 | 52,734 | 52,733 | 52,733 | 98.5 | 1.001 | 196 | 0 | 12 | 6.2 | 2.4 | 2,266 | 5,787 |
|  | **HGA re-assembly Contigs** | HGA(91, C(21, 2, 50x)) | 254,859 | 242,515 | 254,859 | 242,515 | 98.9 | 1.002 | 67 | 6 | 21 | 11.7 | 2.3 | 3,864 | 5,924 |
|  |  | HGA(91, M(21, 8, 12x)) | 381,118 | 260,843 | 381,118 | 260,843 | 98.8 | 1.001 | 59 | 5 | 21 | 14.0 | 2.5 | 3,249 | 5,926 |
|  | **GAGE Contigs** | GAGE-B | 155,352 | 150,479 | 155,352 | 150,479 | 99.3 | 1.005 | 78 | 5 | 6 | 4.8 | 2.4 | 2,142 | 5,934 |
|  | **HGA re-assembly scaffolds** | HGA(91, C(21, 2, 50x)) | 319,561 | 269,969 | 319,561 | 269,969 | 98.9 | 1.002 | 63 | 6 | 23 | 12.1 | 2.4 | 3,864 | 0 |
|  |  | HGA(91, M(21, 8, 12x)) | 485,675 | 485,675 | 443,416 | 270,576 | 98.8 | **1.001** | 53 | 5 | 25 | 14.4 | 2.6 | 3,249 | 0 |
|  | **GAGE scaffolds** | GAGE-B | 431,479 | 364,209 | 431,479 | 364,209 | **99.4** | 1.005 | **33** | 9 | 13 | 4.4 | 3.0 | 2,142 | 0 |
| **MIRA** | **Basic flow** | B(21, 100x) | 5,205 | 5,198 | 10,381 | 10,328 | 99.2 | 2.079 | 4,618 | 31 | 41 | 9.9 | 3.2 | 19,388 | 5,675 |
|  | **HGA Preprocessing** | AP(21, 2, 50x) | 4,029 | 4,010 | 7,397 | 7,363 | 99.3 | 2.071 | 5,397 | 25 | 20 | 11.4 | 3.0 | 12,018 | 5,504 |
|  |  | C(21, 4, 25x) | 82,948 | 82,948 | 78,740 | 78,740 | 98.5 | 1.002 | 163 | 1 | 5 | 6.2 | 2.3 | 2,712 | 5,829 |
|  | **HGA re-assembly Contigs** | HGA(91, C(21, 1, 100x)) | 381,025 | 269,870 | 269,870 | 269,870 | 98.7 | 1.002 | 64 | 1 | 15 | 10.1 | 2.3 | 3,864 | 5,945 |
|  |  | HGA(91, M(21, 8, 12x)) | 330,615 | 260,692 | 330,615 | 260,692 | **99.4** | 1.002 | 63 | 13 | 27 | 12.8 | 3.3 | **1,380** | 5,943 |
|  | **GAGE Contigs** | GAGE-B | 116,480 | 100,038 | 116,480 | 100,038 | 99.2 | 1.007 | 153 | 9 | 14 | 4.8 | 2.1 | 5,903 | 5,919 |
|  | **HGA re-assembly scaffolds** | HGA(91, C(21, 1, 100x)) | 390,369 | 390,334 | 390,369 | 269,870 | 98.7 | 1.002 | 63 | 1 | 15 | 10.1 | 2.4 | 3,864 | 0 |
|  |  | HGA(91, M(21, 8, 12x)) | 330,615 | 260,692 | 330,615 | 260,692 | **99.4** | 1.002 | 60 | 13 | 29 | 13.0 | 3.1 | **1,380** | 0 |
|  | **GAGE scaffolds** | GAGE-B | - | - | - | - | - | - | - | - | - | - | - | - | - |
| **MaSuRCA** | **Basic flow** | B(61, 100x) | 80,722 | 80,722 | 80,722 | 80,722 | 98.7 | 1.007 | 146 | 2 | 7 | 14.6 | 2.4 | 2,574 | 5,880 |
|  | **HGA Preprocessing** | AP(51, 2, 50x) | 50,840 | 50,840 | 50,027 | 50,027 | 98.5 | 1.006 | 191 | 2 | 7 | 9.0 | 2.2 | 2,139 | 5,822 |
|  |  | C(91, 2, 50x) | 74,832 | 74,829 | 74,832 | 74,829 | 98.0 | 1.001 | 162 | 0 | 3 | 6.2 | 1.9 | 2,313 | 5,805 |
|  | **HGA re-assembly Contigs** | HGA(91, C(21, 1, 100x)) | 542,153 | 542,005 | 542,153 | **542,005** | 98.7 | 1.002 | 61 | 4 | 16 | 7.9 | 2.5 | 3,864 | 5,937 |
|  |  | HGA(81, M(21, 2, 50x)) | 485,217 | 485,217 | 485,217 | 485,217 | 98.8 | **1.001** | **50** | 2 | 18 | 9.4 | 3.1 | 3,708 | 5,946 |
|  | **GAGE Contigs** | GAGE-B(101) | 246,697 | 246,697 | 246,697 | 246,697 | 99.2 | 1.010 | 90 | 9 | 11 | 9.2 | 2.5 | 2,142 | **5,961** |
|  | **HGA re-assembly scaffolds** | HGA(91, C(21, 1, 100x)) | 542,153 | **542,005** | 542,153 | **542,005** | 98.7 | 1.002 | 60 | 4 | 17 | 8.0 | 2.4 | 3,864 | 0 |
|  |  | HGA(81, M(21, 2, 50x)) | 485,217 | 485,217 | 485,217 | 485,217 | 98.8 | **1.001** | 48 | 2 | 18 | 9.5 | 3.1 | 3,708 | 0 |
|  | **GAGE scaffolds** | GAGE-B(101) | 337,861 | 337,861 | 337,861 | 337,861 | 99.2 | 1.010 | 83 | 12 | 13 | 9.2 | 2.5 | 2,142 | 0 |
| **SGA** | **Basic flow** | B(81, 100x) | 22,358 | 22,358 | 26,552 | 26,552 | 99.0 | 1.157 | 3,529 | 18 | 8 | 2.1 | 2.0 | 5,592 | 5,754 |
|  | **HGA Preprocessing** | AP(51, 2, 50x) | 23,311 | 23,311 | 25,180 | 25,180 | 98.9 | 1.094 | 2,304 | 18 | 7 | 1.5 | 1.9 | 5,208 | 5,735 |
|  |  | C(61, 2, 50x) | 41,550 | 41,550 | 40,815 | 40,815 | 98.2 | 1.001 | 259 | 0 | 4 | 1.7 | 1.9 | 2,266 | 5,714 |
|  | **HGA re-assembly Contigs** | HGA(81, C(41, 1, 100x)) | 330,637 | 182,008 | 330,637 | 182,008 | 98.8 | 1.002 | 88 | 7 | 34 | 13.9 | 3.3 | 4,745 | 5,897 |
|  |  | HGA(91, M(41, 1, 100x)) | 209,067 | 208,896 | 209,067 | 208,896 | 98.7 | 1.002 | 83 | 2 | 28 | 10.6 | 2.4 | 3,517 | 5,916 |
|  | **GAGE Contigs** | GAGE-B(65) | 22,044 | 22,042 | 25,512 | 25,512 | 99.0 | 1.148 | 3,335 | 17 | 9 | **2.1** | **2.0** | 5,799 | 5,747 |
|  | **HGA re-assembly scaffolds** | HGA(81, C(41, 1, 100x)) | 331,201 | 208,794 | 331,201 | 208,794 | 98.8 | 1.002 | 78 | 7 | 41 | 13.7 | 3.4 | 4,745 | 0 |
|  |  | HGA(91, M(41, 1, 100x)) | 255,106 | 254,949 | 209,067 | 208,951 | 98.7 | 1.002 | 79 | 2 | 30 | 10.7 | 2.5 | 3,517 | 0 |
|  | **GAGE scaffolds** | GAGE-B(65) | 25,767 | 25,767 | 25,512 | 25,512 | 98.0 | 1.012 | 502 | 2 | **2** | **1.4** | **2.0** | 2,139 | 0 |
| **SOAPd2** | **Basic flow** | B(41, 100x) | 25,831 | 25,831 | 24,621 | 24,621 | 98.1 | 1.001 | 435 | 0 | 1 | 0.7 | 1.8 | 2,288 | 5,639 |
|  | **HGA Preprocessing** | AP(41, 2, 50x) | 14,299 | 14,299 | 13,798 | 13,798 | 98.1 | 1.002 | 769 | 0 | 2 | 0.9 | 1.9 | 2,366 | 5,388 |
|  |  | C(41, 2, 50x) | 28,369 | 28,369 | 27,308 | 27,308 | 97.9 | 1.000 | 387 | 0 | 2 | 1.5 | 1.9 | 2,266 | 5,657 |
|  | **HGA re-assembly Contigs** | HGA(81, C(21, 2, 50x)) | 452,656 | 229,509 | 452,656 | 229,509 | 98.7 | 1.002 | 72 | 4 | 26 | 13.4 | 3.3 | 3,744 | 5,916 |
|  |  | HGA(81, M(21, 8, 12x)) | 483,098 | 452,295 | 483,098 | 452,295 | 98.7 | 1.002 | 72 | 4 | 28 | 12.6 | 3.2 | 3,744 | 5,917 |
|  | **GAGE Contigs** | GAGE-B(55) | 246,346 | 246,346 | 246,346 | 246,346 | 98.4 | **1.001** | 105 | **0** | 20 | 9.0 | 2.7 | 2,316 | 5,834 |
|  | **HGA re-assembly scaffolds** | HGA(81, C(21, 2, 50x)) | 452,656 | 264,725 | 452,656 | 264,725 | 98.7 | 1.002 | 69 | 4 | 29 | 13.4 | 3.3 | 3,744 | 0 |
|  |  | HGA(81, M(21, 8, 12x)) | 483,098 | 452,295 | 483,098 | 452,295 | 98.8 | 1.002 | 68 | 6 | 30 | 12.7 | 3.2 | 3,744 | 0 |
|  | **GAGE scaffolds** | GAGE-B(55) | 456,635 | 455,989 | 456,635 | 455,989 | 98.4 | **1.001** | 77 | **0** | 39 | 8.1 | 2.9 | 2,316 | 0 |
| **SPAdes** | **Basic flow** | B(81, 100x) | 127,979 | 127,979 | 127,979 | 127,979 | 98.7 | 1.003 | 105 | 3 | 44 | 15.1 | 3.8 | 3,744 | 5,876 |
|  | **HGA Preprocessing** | AP(61, 2, 50x) | 90,025 | 89,938 | 89,201 | 89,111 | 98.6 | 1.003 | 164 | 6 | 31 | 16.3 | 3.4 | 3,799 | 5,831 |
|  |  | C(61, 4, 25x) | 168,952 | 168,950 | 168,952 | 168,950 | 98.4 | 1.006 | 115 | 2 | 20 | 7.4 | 3.0 | 5,279 | 5,842 |
|  | **HGA re-assembly Contigs** | HGA(81, C(21, 2, 50x)) | 754,905 | 283,161 | 754,905 | 283,161 | 98.8 | 1.002 | 60 | 5 | 22 | 14.5 | 3.5 | 3,744 | 5,924 |
|  |  | HGA(81, M(21, 8, 12x)) | 826,529 | 382,489 | 826,529 | 382,489 | 98.8 | 1.002 | 72 | 12 | 19 | 13.3 | 3.5 | 8,472 | 5,927 |
|  | **GAGE Contigs** | GAGE-B(51,63,85) | 103,691 | 103,691 | 103,691 | 103,691 | 99.1 | 1.377 | 49,967 | 8 | 14 | 19.0 | 3.0 | 10,464,230 | 5,911 |
|  | **HGA re-assembly scaffolds** | HGA(81, C(21, 2, 50x)) | 1,201,442 | 283,902 | 1,201,442 | 283,902 | 98.8 | 1.002 | 57 | 6 | 24 | 14.8 | 3.5 | 3,744 | 0 |
|  |  | HGA(81, M(21, 8, 12x)) | 931,487 | 444,453 | 931,487 | 444,453 | 98.8 | 1.002 | 68 | 12 | 21 | 13.7 | 3.6 | 227,499 | 0 |
|  | **GAGE scaffolds** | GAGE-B(51,63,85) | 212,506 | 212,506 | 212,506 | 212,506 | 99.1 | 1.377 | 49,919 | 9 | 37 | 18.3 | 3.4 | 10,463,127 | 0 |
| **Velvet** | **Basic flow** | B(51, 100x) | 30,953 | 30,899 | 30,655 | 30,519 | 98.2 | 1.002 | 345 | 8 | 20 | 13.3 | 3.2 | 2,506 | 5,672 |
|  | **HGA Preprocessing** | AP(41, 2, 50x) | 25,951 | 25,951 | 25,719 | 25,719 | 98.0 | 1.002 | 403 | 6 | 24 | 13.2 | 3.5 | 2,447 | 5,571 |
|  |  | C(31, 8, 12x) | 79,075 | 66,133 | 79,075 | 66,111 | 98.3 | 1.003 | 162 | 5 | 31 | 12.4 | 3.8 | 2,408 | 5,778 |
|  | **HGA re-assembly Contigs** | HGA(91, C(21, 8, 12x)) | 485,240 | 485,240 | 485,240 | 485,240 | 98.7 | 1.003 | 65 | 3 | 19 | 14.2 | 2.6 | 3,864 | 5,927 |
|  |  | HGA(81, M(21, 8, 12x)) | **1,276,342** | **1,276,279** | **1,276,342** | 485,259 | 98.8 | 1.002 | 58 | 7 | 24 | 11.9 | 3.5 | 3,744 | 5,933 |
|  | **GAGE Contigs** | GAGE-B(63) | 24,577 | 24,577 | 24,465 | 24,465 | 97.8 | **1.001** | 404 | 3 | 11 | 6.1 | 2.4 | 2,652 | 5,559 |
|  | **HGA re-assembly scaffolds** | HGA(91, C(21, 8, 12x)) | 485,240 | 485,240 | 485,240 | 485,240 | 98.7 | 1.003 | 63 | 3 | 20 | 14.5 | 2.6 | 3,864 | 0 |
|  |  | HGA(81, M(21, 8, 12x)) | **1,276,342** | 485,259 | **1,276,342** | 485,259 | 98.8 | 1.002 | 51 | 7 | 23 | 12.4 | 4.0 | 544,854 | 0 |
|  | **GAGE scaffolds** | GAGE-B(63) | 247,748 | 208,398 | 247,748 | 208,398 | 97.8 | 1.009 | 99 | 11 | 258 | 6.0 | 3.4 | 2,404 | 0 |

**Supplementary Table 4:** 251bp MiSeq read assemblies of *R. sphaeroides*, with reference genome size of 4,565,960bp and 4,474 genes. For metric and assembly flow descriptions see beginning of section.

| **Assembler** | **Type** | **Flow** | **N50** | **NA50** | **NG50** | **NGA50** | **Genome** | **Duplication** | **#** | **# mis-** | **Local mis-** | **# mismatches** | **# indels** | **Unaligned** | **#** |
| --- | --- | --- | --- | --- | --- | --- | --- | --- | --- | --- | --- | --- | --- | --- | --- |
|  |  |  |  |  |  |  | **fraction** | **ratio** | **contigs** | **assemblies** | **assemblies** | **per 100KB** | **per 100KB** | **length** | **Genes** |
| **ABySS** | **Basic flow** | B(41, 100x) | 112,782 | 112,782 | 115,786 | 114,140 | 99.6 | 1.021 | 120 | 12 | 25 | 17.4 | 4.1 | 1,178 | 4,383 |
|  | **HGA Preprocessing** | AP(31, 2, 50x) | 110,193 | 110,103 | 27,460 | 27,460 | 49.7 | 1.028 | 57 | 5 | 13 | 25.2 | 4.1 | 1,292 | 2,189 |
|  |  | C(31, 8, 12x) | 21,747 | 21,747 | 18,905 | 18,905 | 87.2 | 1.001 | 449 | 2 | 3 | 19.9 | 1.8 | 540 | 3,672 |
|  | **HGA re-assembly Contigs** | HGA(51, C(31, 1, 100x)) | 128,203 | 112,027 | 128,203 | 112,027 | 99.2 | 1.002 | 111 | 8 | 7 | 38.0 | 3.6 | 1,439 | 4,377 |
|  |  | HGA(31, M(31, 8, 12x)) | 173,288 | 170,032 | 173,288 | 170,032 | 99.0 | 1.003 | 109 | 8 | 12 | 36.7 | 2.6 | 3,329 | 4,386 |
|  | **GAGE Contigs** | GAGE-B(49) | 21,647 | 21,647 | 21,441 | 21,441 | 98.5 | 1.001 | 486 | 1 | 2 | 3.5 | 0.2 | 96 | 4,121 |
|  | **HGA re-assembly scaffolds** | HGA(51, C(31, 1, 100x)) | 128,203 | 112,027 | 128,203 | 112,027 | 99.2 | 1.002 | 108 | 8 | 7 | 38.3 | 3.6 | 1,439 | 0 |
|  |  | HGA(31, M(31, 8, 12x)) | 174,187 | 173,288 | 174,187 | 173,288 | 99.0 | 1.003 | 104 | 8 | 16 | 37.9 | 2.7 | 3,329 | 0 |
|  | **GAGE scaffolds** | GAGE-B(49) | 21,647 | 21,647 | 21,441 | 21,441 | 98.5 | **1.001** | 486 | 1 | 2 | 3.5 | **0.2** | 96 | 0 |
| **CABOG** | **Basic flow** | B(21, 100x) | 19,766 | 19,554 | 9,985 | 9,903 | 59.2 | 1.000 | 185 | 3 | 1 | 9.0 | 0.4 | 0 | 2,496 |
|  | **HGA Preprocessing** | AP(21, 2, 50x) | 20,414 | 20,414 | 0 | 0 | 31.9 | 1.001 | 104 | 1 | 1 | 10.8 | 0.6 | 16 | 1,342 |
|  |  | C(21, 2, 50x) | 16,115 | 16,115 | 0 | 0 | 28.7 | 1.001 | 195 | 4 | 0 | 6.8 | 0.2 | 0 | 1,208 |
|  | **HGA re-assembly Contigs** | HGA(51, C(21, 8, 12x)) | 127,081 | 127,081 | 127,081 | 127,081 | 99.3 | 1.002 | 138 | 9 | 5 | 22.7 | 2.2 | 1,578 | 4,368 |
|  |  | HGA(31, M(21, 4, 25x)) | 167,899 | 136,193 | 167,899 | 136,193 | 99.4 | 1.002 | 129 | 10 | 10 | 40.1 | 2.8 | 2,478 | 4,390 |
|  | **GAGE Contigs** | GAGE-B | 41,794 | 41,176 | 31,540 | 30,364 | 85.7 | 1.004 | 146 | 6 | 2 | 7.1 | 0.5 | 78 | 3,738 |
|  | **HGA re-assembly scaffolds** | HGA(51, C(21, 8, 12x)) | 127,081 | 127,081 | 127,081 | 127,081 | 99.3 | 1.002 | 131 | 10 | 8 | 22.7 | 2.3 | 1,578 | 0 |
|  |  | HGA(31, M(21, 4, 25x)) | 173,888 | 147,716 | 173,888 | 147,716 | 99.4 | 1.003 | 124 | 10 | 13 | 40.8 | 2.8 | 2,478 | 0 |
|  | **GAGE scaffolds** | GAGE-B | 45,887 | 42,489 | 40,287 | 36,097 | 85.7 | 1.004 | 131 | 6 | 6 | 7.2 | 1.0 | 78 | 0 |
| **MIRA** | **Basic flow** | B(101, 100x) | 0 | 0 | 0 | 0 | 0.0 | 0.000 | 0 | 0 | 0 | 0.0 | 0.0 | 0 | 0 |
|  | **HGA Preprocessing** | AP(101, 8, 12x) | 0 | 0 | 0 | 0 | 0.0 | 0.000 | 0 | 0 | 0 | 0.0 | 0.0 | 0 | 0 |
|  |  | C(101, 8, 12x) | 0 | 0 | 0 | 0 | 0.0 | 0.000 | 0 | 0 | 0 | 0.0 | 0.0 | 0 | 0 |
|  | **HGA re-assembly Contigs** | HGA(41, C(21, 8, 12x)) | 124,257 | 124,225 | 124,257 | 124,225 | 99.1 | **1.001** | 150 | 3 | 5 | 18.5 | 1.9 | 1,766 | 4,348 |
|  |  | HGA(41, M(21, 8, 12x)) | 124,257 | 124,225 | 124,257 | 124,225 | 99.1 | **1.001** | 150 | 3 | 5 | 18.5 | 1.9 | 1,766 | 4,348 |
|  | **GAGE Contigs** | GAGE-B | 15,445 | 15,271 | 15,792 | 15,445 | 99.3 | 1.022 | 867 | 16 | 4 | 39.2 | 2.8 | 43 | 3,951 |
|  | **HGA re-assembly scaffolds** | HGA(41, C(21, 8, 12x)) | 130,354 | 130,354 | 127,712 | 127,712 | 99.2 | **1.001** | 142 | 3 | 9 | 19.5 | 2.0 | 1,766 | 0 |
|  |  | HGA(41, M(21, 8, 12x)) | 130,354 | 130,354 | 127,712 | 127,712 | 99.2 | **1.001** | 142 | 3 | 9 | 19.5 | 2.0 | 1,766 | 0 |
|  | **GAGE scaffolds** | GAGE-B | - | - | - | - | - | - | - | - | - | - | - | - | - |
| **MaSuRCA** | **Basic flow** | B(41, 100x) | 116,186 | 116,186 | 112,886 | 112,886 | 93.8 | 1.004 | 88 | 3 | 2 | 3.4 | 0.7 | 2,450 | 4,175 |
|  | **HGA Preprocessing** | AP(41, 2, 50x) | 114,614 | 114,614 | 24,633 | 24,633 | 47.0 | 1.004 | 41 | 2 | 1 | 4.3 | 0.8 | 1,225 | 2,091 |
|  |  | C(21, 4, 25x) | 26,970 | 26,970 | 21,651 | 21,400 | 81.3 | 1.001 | 365 | 1 | 6 | 5.1 | 0.4 | 26 | 3,497 |
|  | **HGA re-assembly Contigs** | HGA(71, C(51, 8, 12x)) | 178,403 | 144,067 | 170,376 | 144,067 | 99.3 | 1.003 | 91 | 11 | 3 | 22.7 | 1.0 | 356 | 4,397 |
|  |  | HGA(101, M(21, 4, 25x)) | **251,279** | 212,893 | **251,279** | 212,893 | **99.8** | **1.001** | **52** | 7 | 7 | 9.9 | 0.7 | **0** | **4,430** |
|  | **GAGE Contigs** | GAGE-B(63) | 142,742 | 142,742 | 130,714 | 130,714 | 92.1 | 1.004 | 63 | 5 | 3 | 9.8 | 0.8 | 341 | 4,109 |
|  | **HGA re-assembly scaffolds** | HGA(71, C(51, 8, 12x)) | 178,403 | 144,067 | 170,376 | 144,067 | 99.3 | 1.003 | 89 | 12 | 4 | 23.0 | 1.1 | 356 | 0 |
|  |  | HGA(101, M(21, 4, 25x)) | **251,279** | 212,893 | **251,279** | 212,893 | **99.8** | **1.001** | **52** | 7 | 7 | 9.9 | 0.7 | **0** | 0 |
|  | **GAGE scaffolds** | GAGE-B(63) | 165,131 | 165,131 | 144,812 | 144,812 | 92.0 | 1.004 | 55 | 5 | 6 | 9.7 | 0.9 | 341 | 0 |
| **SGA** | **Basic flow** | B(51, 100x) | 19,331 | 19,229 | 19,703 | 19,229 | 99.1 | 1.016 | 785 | 4 | 2 | 1.0 | 0.2 | 1,204 | 4,107 |
|  | **HGA Preprocessing** | AP(51, 8, 12x) | 21,546 | 21,545 | 0 | 0 | 12.4 | 1.015 | 74 | 1 | 0 | 2.3 | 0.7 | 0 | 518 |
|  |  | C(51, 1, 100x) | 14,751 | 14,460 | 1,967 | 1,903 | 52.7 | 1.002 | 426 | 7 | 0 | 9.8 | 0.5 | 129 | 2,168 |
|  | **HGA re-assembly Contigs** | HGA(41, C(61, 8, 12x)) | 144,749 | 126,907 | 144,749 | 126,907 | 99.2 | 1.003 | 138 | 9 | 5 | 27.6 | 2.6 | 1,766 | 4,365 |
|  |  | HGA(31, M(101, 8, 12x)) | 170,071 | 170,071 | 170,071 | 170,071 | 99.1 | 1.002 | 107 | 4 | 6 | 38.6 | 2.0 | 2,672 | 4,384 |
|  | **GAGE Contigs** | GAGE-B(23) | 9,086 | 9,086 | 9,108 | 9,108 | 99.0 | 1.013 | 986 | 4 | **2** | **0.8** | 0.4 | 763 | 3,807 |
|  | **HGA re-assembly scaffolds** | HGA(41, C(61, 8, 12x)) | 147,992 | 144,746 | 147,992 | 144,746 | 99.2 | 1.003 | 130 | 9 | 9 | 27.8 | 2.7 | 1,766 | 0 |
|  |  | HGA(31, M(101, 8, 12x)) | 170,071 | 170,071 | 170,071 | 170,071 | 99.1 | 1.002 | 104 | 4 | 8 | 38.6 | 2.0 | 2,672 | 0 |
|  | **GAGE scaffolds** | GAGE-B(23) | 9,055 | 9,055 | 7,971 | 7,971 | 88.6 | 1.009 | 778 | **0** | **1** | **0.3** | 0.4 | 266 | 0 |
| **SOAPd2** | **Basic flow** | B(41, 100x) | 24,166 | 23,927 | 23,927 | 23,780 | 98.9 | 1.001 | 460 | 1 | 1 | 0.5 | 0.2 | 0 | 4,161 |
|  | **HGA Preprocessing** | AP(41, 8, 12x) | 24,787 | 24,787 | 0 | 0 | 12.5 | 1.002 | 49 | 0 | 0 | 0.5 | 0.2 | 5 | 527 |
|  |  | C(31, 1, 100x) | 16,391 | 16,391 | 11,001 | 11,001 | 75.7 | 1.002 | 510 | 1 | 1 | 5.3 | 0.2 | 0 | 3,132 |
|  | **HGA re-assembly Contigs** | HGA(41, C(21, 8, 12x)) | 174,187 | 157,067 | 174,187 | 157,067 | 99.2 | **1.001** | 127 | 7 | 5 | 26.8 | 2.9 | 4,011 | 4,367 |
|  |  | HGA(61, M(31, 4, 25x)) | 176,506 | 162,346 | 176,506 | 162,346 | 99.5 | 1.002 | 95 | 6 | 3 | 14.8 | 1.1 | 376 | 4,405 |
|  | **GAGE Contigs** | GAGE-B(79) | 33,829 | 33,829 | 33,491 | 33,491 | 98.4 | 1.005 | 437 | **1** | 10 | 3.6 | 0.4 | 5,085 | 4,134 |
|  | **HGA re-assembly scaffolds** | HGA(41, C(21, 8, 12x)) | 193,157 | 193,157 | 180,964 | 177,461 | 99.2 | **1.001** | 119 | 7 | 8 | 27.9 | 3.1 | 4,011 | 0 |
|  |  | HGA(61, M(31, 4, 25x)) | 177,971 | 175,390 | 177,971 | 175,390 | 99.5 | 1.002 | 91 | 6 | 3 | 14.7 | 1.1 | 376 | 0 |
|  | **GAGE scaffolds** | GAGE-B(79) | 45,133 | 45,022 | 45,133 | 45,022 | 98.4 | 1.007 | 226 | 4 | 213 | 3.5 | 0.4 | 4,702 | 0 |
| **SPAdes** | **Basic flow** | B(41, 100x) | 124,257 | 124,225 | 124,257 | 124,225 | 99.1 | 1.001 | 151 | 3 | 5 | 18.4 | 1.9 | 1,766 | 4,348 |
|  | **HGA Preprocessing** | AP(21, 8, 12x) | 211,854 | 211,852 | 0 | 0 | 12.4 | 1.001 | 21 | 3 | 2 | 27.7 | 1.7 | 856 | 547 |
|  |  | C(51, 8, 12x) | 26,349 | 26,349 | 22,593 | 22,593 | 88.6 | 1.001 | 414 | 3 | 2 | 4.0 | 0.6 | 0 | 3,771 |
|  | **HGA re-assembly Contigs** | HGA(51, C(21, 4, 25x)) | 196,881 | 174,187 | 196,881 | 174,187 | 99.5 | 1.004 | 113 | 5 | 4 | 27.4 | 1.8 | 1,578 | 4,402 |
|  |  | HGA(31, M(21, 4, 25x)) | 244,543 | **244,543** | 244,543 | **244,543** | 98.9 | 1.004 | 130 | 6 | 13 | 38.8 | 2.1 | 5,771 | 4,380 |
|  | **GAGE Contigs** | GAGE-B(31,43,65) | 118,093 | 115,059 | 118,093 | 118,093 | 99.6 | 1.004 | 185 | 5 | 4 | 8.2 | 0.8 | 13,389 | 4,384 |
|  | **HGA re-assembly scaffolds** | HGA(51, C(21, 4, 25x)) | 204,704 | 188,727 | 204,704 | 188,727 | 99.5 | 1.004 | 110 | 6 | 4 | 27.3 | 1.8 | 1,578 | 0 |
|  |  | HGA(31, M(21, 4, 25x)) | 244,543 | **244,543** | 244,543 | **244,543** | 98.9 | 1.004 | 127 | 6 | 14 | 39.6 | 2.1 | 5,771 | 0 |
|  | **GAGE scaffolds** | GAGE-B(31,43,65) | 151,794 | 151,794 | 151,794 | 151,794 | 99.7 | 1.016 | 145 | 8 | 8 | 7.6 | 1.9 | 13,389 | 0 |
| **Velvet** | **Basic flow** | B(41, 100x) | 29,448 | 28,750 | 29,092 | 28,677 | 98.8 | 1.003 | 348 | 5 | 11 | 16.2 | 1.2 | 0 | 4,213 |
|  | **HGA Preprocessing** | AP(31, 8, 12x) | 35,181 | 33,678 | 0 | 0 | 12.4 | 1.001 | 32 | 1 | 2 | 17.6 | 1.2 | 481 | 534 |
|  |  | C(21, 8, 12x) | 17,228 | 16,762 | 2,996 | 2,864 | 52.8 | 0.999 | 325 | 8 | 6 | 12.9 | 1.2 | 1,129 | 2,239 |
|  | **HGA re-assembly Contigs** | HGA(41, C(21, 8, 12x)) | 170,405 | 157,915 | 170,405 | 147,761 | 99.4 | **1.001** | 127 | 9 | 7 | 25.6 | 2.5 | 1,766 | 4,375 |
|  |  | HGA(31, M(21, 8, 12x)) | 204,678 | 204,678 | 204,678 | 204,678 | 99.0 | 1.003 | 100 | 8 | 14 | 36.8 | 2.5 | 2,821 | 4,388 |
|  | **GAGE Contigs** | GAGE-B(31) | 24,300 | 24,248 | 24,248 | 24,205 | 98.0 | **1.001** | 416 | 2 | 8 | 11.6 | 0.6 | 688 | 4,136 |
|  | **HGA re-assembly scaffolds** | HGA(41, C(21, 8, 12x)) | 176,125 | 170,405 | 174,871 | 170,405 | 99.4 | **1.001** | 120 | 9 | 10 | 26.6 | 2.7 | 1,766 | 0 |
|  |  | HGA(31, M(21, 8, 12x)) | 204,678 | 204,678 | 204,678 | 204,678 | 99.0 | 1.003 | 97 | 8 | 15 | 37.1 | 2.6 | 2,821 | 0 |
|  | **GAGE scaffolds** | GAGE-B(31) | 88,399 | 84,960 | 85,272 | 84,960 | 98.0 | 1.009 | 209 | 19 | 184 | 11.5 | 1.1 | **0** | 0 |

**Supplementary Table 5:** 101bp HiSeq read assemblies of *R. sphaeroides*, with reference genome size of 4,565,960bp and 4,474 genes. For metric and assembly flow descriptions see beginning of section.

| **Assembler** | **Type** | **Flow** | **N50** | **NA50** | **NG50** | **NGA50** | **Genome** | **Duplication** | **#** | **# mis-** | **Local mis-** | **# mismatches** | **# indels** | **Unaligned** | **#** |
| --- | --- | --- | --- | --- | --- | --- | --- | --- | --- | --- | --- | --- | --- | --- | --- |
|  |  |  |  |  |  |  | **fraction** | **ratio** | **contigs** | **assemblies** | **assemblies** | **per 100KB** | **per 100KB** | **length** | **Genes** |
| **ABySS** | **Basic flow** | B(31, 210x) | 120,554 | 120,554 | 120,554 | 120,554 | 99.0 | 1.019 | 145 | 9 | 10 | 15.6 | 5.6 | 0 | 4,352 |
|  | **HGA Preprocessing** | AP(31, 2, 105x) | 98,263 | 98,263 | 98,795 | 98,263 | 98.9 | 1.019 | 174 | 9 | 7 | 16.4 | 9.5 | 58 | 4,323 |
|  |  | C(31, 4, 52x) | 51,404 | 51,404 | 49,231 | 49,231 | 98.1 | 1.007 | 277 | 2 | 29 | 206.7 | 17.0 | 213 | 4,204 |
|  | **HGA re-assembly Contigs** | HGA(41, C(21, 2, 105x)) | 161,703 | 136,021 | 161,703 | 136,021 | 99.0 | 1.000 | 202 | 7 | 12 | 29.9 | 2.7 | 8,396 | 4,357 |
|  |  | HGA(31, M(21, 4, 52x)) | 187,637 | 187,637 | 187,637 | 187,637 | 98.6 | 1.002 | 236 | 10 | 15 | 34.2 | 2.5 | 15,503 | 4,338 |
|  | **GAGE Contigs** | GAGE-B(31) | 13,460 | 13,460 | 13,319 | 13,319 | 98.4 | 1.009 | 604 | 6 | 2 | 8.8 | 0.7 | 661 | 4,030 |
|  | **HGA re-assembly scaffolds** | HGA(41, C(21, 2, 105x)) | 173,221 | 161,703 | 173,221 | 161,703 | 99.1 | 1.001 | 196 | 7 | 16 | 34.1 | 2.9 | 8,396 | 0 |
|  |  | HGA(31, M(21, 4, 52x)) | 229,984 | 229,984 | 229,984 | 229,984 | 98.6 | 1.002 | 230 | 10 | 16 | 34.1 | 2.7 | 15,503 | 0 |
|  | **GAGE scaffolds** | GAGE-B(31) | 13,475 | 13,475 | 13,460 | 13,460 | 98.5 | 1.010 | 582 | 34 | 11 | 9.1 | 0.7 | **27** | 0 |
| **CABOG** | **Basic flow** | B(21, 210x) | 4,462 | 4,462 | 3,232 | 3,232 | 75.7 | 1.001 | 1,001 | 2 | 2 | 15.9 | 1.3 | 0 | 2,606 |
|  | **HGA Preprocessing** | AP(21, 2, 105x) | 2,949 | 2,947 | 1,507 | 1,507 | 57.2 | 1.004 | 1,018 | 3 | 2 | 14.5 | 1.2 | 69 | 1,821 |
|  |  | C(21, 1, 210x) | 4,861 | 4,256 | 0 | 0 | 11.8 | 1.000 | 225 | 12 | 0 | 70.6 | 7.3 | 288 | 410 |
|  | **HGA re-assembly Contigs** | HGA(31, C(21, 8, 26x)) | 127,178 | 112,298 | 127,178 | 112,298 | 98.7 | 1.001 | 259 | 11 | 19 | 24.3 | 2.1 | 15,503 | 4,321 |
|  |  | HGA(31, M(21, 1, 210x)) | 143,313 | 127,178 | 143,313 | 127,178 | 99.0 | 1.002 | 204 | 8 | 18 | 33.6 | 2.2 | 15,897 | 4,362 |
|  | **GAGE Contigs** | GAGE-B | 12,421 | 12,360 | 11,358 | 11,217 | 90.9 | 1.008 | 537 | 4 | 3 | 20.1 | 1.6 | 360 | 3,619 |
|  | **HGA re-assembly scaffolds** | HGA(31, C(21, 8, 26x)) | 186,283 | 161,627 | 186,283 | 161,627 | 98.7 | 1.001 | 245 | 11 | 29 | 24.2 | 2.1 | 15,503 | 0 |
|  |  | HGA(31, M(21, 1, 210x)) | 201,144 | 201,144 | 201,144 | 201,144 | 99.0 | 1.002 | 191 | 8 | 28 | 33.6 | 2.2 | 15,897 | 0 |
|  | **GAGE scaffolds** | GAGE-B | 23,585 | 23,406 | 21,196 | 20,580 | 90.9 | 1.009 | 320 | 6 | 33 | 21.7 | 6.7 | 146 | 0 |
| **MIRA** | **Basic flow** | B(91, 210x) | - | - | - | - | - | - | - | - | - | - | - | - | - |
|  | **HGA Preprocessing** | AP(91, 8, 26x) | - | - | - | - | - | - | - | - | - | - | - | - | - |
|  |  | C(91, 8, 26x) | - | - | - | - | - | - | - | - | - | - | - | - | - |
|  | **HGA re-assembly Contigs** | HGA(31, C(21, 8, 26x)) | - | - | - | - | - | - | - | - | - | - | - | - | - |
|  |  | HGA(31, M(21, 8, 26x)) | - | - | - | - | - | - | - | - | - | - | - | - | - |
|  | **GAGE Contigs** | GAGE-B | - | - | - | - | - | - | - | - | - | - | - | - | - |
|  | **HGA re-assembly scaffolds** | HGA(31, C(21, 8, 26x)) | - | - | - | - | - | - | - | - | - | - | - | - | - |
|  |  | HGA(31, M(21, 8, 26x)) | - | - | - | - | - | - | - | - | - | - | - | - | - |
|  | **GAGE scaffolds** | GAGE-B | - | - | - | - | - | - | - | - | - | - | - | - | - |
| **MaSuRCA** | **Basic flow** | B(41, 210x) | 24,800 | 24,800 | 24,406 | 24,406 | 95.6 | 1.020 | 382 | 2 | 2 | 47.5 | 0.5 | 219 | 4,093 |
|  | **HGA Preprocessing** | AP(41, 2, 105x) | 41,079 | 40,951 | 39,503 | 39,307 | 95.8 | 1.009 | 226 | 3 | 4 | 22.8 | 0.4 | 25 | 4,177 |
|  |  | C(51, 2, 105x) | 103,409 | 103,409 | 102,493 | 102,493 | 95.2 | 1.002 | 154 | 0 | 2 | 18.3 | 0.4 | 0 | 4,208 |
|  | **HGA re-assembly Contigs** | HGA(51, C(21, 2, 105x)) | 284,717 | 222,832 | 284,717 | 222,832 | 99.1 | 1.002 | **127** | 5 | 10 | 16.6 | 1.5 | 4,627 | **4,381** |
|  |  | HGA(41, M(21, 1, 210x)) | 209,442 | 168,043 | 209,442 | 168,043 | 99.2 | 1.001 | 159 | 9 | 17 | 21.9 | 1.5 | 7,732 | 4,375 |
|  | **GAGE Contigs** | GAGE-B(55) | 176,783 | 176,783 | 176,783 | 176,783 | 97.1 | 1.005 | 130 | 5 | 4 | 72.1 | 1.0 | **49** | 4,298 |
|  | **HGA re-assembly scaffolds** | HGA(51, C(21, 2, 105x)) | 284,717 | 222,832 | 284,717 | 222,832 | 99.1 | 1.002 | 126 | 5 | 10 | 16.6 | 1.5 | 4,627 | 0 |
|  |  | HGA(41, M(21, 1, 210x)) | 209,442 | 185,588 | 209,442 | 185,588 | 99.2 | 1.002 | 155 | 10 | 19 | 26.0 | 2.0 | 7,732 | 0 |
|  | **GAGE scaffolds** | GAGE-B(55) | 196,511 | 196,511 | 196,511 | 196,511 | 97.1 | 1.005 | **125** | 6 | 6 | 72.1 | 1.1 | 49 | 0 |
| **SGA** | **Basic flow** | B(41, 210x) | 12,788 | 12,788 | 12,113 | 12,113 | 93.0 | 1.003 | 845 | 1 | 1 | 2.2 | 0.2 | 49 | 3,687 |
|  | **HGA Preprocessing** | AP(41, 2, 105x) | 10,121 | 10,111 | 9,165 | 9,134 | 89.8 | 1.004 | 902 | 2 | 1 | 2.1 | 0.2 | 159 | 3,479 |
|  |  | C(21, 2, 105x) | 23,702 | 22,341 | 21,543 | 20,662 | 94.7 | 1.000 | 444 | 3 | 5 | 3.7 | 0.3 | 0 | 3,940 |
|  | **HGA re-assembly Contigs** | HGA(31, C(51, 8, 26x)) | 148,826 | 127,185 | 148,826 | 127,185 | 98.8 | 1.001 | 236 | 4 | 21 | 27.0 | 2.2 | 15,291 | 4,337 |
|  |  | HGA(31, M(21, 1, 210x)) | 159,189 | 146,650 | 159,189 | 146,650 | 98.5 | 1.002 | 234 | 13 | 18 | 28.9 | 1.8 | 15,773 | 4,322 |
|  | **GAGE Contigs** | GAGE-B(41) | 12,793 | 12,793 | 12,057 | 12,057 | 92.9 | 1.004 | 862 | **1** | **1** | **2.1** | **0.2** | 205 | 3,679 |
|  | **HGA re-assembly scaffolds** | HGA(31, C(51, 8, 26x)) | 185,025 | 185,025 | 185,025 | 185,025 | 98.8 | 1.001 | 222 | 4 | 31 | 27.3 | 2.3 | 15,291 | 0 |
|  |  | HGA(31, M(21, 1, 210x)) | 159,189 | 146,650 | 159,189 | 146,650 | 98.5 | 1.002 | 231 | 13 | 21 | 28.6 | 1.8 | 15,773 | 0 |
|  | **GAGE scaffolds** | GAGE-B(41) | 13,487 | 13,487 | 11,833 | 11,793 | 87.2 | 1.003 | 662 | **1** | **1** | **1.7** | **0.2** | 54 | 0 |
| **SOAPd2** | **Basic flow** | B(61, 210x) | 3,066 | 3,062 | 3,014 | 3,008 | 97.1 | 1.009 | 2,476 | 1 | 1 | 34.2 | 1.0 | 2,299 | 2,796 |
|  | **HGA Preprocessing** | AP(51, 2, 105x) | 4,239 | 4,231 | 4,185 | 4,165 | 98.0 | 1.008 | 1,986 | 1 | 2 | 25.9 | 0.8 | 1,737 | 3,101 |
|  |  | C(41, 4, 52x) | 20,999 | 20,712 | 20,712 | 20,516 | 98.4 | 1.002 | 498 | 2 | 1 | 9.4 | 0.5 | 215 | 4,092 |
|  | **HGA re-assembly Contigs** | HGA(31, C(41, 1, 210x)) | 157,339 | 147,243 | 157,339 | 147,243 | 98.6 | 1.002 | 237 | 5 | 21 | 23.3 | 1.8 | 15,540 | 4,330 |
|  |  | HGA(31, M(41, 2, 105x)) | 126,438 | 126,438 | 126,438 | 126,438 | 98.6 | 1.002 | 257 | 5 | 38 | 23.9 | 2.7 | 15,664 | 4,306 |
|  | **GAGE Contigs** | GAGE-B(55) | 10,993 | 10,906 | 10,600 | 10,512 | 97.3 | 1.002 | 859 | 2 | 9 | 25.6 | 1.9 | 1,125 | 3,752 |
|  | **HGA re-assembly scaffolds** | HGA(31, C(41, 1, 210x)) | 185,556 | 185,548 | 185,556 | 185,548 | 98.6 | 1.002 | 228 | 5 | 26 | 23.0 | 2.0 | 15,540 | 0 |
|  |  | HGA(31, M(41, 2, 105x)) | 161,484 | 161,478 | 161,484 | 161,478 | 98.6 | 1.002 | 243 | 5 | 44 | 23.1 | 2.8 | 15,641 | 0 |
|  | **GAGE scaffolds** | GAGE-B(55) | 15,850 | 15,839 | 15,520 | 15,517 | 97.2 | 1.003 | 661 | 2 | 126 | 24.6 | 4.3 | 6,512 | 0 |
| **SPAdes** | **Basic flow** | B(31, 210x) | 112,631 | 112,631 | 112,301 | 112,298 | 98.5 | 1.002 | 281 | 1 | 21 | 18.1 | 1.6 | 15,503 | 4,310 |
|  | **HGA Preprocessing** | AP(31, 2, 105x) | 64,334 | 64,334 | 63,825 | 63,825 | 98.5 | 1.003 | 294 | 4 | 27 | 18.2 | 2.5 | 4,845 | 4,263 |
|  |  | C(31, 4, 52x) | 111,793 | 111,793 | 111,793 | 105,389 | 98.4 | 1.007 | 219 | 9 | 29 | 12.3 | 3.1 | 198 | 4,294 |
|  | **HGA re-assembly Contigs** | HGA(41, C(21, 2, 105x)) | 286,024 | 286,024 | 286,024 | 286,024 | 99.0 | **1.000** | 169 | 8 | 19 | 27.8 | 1.8 | 8,099 | 4,376 |
|  |  | HGA(41, M(21, 1, 210x)) | **314,739** | **314,739** | **314,739** | **314,739** | 99.0 | 1.001 | 269 | 7 | 16 | 31.3 | 2.2 | 30,304 | 4,363 |
|  | **GAGE Contigs** | GAGE-B(21,33,55) | 74,486 | 74,016 | 83,463 | 83,460 | **99.5** | 1.014 | 298 | 6 | 5 | 6.0 | 0.8 | 25,816 | 4,350 |
|  | **HGA re-assembly scaffolds** | HGA(41, C(21, 2, 105x)) | 331,799 | 331,799 | 331,799 | 331,799 | 99.0 | **1.000** | 163 | 9 | 21 | 28.1 | 2.0 | 8,099 | 0 |
|  |  | HGA(41, M(21, 1, 210x)) | **465,114** | **465,114** | **465,114** | **465,114** | 99.0 | 1.001 | 265 | 8 | 18 | 34.4 | 2.5 | 30,304 | 0 |
|  | **GAGE scaffolds** | GAGE-B(21,33,55) | 127,911 | 127,911 | 127,911 | 127,911 | **99.5** | 1.013 | 259 | 6 | 9 | 6.3 | 1.4 | 25,919 | 0 |
| **Velvet** | **Basic flow** | B(51, 210x) | 12,779 | 12,779 | 12,225 | 12,225 | 98.0 | 1.003 | 729 | 2 | 5 | 14.9 | 1.2 | 552 | 3,864 |
|  | **HGA Preprocessing** | AP(41, 2, 105x) | 14,403 | 14,178 | 14,045 | 13,979 | 98.2 | 1.002 | 638 | 3 | 5 | 17.3 | 0.8 | 384 | 3,949 |
|  |  | C(31, 4, 52x) | 44,626 | 44,626 | 44,315 | 44,315 | 98.4 | 1.001 | 338 | 1 | 6 | 12.0 | 0.7 | 21 | 4,222 |
|  | **HGA re-assembly Contigs** | HGA(31, C(31, 8, 26x)) | 148,202 | 130,168 | 148,202 | 130,168 | 98.7 | 1.008 | 216 | 14 | 22 | 28.2 | 2.1 | 15,291 | 4,340 |
|  |  | HGA(31, M(41, 1, 210x)) | 159,736 | 150,263 | 150,266 | 150,263 | 98.6 | 1.002 | 220 | 9 | 21 | 24.0 | 2.3 | 15,291 | 4,334 |
|  | **GAGE Contigs** | GAGE-B(49) | 13,800 | 13,775 | 13,087 | 13,087 | 97.9 | 1.001 | 696 | 2 | 4 | 10.2 | 0.3 | 404 | 3,904 |
|  | **HGA re-assembly scaffolds** | HGA(31, C(31, 4, 52x)) | 190,800 | 190,772 | 190,800 | 190,772 | 98.6 | 1.003 | 213 | 7 | 22 | 26.0 | 1.8 | 15,305 | 0 |
|  |  | HGA(31, M(51, 1, 210x)) | 185,472 | 185,440 | 185,472 | 185,440 | 98.7 | 1.001 | 208 | 4 | 36 | 26.5 | 3.3 | 15,291 | 0 |
|  | **GAGE scaffolds** | GAGE-B(49) | 36,086 | 33,823 | 34,182 | 33,086 | 98.0 | 1.010 | 348 | 20 | 333 | 10.4 | 0.7 | 252 | 0 |

**Supplementary Table 6:** 250bp MiSeq read assemblies of *M. abscessus*, with reference genome size of 5,090,401bp and 4,992 genes. For metric and assembly flow descriptions see beginning of section.

| **Assembler** | **Type** | **Flow** | **N50** | **NA50** | **NG50** | **NGA50** | **Genome** | **Duplication** | **#** | **# mis-** | **Local mis-** | **# mismatches** | **# indels** | **Unaligned** | **#** |
| --- | --- | --- | --- | --- | --- | --- | --- | --- | --- | --- | --- | --- | --- | --- | --- |
|  |  |  |  |  |  |  | **fraction** | **ratio** | **contigs** | **assemblies** | **assemblies** | **per 100KB** | **per 100KB** | **length** | **Genes** |
| **ABySS** | **Basic flow** | B(51, 100x) | 87,472 | 86,675 | 87,624 | 86,675 | 100.6 | 1.001 | 168 | 2 | 5 | 4.1 | 1.1 | 80,087 | 4,853 |
|  | **HGA Preprocessing** | AP(41, 2, 50x) | 40,153 | 37,883 | 40,153 | 38,366 | 100.4 | 1.001 | 322 | 4 | 2 | 3.1 | 1.0 | 79,271 | 4,739 |
|  |  | C(61, 4, 25x) | 79,635 | 75,731 | 79,635 | 75,731 | 100.4 | 1.002 | 189 | 5 | 3 | 10.0 | 1.1 | 76,474 | 4,831 |
|  | **HGA re-assembly Contigs** | HGA(31, C(51, 4, 25x)) | 272,898 | 205,053 | 272,898 | 205,053 | 100.6 | 1.003 | 496 | 6 | 8 | 12.8 | 0.8 | 222,852 | 4,902 |
|  |  | HGA(41, M(91, 8, 12x)) | 294,301 | 199,090 | 294,301 | 225,649 | 100.7 | 1.016 | 408 | 10 | 6 | 10.5 | 1.3 | 136,348 | 4,915 |
|  | **GAGE Contigs** | GAGE-B(58) | 70,424 | 67,488 | 70,424 | 68,549 | 99.2 | 1.001 | 210 | 2 | 2 | 1.9 | 0.6 | 80,486 | 4,825 |
|  | **HGA re-assembly scaffolds** | HGA(31, C(51, 4, 25x)) | 272,898 | 210,070 | 274,707 | 225,649 | 100.6 | 1.051 | 492 | 10 | 9 | 15.9 | 1.1 | 168,639 | 0 |
|  |  | HGA(41, M(91, 8, 12x)) | 340,251 | 225,649 | 388,189 | 231,650 | 100.7 | 1.016 | 406 | 11 | 6 | 12.5 | 1.5 | 662,877 | 0 |
|  | **GAGE scaffolds** | GAGE-B(58) | 73,179 | 73,179 | 70,126 | 70,126 | 99.2 | **1.001** | 208 | **2** | 3 | **0.7** | 2.2 | 157,344 | 0 |
| **CABOG** | **Basic flow** | B(21, 100x) | 22,071 | 21,315 | 22,335 | 21,484 | 100.0 | 1.008 | 352 | 3 | 4 | 3.5 | 0.5 | 77,638 | 4,667 |
|  | **HGA Preprocessing** | AP(21, 2, 50x) | 23,089 | 22,579 | 22,871 | 22,584 | 99.7 | 1.001 | 374 | 3 | 2 | 3.4 | 0.6 | 77,409 | 4,615 |
|  |  | C(21, 2, 50x) | 63,342 | 61,017 | 63,342 | 61,017 | 99.9 | 1.000 | 149 | 4 | 2 | 1.9 | 0.3 | 77,986 | 4,814 |
|  | **HGA re-assembly Contigs** | HGA(31, C(21, 8, 12x)) | 287,637 | 203,401 | 287,637 | 203,401 | 100.7 | 1.013 | 472 | 12 | 5 | 25.4 | 2.4 | 168,639 | 4,914 |
|  |  | HGA(21, M(21, 8, 12x)) | 278,455 | 187,335 | 278,455 | 187,335 | 100.6 | 1.016 | 546 | 14 | 8 | 30.2 | 2.0 | 180,878 | 4,910 |
|  | **GAGE Contigs** | GAGE-B | 8,655 | 8,294 | 8,716 | 8,344 | 97.5 | 1.030 | 857 | 122 | 5 | 4.2 | 0.7 | 75,046 | 4,148 |
|  | **HGA re-assembly scaffolds** | HGA(31, C(21, 8, 12x)) | 302,807 | 203,401 | 302,807 | 210,004 | 100.7 | 1.013 | 468 | 12 | 6 | 25.5 | 2.5 | 168,639 | 0 |
|  |  | HGA(21, M(21, 8, 12x)) | 278,455 | 187,335 | 278,455 | 187,335 | 100.6 | 1.016 | 545 | 14 | 8 | 28.9 | 2.0 | 180,878 | 0 |
|  | **GAGE scaffolds** | GAGE-B | 9,070 | 8,413 | 9,127 | 8,433 | 97.5 | 1.030 | 847 | 131 | 5 | 4.2 | 0.8 | 75,046 | 0 |
| **MIRA** | **Basic flow** | B(21, 100x) | 6,089 | 4,779 | 15,167 | 12,051 | 100.8 | 2.195 | 4,717 | 1,476 | 22 | 4.9 | 0.9 | 167,341 | 4,594 |
|  | **HGA Preprocessing** | AP(21, 2, 50x) | 3,246 | 2,822 | 8,642 | 7,357 | 100.7 | 2.210 | 7,000 | 1,296 | 27 | 8.1 | 1.2 | 192,680 | 4,339 |
|  |  | C(21, 2, 50x) | 36,144 | 35,721 | 36,347 | 35,910 | 100.6 | 1.011 | 338 | 14 | 5 | 3.7 | 0.5 | 85,563 | 4,753 |
|  | **HGA re-assembly Contigs** | HGA(31, C(21, 1, 100x)) | 273,781 | 205,053 | 273,781 | 205,053 | 100.7 | 1.005 | 474 | 6 | 3 | 12.3 | 1.0 | 210,953 | 4,918 |
|  |  | HGA(21, M(21, 2, 50x)) | 388,304 | **308,604** | 388,304 | **308,604** | 100.7 | 1.016 | 528 | 13 | 7 | 31.1 | 2.2 | 180,151 | **4,924** |
|  | **GAGE Contigs** | GAGE-B | 81,728 | 64,678 | 114,083 | 74,987 | **100.8** | 1.150 | 1,760 | 2,358 | 35 | 4.2 | 0.6 | 45,545 | 4,869 |
|  | **HGA re-assembly scaffolds** | HGA(31, C(21, 1, 100x)) | 274,707 | 210,037 | 274,707 | 225,649 | 100.7 | 1.015 | 471 | 9 | 3 | 13.1 | 1.2 | 156,740 | 0 |
|  |  | HGA(21, M(21, 2, 50x)) | 388,304 | **308,604** | 388,304 | **308,604** | 100.7 | 1.016 | 527 | 12 | 7 | 31.0 | 2.4 | 180,151 | 0 |
|  | **GAGE scaffolds** | GAGE-B | - | - | - | - | - | - | - | - | - | - | - | - | - |
| **MaSuRCA** | **Basic flow** | B(101, 100x) | 11,889 | 11,524 | 12,466 | 12,075 | 100.2 | 1.056 | 644 | 5 | 2 | 2.8 | 0.4 | 80,838 | 4,569 |
|  | **HGA Preprocessing** | AP(91, 2, 50x) | 23,342 | 22,308 | 23,893 | 23,547 | 99.9 | 1.024 | 332 | 6 | 3 | 2.4 | 0.4 | 76,301 | 4,701 |
|  |  | C(71, 2, 50x) | 140,334 | 121,303 | 140,334 | 121,303 | 100.1 | 1.014 | 89 | 5 | 3 | 2.7 | 0.4 | 17,310 | 4,867 |
|  | **HGA re-assembly Contigs** | HGA(91, C(21, 1, 100x)) | 322,474 | 209,888 | 322,474 | 233,055 | 100.7 | 1.012 | 148 | 7 | 3 | 4.8 | 0.8 | 61,909 | 4,920 |
|  |  | HGA(61, M(21, 1, 100x)) | 340,250 | 272,329 | 340,250 | 272,329 | 100.7 | 1.014 | 287 | 6 | 3 | 3.9 | **0.4** | 102,307 | 4,920 |
|  | **GAGE Contigs** | GAGE-B(99) | 36,211 | 35,496 | 38,240 | 37,156 | 99.7 | 1.067 | 326 | 70 | **2** | 2.9 | 0.6 | 72,188 | 4,790 |
|  | **HGA re-assembly scaffolds** | HGA(91, C(21, 1, 100x)) | 340,280 | 209,888 | 340,280 | 233,055 | 100.7 | 1.012 | 147 | 8 | 3 | 5.4 | 0.9 | 61,909 | 0 |
|  |  | HGA(61, M(21, 1, 100x)) | 340,250 | 272,329 | 340,250 | 272,329 | 100.7 | 1.014 | 286 | 7 | 3 | 4.5 | 0.5 | 102,307 | 0 |
|  | **GAGE scaffolds** | GAGE-B(99) | 36,211 | 35,496 | 38,240 | 37,156 | 99.7 | 1.067 | 325 | 70 | **2** | 2.9 | 0.6 | 72,188 | 0 |
| **SGA** | **Basic flow** | B(91, 100x) | 28,150 | 26,835 | 28,371 | 27,256 | 100.7 | 1.016 | 488 | 71 | 2 | 2.0 | 0.4 | 84,449 | 4,713 |
|  | **HGA Preprocessing** | AP(61, 2, 50x) | 18,850 | 18,371 | 19,115 | 18,675 | 100.5 | 1.011 | 606 | 42 | 2 | 1.9 | 0.4 | 83,864 | 4,580 |
|  |  | C(21, 2, 50x) | 79,650 | 76,525 | 79,650 | 76,525 | 100.5 | 1.003 | 146 | 4 | 2 | 2.4 | 0.4 | 71,528 | 4,842 |
|  | **HGA re-assembly Contigs** | HGA(31, C(51, 8, 12x)) | 363,745 | 205,054 | 363,745 | 205,054 | 100.7 | 1.005 | 463 | 12 | 4 | 20.1 | 1.6 | 210,953 | 4,919 |
|  |  | HGA(21, M(71, 8, 12x)) | 278,376 | 193,542 | 278,376 | 209,488 | 100.6 | 1.014 | 553 | 11 | 9 | 31.2 | 2.4 | 192,021 | 4,910 |
|  | **GAGE Contigs** | GAGE-B(65) | 12,696 | 12,320 | 13,299 | 12,834 | 100.7 | 1.045 | 1,117 | 180 | 4 | 2.0 | 0.4 | 74,740 | 4,467 |
|  | **HGA re-assembly scaffolds** | HGA(31, C(51, 8, 12x)) | 363,745 | 210,039 | 363,745 | 225,649 | 100.7 | 1.016 | 460 | 14 | 4 | 22.9 | 1.8 | 156,740 | 0 |
|  |  | HGA(21, M(71, 8, 12x)) | 278,376 | 193,542 | 278,376 | 209,488 | 100.6 | 1.014 | 552 | 11 | 9 | 31.4 | 2.5 | 192,021 | 0 |
|  | **GAGE scaffolds** | GAGE-B(65) | 12,834 | 12,472 | 13,299 | 12,834 | 100.7 | 1.031 | 815 | 10 | **2** | 1.8 | **0.4** | 67,076 | 0 |
| **SOAPd2** | **Basic flow** | B(51, 100x) | 38,506 | 36,994 | 39,634 | 38,168 | 100.6 | 1.002 | 275 | 2 | 2 | 1.4 | 0.4 | 83,782 | 4,772 |
|  | **HGA Preprocessing** | AP(41, 2, 50x) | 15,745 | 15,319 | 15,782 | 15,405 | 100.4 | 1.002 | 616 | 2 | 1 | 2.2 | 0.4 | 83,569 | 4,508 |
|  |  | C(51, 2, 50x) | 67,061 | 62,457 | 67,061 | 65,560 | 100.4 | 1.000 | 188 | 3 | 3 | 2.4 | 0.4 | 83,293 | 4,830 |
|  | **HGA re-assembly Contigs** | HGA(81, C(41, 1, 100x)) | 274,727 | 210,215 | 274,727 | 210,215 | 100.7 | 1.016 | 198 | 7 | 3 | 3.8 | 0.5 | 54,982 | 4,911 |
|  |  | HGA(21, M(61, 4, 25x)) | 278,409 | 194,290 | 278,409 | 209,503 | 100.6 | 1.017 | 549 | 12 | 14 | 32.1 | 2.9 | 192,021 | 4,909 |
|  | **GAGE Contigs** | GAGE-B(47) | 131,561 | 113,272 | 131,561 | 113,272 | 100.6 | 1.014 | **113** | 5 | 19 | 2.2 | 0.7 | **17,490** | 4,877 |
|  | **HGA re-assembly scaffolds** | HGA(81, C(41, 1, 100x)) | 274,727 | 210,215 | 274,727 | 210,215 | 100.7 | 1.016 | 197 | 8 | 3 | 4.4 | 0.6 | 54,982 | 0 |
|  |  | HGA(21, M(61, 4, 25x)) | 278,409 | 194,290 | 278,409 | 209,503 | 100.5 | 1.017 | 548 | 12 | 14 | 31.3 | 2.9 | 194,332 | 0 |
|  | **GAGE scaffolds** | GAGE-B(47) | 147,990 | 147,162 | 152,604 | 147,162 | 100.6 | 1.014 | **101** | 5 | 31 | 2.2 | 0.7 | **17,490** | 0 |
| **SPAdes** | **Basic flow** | B(31, 100x) | 231,671 | 193,002 | 253,859 | 193,002 | 100.5 | 1.003 | 500 | 8 | 4 | 7.4 | 0.6 | 222,954 | 4,900 |
|  | **HGA Preprocessing** | AP(21, 2, 50x) | 128,044 | 116,659 | 131,477 | 116,659 | 100.4 | 1.014 | 371 | 9 | 5 | 15.1 | 1.1 | 108,673 | 4,872 |
|  |  | C(61, 4, 25x) | 140,162 | 131,364 | 144,325 | 131,364 | 100.5 | 1.003 | 97 | 7 | 4 | 3.5 | 0.5 | 72,070 | 4,889 |
|  | **HGA re-assembly Contigs** | HGA(71, C(21, 8, 12x)) | 372,707 | 234,759 | 372,707 | 234,759 | 100.7 | 1.016 | 224 | 17 | 7 | 8.5 | 1.3 | 72,271 | 4,916 |
|  |  | HGA(21, M(81, 8, 12x)) | **474,754** | 206,114 | **474,754** | 206,114 | 100.6 | 1.015 | 530 | 14 | 10 | 28.0 | 2.3 | 192,329 | 4,923 |
|  | **GAGE Contigs** | GAGE-B(33,55,65,75,85,99) | 215,400 | 209,894 | 220,161 | 209,894 | **100.8** | 1.018 | 908 | 20 | 5 | 4.7 | 0.9 | 259,449 | 4,913 |
|  | **HGA re-assembly scaffolds** | HGA(71, C(21, 8, 12x)) | 372,707 | 234,759 | 372,707 | 234,759 | 100.7 | 1.017 | 222 | 18 | 7 | 8.8 | 1.3 | 72,271 | 0 |
|  |  | HGA(21, M(81, 8, 12x)) | **474,754** | 206,114 | 474,754 | 206,114 | 100.6 | 1.015 | 529 | 14 | 10 | 26.7 | 2.3 | 192,329 | 0 |
|  | **GAGE scaffolds** | GAGE-B(33,55,65,75,85,99) | 215,400 | 209,894 | 220,161 | 209,894 | **100.8** | 1.018 | 908 | 20 | 5 | 4.7 | 0.9 | 264,835 | 0 |
| **Velvet** | **Basic flow** | B(91, 100x) | 23,440 | 22,003 | 23,440 | 22,003 | 100.0 | 1.006 | 370 | 30 | 3 | 5.5 | 0.9 | 67,345 | 4,621 |
|  | **HGA Preprocessing** | AP(71, 2, 50x) | 16,952 | 16,481 | 17,060 | 16,455 | 99.5 | 1.004 | 537 | 37 | 3 | 5.1 | 0.9 | 82,973 | 4,471 |
|  |  | C(71, 2, 50x) | 54,548 | 53,765 | 54,548 | 53,765 | 100.1 | 1.001 | 220 | 2 | 3 | 2.7 | 0.6 | 83,376 | 4,778 |
|  | **HGA re-assembly Contigs** | HGA(31, C(101, 8, 12x)) | 273,781 | 205,054 | 273,781 | 205,054 | 100.6 | 1.003 | 473 | 8 | 5 | 10.7 | 0.6 | 222,852 | 4,917 |
|  |  | HGA(31, M(91, 8, 12x)) | 452,190 | 210,096 | 452,190 | 225,649 | 100.6 | 1.014 | 454 | 11 | 5 | 13.0 | 1.4 | 168,034 | 4,920 |
|  | **GAGE Contigs** | GAGE-B(97) | 47,327 | 40,129 | 48,155 | 41,485 | 100.4 | 1.011 | 279 | 76 | 3 | 2.5 | 0.6 | 62,440 | 4,746 |
|  | **HGA re-assembly scaffolds** | HGA(31, C(101, 8, 12x)) | 274,707 | 210,039 | 274,707 | 225,649 | 100.6 | 1.013 | 471 | 9 | 5 | 11.1 | 0.7 | 168,639 | 0 |
|  |  | HGA(31, M(91, 8, 12x)) | 466,584 | 225,649 | **571,267** | 232,801 | 100.6 | 1.028 | 453 | 12 | 5 | 13.0 | 1.4 | 168,034 | 0 |
|  | **GAGE scaffolds** | GAGE-B(97) | 70,983 | 44,950 | 70,983 | 45,957 | 100.4 | 1.011 | 229 | 124 | 19 | 2.5 | 0.6 | 62,427 | 0 |

**Supplementary Table 7:** 100bp HiSeq read assemblies of *M. abscessus*, with reference genome size of 5,090,401bp and 4,992 genes. For metric and assembly flow descriptions see beginning of section.

| **Assembler** | **Type** | **Flow** | **N50** | **NA50** | **NG50** | **NGA50** | **Genome** | **Duplication** | **#** | **# mis-** | **Local mis-** | **# mismatches** | **# indels** | **Unaligned** | **#** |
| --- | --- | --- | --- | --- | --- | --- | --- | --- | --- | --- | --- | --- | --- | --- | --- |
|  |  |  |  |  |  |  | **fraction** | **ratio** | **contigs** | **assemblies** | **assemblies** | **per 100KB** | **per 100KB** | **length** | **Genes** |
| **ABySS** | **Basic flow** | B(41, 115x) | 148,261 | 147,341 | 148,261 | 148,137 | 99.9 | 1.005 | 85 | 3 | 9 | 5.5 | 1.1 | 66,112 | 4,901 |
|  | **HGA Preprocessing** | AP(31, 2, 57x) | 127,253 | 123,841 | 127,253 | 123,841 | 99.8 | 1.004 | 102 | 3 | 13 | 5.5 | 1.8 | 66,708 | 4,886 |
|  |  | C(31, 2, 57x) | 125,738 | 105,633 | 125,738 | 125,738 | 99.6 | 1.000 | 126 | 2 | 16 | 4.9 | 2.0 | 77,908 | 4,865 |
|  | **HGA re-assembly Contigs** | HGA(31, C(61, 8, 14x)) | 321,915 | 231,737 | 321,915 | 247,233 | 99.8 | 1.017 | 98 | 13 | 7 | 11.1 | 1.4 | 9,431 | 4,911 |
|  |  | HGA(31, M(91, 2, 57x)) | 336,297 | 190,184 | 336,297 | 233,884 | 99.8 | 1.006 | 94 | 6 | 12 | 8.5 | 0.9 | 63,512 | 4,915 |
|  | **GAGE Contigs** | GAGE-B(53) | 119,446 | 111,763 | 119,446 | 115,738 | 99.8 | 1.003 | 124 | 4 | 8 | 3.9 | 1.5 | 66,194 | 4,875 |
|  | **HGA re-assembly scaffolds** | HGA(31, C(61, 8, 14x)) | 321,915 | 260,299 | 321,915 | 260,299 | 99.9 | 1.017 | 94 | 13 | 9 | 11.8 | 1.4 | 9,431 | 0 |
|  |  | HGA(31, M(91, 2, 57x)) | 336,297 | 190,184 | 336,297 | 233,884 | 99.8 | 1.017 | 92 | 8 | 13 | 9.0 | 1.1 | 9,299 | 0 |
|  | **GAGE scaffolds** | GAGE-B(53) | 147,937 | 127,410 | 147,937 | 127,410 | 99.8 | 1.015 | 77 | 11 | 26 | 9.8 | 2.1 | 11,899 | 0 |
| **CABOG** | **Basic flow** | B(21, 115x) | 13,923 | 13,366 | 13,670 | 13,087 | 97.4 | 1.006 | 578 | 3 | 4 | 5.6 | 3.8 | 78,651 | 4,368 |
|  | **HGA Preprocessing** | AP(21, 2, 57x) | 10,824 | 10,602 | 10,705 | 10,503 | 97.9 | 1.002 | 703 | 3 | 5 | 6.7 | 0.8 | 76,078 | 4,278 |
|  |  | C(21, 2, 57x) | 20,502 | 19,499 | 19,952 | 19,415 | 98.3 | 1.000 | 394 | 4 | 7 | 8.8 | 1.0 | 77,038 | 4,548 |
|  | **HGA re-assembly Contigs** | HGA(31, C(21, 2, 57x)) | 364,306 | 247,266 | 364,306 | 247,266 | 99.9 | 1.017 | 89 | 10 | 10 | 16.2 | 1.4 | 9,360 | 4,914 |
|  |  | HGA(31, M(21, 4, 28x)) | 364,231 | 278,346 | 364,231 | 278,346 | 99.8 | 1.016 | 82 | 9 | 9 | 11.0 | 0.9 | 9,299 | 4,910 |
|  | **GAGE Contigs** | GAGE-B | 81,416 | 78,165 | 81,416 | 78,165 | 99.5 | 1.004 | 127 | 7 | 6 | 8.6 | 5.8 | 65,602 | 4,827 |
|  | **HGA re-assembly scaffolds** | HGA(31, C(21, 2, 57x)) | 364,306 | 247,266 | 364,306 | 247,266 | 99.9 | 1.017 | 88 | 10 | 10 | 16.2 | 1.6 | 9,360 | 0 |
|  |  | HGA(31, M(21, 4, 28x)) | 364,231 | 278,346 | 364,231 | 278,346 | 99.8 | 1.016 | 80 | 9 | 9 | 11.1 | 1.1 | 9,299 | 0 |
|  | **GAGE scaffolds** | GAGE-B | 94,359 | 89,623 | 94,359 | 89,623 | 99.5 | **1.004** | 109 | 10 | 13 | 8.8 | 6.2 | 65,564 | 0 |
| **MIRA** | **Basic flow** | B(21, 115x) | 8,616 | 8,242 | 15,845 | 15,260 | 99.9 | 2.043 | 2,805 | 75 | 7 | 8.0 | 1.0 | 166,010 | 4,534 |
|  | **HGA Preprocessing** | AP(21, 2, 57x) | 5,811 | 5,583 | 10,086 | 9,823 | 99.9 | 2.047 | 3,654 | 77 | 8 | 10.9 | 1.0 | 168,031 | 4,229 |
|  |  | C(21, 2, 57x) | 120,187 | 119,730 | 120,187 | 119,730 | 99.8 | 1.004 | 105 | 6 | 7 | 9.3 | 0.7 | 66,133 | 4,879 |
|  | **HGA re-assembly Contigs** | HGA(31, C(21, 8, 14x)) | 290,400 | 214,205 | 290,400 | 214,205 | 99.9 | 1.017 | 89 | 16 | 12 | 17.6 | 1.3 | 9,299 | 4,915 |
|  |  | HGA(31, M(21, 8, 14x)) | 290,400 | 212,994 | 290,400 | 212,994 | 99.9 | 1.019 | 75 | 21 | 13 | 23.7 | 1.7 | 9,299 | 4,915 |
|  | **GAGE Contigs** | GAGE-B | 129,228 | 119,746 | 147,272 | 129,075 | **100.0** | 1.170 | 3,293 | 452 | 6 | 6.1 | 0.7 | 59,775 | 4,902 |
|  | **HGA re-assembly scaffolds** | HGA(31, C(21, 8, 14x)) | 304,450 | 214,205 | 304,450 | 214,205 | 99.9 | 1.017 | 88 | 16 | 13 | 17.6 | 1.3 | 9,299 | 0 |
|  |  | HGA(31, M(21, 8, 14x)) | 290,400 | 212,994 | 290,400 | 212,994 | 99.9 | 1.019 | 73 | 21 | 15 | 24.4 | 1.8 | 9,299 | 0 |
|  | **GAGE scaffolds** | GAGE-B | - | - | - | - | - | - | - | - | - | - | - | - | - |
| **MaSuRCA** | **Basic flow** | B(81, 115x) | 14,899 | 13,999 | 15,406 | 14,677 | 99.8 | 1.044 | 544 | 2 | 8 | 21.2 | 1.3 | 79,393 | 4,630 |
|  | **HGA Preprocessing** | AP(31, 2, 28x) | 28,219 | 27,638 | 29,139 | 28,583 | 99.2 | 1.029 | 291 | 6 | 4 | 6.7 | 0.7 | 77,561 | 4,770 |
|  |  | C(71, 2, 57x) | 167,952 | 146,852 | 167,952 | 146,852 | 99.6 | 1.005 | 71 | 4 | 2 | 5.6 | 1.0 | 54,211 | 4,893 |
|  | **HGA re-assembly Contigs** | HGA(31, C(61, 2, 57x)) | 508,685 | **343,659** | 508,685 | **343,659** | 99.9 | 1.016 | 77 | 7 | 5 | 10.4 | 0.8 | 9,299 | **4,927** |
|  |  | HGA(31, M(81, 4, 28x)) | **551,349** | 313,850 | **551,350** | 313,850 | 99.9 | 1.016 | 80 | 10 | 4 | 9.8 | 1.2 | 9,299 | 4,922 |
|  | **GAGE Contigs** | GAGE-B(89) | 246,830 | 187,809 | 246,830 | 187,809 | 99.9 | 1.018 | **66** | 6 | 2 | 49.7 | 4.2 | **102** | 4,910 |
|  | **HGA re-assembly scaffolds** | HGA(31, C(61, 2, 57x)) | 508,685 | **343,659** | 508,685 | **343,659** | 99.9 | 1.016 | 77 | 7 | 5 | 10.4 | 0.8 | 9,299 | 0 |
|  |  | HGA(31, M(81, 4, 28x)) | **551,350** | 313,850 | **551,350** | 313,850 | 99.9 | 1.016 | 78 | 11 | 5 | 9.8 | 1.2 | 9,299 | 0 |
|  | **GAGE scaffolds** | GAGE-B(89) | 246,830 | 187,809 | 246,830 | 187,809 | **100.0** | 1.018 | **59** | 11 | 3 | 50.4 | 4.3 | **102** | 0 |
| **SGA** | **Basic flow** | B(41, 115x) | 29,477 | 28,291 | 29,507 | 28,728 | 99.8 | 1.004 | 371 | 2 | 2 | 1.1 | 0.4 | 78,127 | 4,725 |
|  | **HGA Preprocessing** | AP(31, 2, 57x) | 21,090 | 20,597 | 21,224 | 20,854 | 99.6 | 1.003 | 512 | 3 | 1 | 1.2 | 0.3 | 78,207 | 4,611 |
|  |  | C(21, 2, 57x) | 63,311 | 55,471 | 63,311 | 58,424 | 99.6 | 1.000 | 187 | 2 | 3 | 1.5 | 0.4 | 77,935 | 4,819 |
|  | **HGA re-assembly Contigs** | HGA(31, C(71, 8, 14x)) | 344,886 | 232,801 | 344,886 | 232,801 | 99.8 | 1.005 | 100 | 6 | 10 | 8.3 | 1.1 | 63,512 | 4,909 |
|  |  | HGA(31, M(61, 4, 28x)) | 286,710 | 231,671 | 286,710 | 231,671 | 99.8 | 1.006 | 101 | 10 | 7 | 22.8 | 1.7 | 63,512 | 4,909 |
|  | **GAGE Contigs** | GAGE-B(65) | 28,734 | 27,712 | 28,781 | 27,760 | 99.8 | 1.006 | 378 | **3** | **1** | **1.2** | **0.4** | 66,157 | 4,712 |
|  | **HGA re-assembly scaffolds** | HGA(31, C(71, 8, 14x)) | 344,886 | 232,801 | 344,886 | 232,801 | 99.8 | 1.016 | 97 | 8 | 11 | 9.8 | 1.5 | 9,299 | 0 |
|  |  | HGA(31, M(61, 4, 28x)) | 313,536 | 278,346 | 313,536 | 278,346 | 99.8 | 1.017 | 97 | 12 | 9 | 14.5 | 1.1 | 9,299 | 0 |
|  | **GAGE scaffolds** | GAGE-B(65) | 28,734 | 27,712 | 28,781 | 27,760 | 99.7 | 1.005 | 363 | **3** | **1** | **1.2** | **0.4** | 66,157 | 0 |
| **SOAPd2** | **Basic flow** | B(61, 115x) | 20,458 | 20,325 | 20,620 | 20,395 | 99.8 | 1.006 | 481 | 4 | 1 | 1.9 | 0.3 | 66,698 | 4,593 |
|  | **HGA Preprocessing** | AP(51, 2, 57x) | 17,073 | 16,361 | 17,155 | 16,535 | 99.8 | 1.004 | 572 | 3 | 1 | 1.4 | 0.3 | 72,611 | 4,516 |
|  |  | C(51, 2, 57x) | 58,445 | 53,919 | 58,445 | 54,497 | 99.7 | 1.003 | 199 | 3 | 2 | 1.5 | 0.4 | 66,556 | 4,811 |
|  | **HGA re-assembly Contigs** | HGA(71, C(21, 8, 14x)) | 365,034 | 234,293 | 365,034 | 234,293 | 99.9 | 1.016 | 76 | 8 | 8 | 5.2 | 0.9 | 6,405 | 4,919 |
|  |  | HGA(31, M(81, 4, 28x)) | 360,431 | 233,090 | 360,431 | 233,090 | 99.9 | 1.005 | 103 | 10 | 10 | 8.9 | 1.1 | 63,512 | 4,907 |
|  | **GAGE Contigs** | GAGE-B(49) | 148,639 | 144,505 | 148,639 | 147,199 | 99.9 | 1.014 | 91 | 9 | 15 | 1.8 | 0.7 | 17,699 | 4,888 |
|  | **HGA re-assembly scaffolds** | HGA(71, C(21, 8, 14x)) | 365,034 | 243,843 | 365,034 | 243,843 | 99.9 | 1.016 | 73 | 8 | 9 | 6.1 | 0.9 | 6,405 | 0 |
|  |  | HGA(31, M(81, 4, 28x)) | 360,431 | 233,090 | 360,431 | 233,090 | 99.9 | 1.016 | 101 | 12 | 10 | 9.3 | 1.1 | 9,299 | 0 |
|  | **GAGE scaffolds** | GAGE-B(49) | 150,256 | 147,925 | 150,256 | 147,925 | 99.9 | 1.014 | 85 | 10 | 20 | 1.8 | 0.7 | 17,699 | 0 |
| **SPAdes** | **Basic flow** | B(31, 115x) | 271,028 | 209,890 | 271,028 | 209,890 | 99.8 | 1.005 | 117 | 4 | 11 | 5.4 | 0.8 | 63,512 | 4,903 |
|  | **HGA Preprocessing** | AP(31, 2, 57x) | 188,056 | 164,996 | 188,056 | 164,996 | 99.7 | 1.003 | 116 | 4 | 13 | 5.0 | 0.8 | 71,627 | 4,895 |
|  |  | C(31, 4, 28x) | 231,735 | 185,600 | 231,735 | 185,600 | 99.8 | 1.011 | 97 | 3 | 16 | 4.4 | 1.0 | 68,268 | 4,902 |
|  | **HGA re-assembly Contigs** | HGA(31, C(61, 8, 14x)) | 397,162 | 209,890 | 397,162 | 209,890 | 99.8 | 1.006 | 93 | 15 | 6 | 15.8 | 1.8 | 63,512 | 4,916 |
|  |  | HGA(61, M(21, 8, 14x)) | 428,516 | 225,656 | 428,516 | 225,656 | 99.9 | 1.017 | 81 | 13 | 8 | 13.2 | 1.4 | 8,404 | 4,921 |
|  | **GAGE Contigs** | GAGE-B(33,55,65,75,85,99) | 150,258 | 147,871 | 150,258 | 147,871 | 99.9 | 1.006 | 96 | 4 | 5 | 2.2 | 0.6 | 56,101 | 4,897 |
|  | **HGA re-assembly scaffolds** | HGA(31, C(61, 8, 14x)) | 428,650 | 209,890 | 428,650 | 209,890 | 99.9 | 1.016 | 89 | 16 | 9 | 16.4 | 1.9 | 9,299 | 0 |
|  |  | HGA(61, M(21, 8, 14x)) | 548,170 | 225,656 | 548,170 | 225,656 | 99.9 | 1.017 | 79 | 14 | 9 | 14.4 | 1.5 | 8,404 | 0 |
|  | **GAGE scaffolds** | GAGE-B(33,55,65,75,85,99) | 215,724 | 209,754 | 223,056 | 213,402 | **100.0** | 1.069 | 70 | 8 | 7 | 5.0 | 1.5 | 56,103 | 0 |
| **Velvet** | **Basic flow** | B(51, 115x) | 53,593 | 47,961 | 53,706 | 47,961 | 99.7 | 1.006 | 169 | 6 | 9 | 4.5 | 0.9 | 54,213 | 4,809 |
|  | **HGA Preprocessing** | AP(41, 2, 57x) | 35,165 | 34,625 | 35,165 | 34,889 | 99.5 | 1.002 | 277 | 6 | 8 | 3.6 | 0.8 | 71,656 | 4,719 |
|  |  | C(31, 4, 28x) | 162,336 | 154,189 | 162,336 | 156,735 | 99.6 | 1.015 | 81 | 9 | 18 | 4.6 | 0.9 | 11,899 | 4,885 |
|  | **HGA re-assembly Contigs** | HGA(61, C(21, 8, 14x)) | 418,776 | 225,668 | 418,776 | 233,193 | 99.9 | 1.017 | **66** | 12 | 12 | 12.5 | 0.9 | 4,034 | 4,919 |
|  |  | HGA(31, M(51, 1, 115x)) | 421,917 | 278,346 | 421,917 | 278,346 | 99.9 | 1.017 | 92 | 8 | 11 | 8.2 | 1.1 | 9,299 | 4,914 |
|  | **GAGE Contigs** | GAGE-B(49) | 60,955 | 58,337 | 61,615 | 58,337 | 99.8 | **1.003** | 155 | 4 | 9 | 3.2 | 0.7 | 66,313 | 4,822 |
|  | **HGA re-assembly scaffolds** | HGA(61, C(21, 8, 14x)) | 418,776 | 225,668 | 418,776 | 233,193 | 99.9 | 1.017 | 64 | 13 | 12 | 12.5 | 0.9 | 4,034 | 0 |
|  |  | HGA(31, M(51, 1, 115x)) | 421,917 | 278,346 | 421,917 | 278,346 | 99.9 | 1.017 | 92 | 8 | 11 | 8.2 | 1.1 | 9,299 | 0 |
|  | **GAGE scaffolds** | GAGE-B(49) | 248,309 | 147,755 | 262,034 | 147,755 | 99.8 | 1.019 | 74 | 9 | 82 | 3.2 | 0.8 | 201 | 0 |

**Supplementary Table 8:** 250bp MiSeq read assemblies of *V. cholera*, with reference genome size of 4,033,464bp and 3,693 genes. For metric and assembly flow descriptions see beginning of section.

| **Assembler** | **Type** | **Flow** | **N50** | **NA50** | **NG50** | **NGA50** | **Genome** | **Duplication** | **#** | **# mis-** | **Local mis-** | **# mismatches** | **# indels** | **Unaligned** | **#** |
| --- | --- | --- | --- | --- | --- | --- | --- | --- | --- | --- | --- | --- | --- | --- | --- |
|  |  |  |  |  |  |  | **fraction** | **ratio** | **contigs** | **assemblies** | **assemblies** | **per 100KB** | **per 100KB** | **length** | **Genes** |
| **ABySS** | **Basic flow** | B(91, 100x) | 92,024 | 92,024 | 85,271 | 85,271 | 101.5 | 1.002 | 213 | 6 | 2 | 3.3 | 2.9 | 767 | 3,495 |
|  | **HGA Preprocessing** | AP(61, 2, 50x) | 34,710 | 34,710 | 34,525 | 34,525 | 100.6 | 1.002 | 393 | 3 | 1 | 4.1 | 2.8 | 1,166 | 3,367 |
|  |  | C(81, 2, 50x) | 71,270 | 71,270 | 68,996 | 68,996 | 100.8 | 1.000 | 214 | 5 | 1 | 5.1 | 2.9 | 915 | 3,433 |
|  | **HGA re-assembly Contigs** | HGA(101, C(51, 2, 50x)) | 243,230 | 199,213 | 243,230 | 199,213 | 101.8 | 1.002 | 232 | 10 | 6 | 8.0 | 2.9 | 57,060 | 3,554 |
|  |  | HGA(101, M(31, 2, 50x)) | 243,230 | 153,070 | 243,230 | 153,070 | 101.8 | 1.002 | 236 | 10 | 5 | 8.4 | 3.3 | 57,576 | 3,550 |
|  | **GAGE Contigs** | GAGE-B(65) | 60,973 | 60,473 | 60,473 | 60,272 | 101.1 | 1.001 | 267 | 2 | 0 | 3.3 | 2.7 | 1,062 | 3,436 |
|  | **HGA re-assembly scaffolds** | HGA(101, C(51, 2, 50x)) | 243,230 | 199,213 | 243,230 | 199,213 | 101.8 | 1.002 | 231 | 10 | 7 | 8.2 | 2.9 | 57,060 | 0 |
|  |  | HGA(101, M(31, 2, 50x)) | 243,230 | 153,070 | 243,230 | 153,070 | 101.8 | 1.002 | 235 | 10 | 6 | 8.5 | 3.3 | 57,576 | 0 |
|  | **GAGE scaffolds** | GAGE-B(65) | 60,973 | 60,473 | 60,473 | 60,272 | 101.1 | 1.001 | 267 | **2** | **0** | 3.3 | **2.7** | 1,062 | 0 |
| **CABOG** | **Basic flow** | B(21, 100x) | 24,874 | 24,874 | 22,450 | 22,450 | 97.6 | 1.003 | 285 | 10 | 6 | 8.4 | 3.1 | 49 | 3,288 |
|  | **HGA Preprocessing** | AP(21, 2, 50x) | 20,379 | 20,352 | 18,978 | 18,646 | 97.2 | 1.003 | 348 | 9 | 4 | 7.5 | 3.0 | 106 | 3,242 |
|  |  | C(21, 4, 25x) | 55,962 | 54,574 | 50,415 | 49,407 | 98.2 | 1.000 | 168 | 6 | 5 | 6.3 | 3.0 | 0 | 3,370 |
|  | **HGA re-assembly Contigs** | HGA(61, C(21, 8, 12x)) | 180,921 | 174,450 | 180,921 | 174,450 | 101.6 | 1.001 | 578 | 10 | 6 | 10.2 | 3.4 | 185,292 | 3,536 |
|  |  | HGA(51, M(21, 2, 50x)) | 211,814 | 195,600 | 211,814 | 195,600 | 101.6 | 1.002 | 662 | 9 | 6 | 12.8 | 3.6 | 219,922 | 3,545 |
|  | **GAGE Contigs** | GAGE-B | 33,710 | 33,710 | 32,790 | 32,784 | 100.8 | 1.011 | 241 | 17 | 7 | 8.2 | 3.4 | 5,249 | 3,401 |
|  | **HGA re-assembly scaffolds** | HGA(61, C(21, 8, 12x)) | 199,144 | 196,419 | 199,144 | 196,419 | 101.6 | 1.001 | 575 | 10 | 9 | 10.6 | 3.4 | 185,292 | 0 |
|  |  | HGA(51, M(21, 2, 50x)) | 211,814 | 195,600 | 211,814 | 195,600 | 101.6 | 1.002 | 662 | 9 | 6 | 12.8 | 3.6 | 219,922 | 0 |
|  | **GAGE scaffolds** | GAGE-B | 33,710 | 33,710 | 32,790 | 32,784 | 100.8 | 1.011 | 241 | 17 | 7 | 8.2 | 3.4 | 5,249 | 0 |
| **MIRA** | **Basic flow** | B(21, 100x) | 15,464 | 14,740 | 34,378 | 32,422 | 102.5 | 2.102 | 2,056 | 171 | 16 | 16.2 | 3.6 | 24,175 | 3,496 |
|  | **HGA Preprocessing** | AP(21, 2, 50x) | 6,703 | 6,578 | 16,784 | 16,663 | 102.3 | 2.132 | 3,243 | 114 | 15 | 15.9 | 4.0 | 16,801 | 3,294 |
|  |  | C(21, 2, 50x) | 113,674 | 107,406 | 112,602 | 107,406 | 101.4 | 1.002 | 127 | 11 | 5 | 6.5 | 3.1 | 5,461 | 3,510 |
|  | **HGA re-assembly Contigs** | HGA(101, C(21, 2, 50x)) | 268,342 | 249,690 | 268,342 | 249,690 | 101.9 | 1.002 | 209 | 16 | 4 | 14.2 | 3.3 | 57,001 | 3,569 |
|  |  | HGA(31, M(21, 8, 12x)) | **443,239** | 153,279 | **443,239** | 259,073 | 102.3 | 1.005 | 824 | 31 | 6 | 26.0 | 4.9 | 288,613 | 3,578 |
|  | **GAGE Contigs** | GAGE-B | 112,926 | 106,563 | 112,926 | 108,689 | 102.3 | 1.029 | 431 | 106 | 12 | 9.5 | 3.8 | 23,688 | 3,559 |
|  | **HGA re-assembly scaffolds** | HGA(101, C(21, 2, 50x)) | 321,330 | 249,690 | 302,120 | 249,690 | 102.0 | 1.003 | 207 | 17 | 4 | 14.2 | 3.2 | 57,001 | 0 |
|  |  | HGA(31, M(21, 8, 12x)) | **443,239** | 153,279 | **443,239** | 259,073 | 102.3 | 1.005 | 824 | 31 | 6 | 26.0 | 4.9 | 288,613 | 0 |
|  | **GAGE scaffolds** | GAGE-B | - | - | - | - | - | - | - | - | - | - | - | - | - |
| **MaSuRCA** | **Basic flow** | B(91, 100x) | 33,065 | 33,065 | 33,065 | 33,065 | 101.1 | 1.020 | 246 | 5 | 8 | 5.4 | 3.0 | 453 | 3,458 |
|  | **HGA Preprocessing** | AP(101, 2, 50x) | 49,033 | 49,033 | 47,227 | 47,227 | 100.1 | 1.007 | 179 | 6 | 6 | 4.1 | 2.8 | 719 | 3,439 |
|  |  | C(101, 2, 50x) | 152,046 | 152,046 | 152,046 | 144,720 | 100.0 | 1.000 | 108 | 5 | 5 | 4.2 | 2.9 | 31 | 3,466 |
|  | **HGA re-assembly Contigs** | HGA(81, C(51, 1, 100x)) | 307,457 | 246,453 | 307,457 | 246,453 | 101.8 | 1.002 | 407 | 14 | 3 | 10.4 | 3.5 | 120,450 | 3,541 |
|  |  | HGA(101, M(21, 1, 100x)) | 351,283 | 246,455 | 351,283 | 246,455 | 101.9 | 1.001 | 210 | 8 | 6 | 6.4 | 3.3 | 56,943 | 3,568 |
|  | **GAGE Contigs** | GAGE-B(99) | 76,131 | 76,131 | 76,131 | 76,131 | 101.6 | 1.024 | **173** | 19 | 3 | 6.2 | 3.0 | **811** | 3,538 |
|  | **HGA re-assembly scaffolds** | HGA(81, C(51, 1, 100x)) | 307,457 | 246,453 | 307,457 | 246,453 | 101.8 | 1.002 | 406 | 14 | 4 | 10.2 | 3.3 | 120,450 | 0 |
|  |  | HGA(101, M(21, 1, 100x)) | 355,721 | **355,721** | 351,283 | 246,455 | 101.9 | 1.001 | 209 | 8 | 6 | 6.5 | 3.3 | 56,943 | 0 |
|  | **GAGE scaffolds** | GAGE-B(99) | 76,131 | 76,131 | 76,131 | 76,131 | 101.6 | 1.024 | **173** | 19 | 3 | 6.2 | 3.0 | 811 | 0 |
| **SGA** | **Basic flow** | B(101, 100x) | 46,219 | 46,219 | 46,611 | 46,611 | 102.1 | 1.032 | 671 | 34 | 4 | 3.4 | 2.8 | 7,709 | 3,490 |
|  | **HGA Preprocessing** | AP(81, 2, 50x) | 23,789 | 23,715 | 23,789 | 23,715 | 101.4 | 1.022 | 740 | 21 | 4 | 3.5 | 2.9 | 7,744 | 3,388 |
|  |  | C(101, 2, 50x) | 85,247 | 85,247 | 82,971 | 82,971 | 100.6 | 1.001 | 216 | 4 | 3 | 4.1 | 2.8 | 5,385 | 3,453 |
|  | **HGA re-assembly Contigs** | HGA(91, C(41, 2, 50x)) | 240,631 | 120,569 | 240,631 | 120,569 | 101.9 | 1.002 | 324 | 16 | 6 | 10.4 | 3.2 | 88,422 | 3,549 |
|  |  | HGA(21, M(91, 8, 12x)) | 247,412 | 175,508 | 247,412 | 175,508 | 101.6 | 1.001 | 900 | 15 | 7 | 42.5 | 5.5 | 309,834 | 3,551 |
|  | **GAGE Contigs** | GAGE-B(65) | 23,501 | 23,501 | 27,303 | 27,303 | 102.1 | 1.090 | 1,726 | 77 | 3 | 4.1 | 2.8 | 9,631 | 3,447 |
|  | **HGA re-assembly scaffolds** | HGA(91, C(41, 2, 50x)) | 145,294 | 120,569 | 240,631 | 120,569 | 101.9 | 1.003 | 323 | 17 | 6 | 10.4 | 3.4 | 88,422 | 0 |
|  |  | HGA(21, M(91, 8, 12x)) | 247,412 | 175,508 | 247,412 | 175,508 | 101.6 | 1.001 | 900 | 15 | 7 | 42.5 | 5.5 | 309,834 | 0 |
|  | **GAGE scaffolds** | GAGE-B(65) | 27,303 | 27,303 | 27,303 | 27,303 | 100.8 | 1.024 | 647 | 5 | 2 | **3.3** | 2.7 | **746** | 0 |
| **SOAPd2** | **Basic flow** | B(51, 100x) | 28,882 | 28,882 | 27,948 | 27,948 | 101.0 | 1.002 | 444 | 2 | 0 | 3.1 | 2.7 | 7,151 | 3,368 |
|  | **HGA Preprocessing** | AP(41, 2, 50x) | 15,426 | 15,426 | 14,567 | 14,567 | 100.2 | 1.002 | 696 | 2 | 0 | 3.5 | 2.6 | 6,821 | 3,222 |
|  |  | C(41, 2, 50x) | 33,703 | 33,703 | 31,050 | 31,050 | 100.3 | 1.001 | 390 | 4 | 0 | 4.0 | 2.6 | 6,624 | 3,373 |
|  | **HGA re-assembly Contigs** | HGA(81, C(21, 8, 12x)) | 246,243 | 246,243 | 246,243 | 246,243 | 101.9 | **1.000** | 404 | 10 | 6 | 7.3 | 2.9 | 122,779 | 3,552 |
|  |  | HGA(91, M(21, 1, 100x)) | 336,603 | 246,263 | 336,603 | 246,263 | 101.8 | 1.001 | 328 | 9 | 6 | 6.3 | 3.1 | 96,185 | 3,558 |
|  | **GAGE Contigs** | GAGE-B(49) | 71,357 | 68,152 | 71,357 | 65,464 | 101.2 | 1.003 | 244 | 16 | 35 | 8.2 | 3.3 | 7,695 | 3,453 |
|  | **HGA re-assembly scaffolds** | HGA(81, C(21, 8, 12x)) | 246,243 | 246,243 | 246,243 | 246,243 | 101.9 | **1.000** | 403 | 10 | 6 | 7.3 | 2.9 | 122,779 | 0 |
|  |  | HGA(91, M(21, 1, 100x)) | 336,603 | 246,263 | 336,603 | 246,263 | 101.8 | 1.001 | 327 | 9 | 7 | 6.4 | 3.1 | 96,185 | 0 |
|  | **GAGE scaffolds** | GAGE-B(49) | 91,942 | 89,759 | 91,942 | 89,759 | 101.3 | 1.004 | 212 | 17 | 70 | 8.5 | 3.4 | 7,684 | 0 |
| **SPAdes** | **Basic flow** | B(61, 100x) | 195,243 | 153,030 | 199,486 | 195,243 | 101.6 | 1.002 | 611 | 10 | 3 | 6.8 | 2.9 | 186,269 | 3,523 |
|  | **HGA Preprocessing** | AP(31, 2, 50x) | 106,265 | 92,042 | 106,265 | 93,426 | 101.3 | 1.006 | 561 | 10 | 6 | 17.9 | 4.0 | 144,717 | 3,499 |
|  |  | C(51, 4, 25x) | 162,478 | 152,274 | 162,478 | 146,048 | 101.1 | 1.001 | 172 | 7 | 5 | 8.0 | 2.8 | 16,182 | 3,480 |
|  | **HGA re-assembly Contigs** | HGA(101, C(31, 1, 100x)) | 355,721 | **355,721** | 355,721 | 246,421 | 101.9 | 1.002 | 219 | 9 | 6 | 7.7 | 2.9 | 58,263 | 3,567 |
|  |  | HGA(101, M(31, 1, 100x)) | 355,646 | 246,515 | 355,646 | **355,646** | 101.9 | 1.001 | 481 | 10 | 6 | 8.0 | 2.9 | 183,577 | 3,567 |
|  | **GAGE Contigs** | GAGE-B(33,55,65,75,85,99) | 246,623 | 246,623 | 262,160 | 262,160 | **102.6** | 1.007 | 1,475 | 5 | 4 | 5.0 | 3.0 | 442,574 | **3,602** |
|  | **HGA re-assembly scaffolds** | HGA(101, C(31, 1, 100x)) | 355,721 | **355,721** | 355,721 | 246,421 | 101.9 | 1.002 | 218 | 9 | 7 | 7.9 | 2.9 | 58,263 | 0 |
|  |  | HGA(101, M(31, 1, 100x)) | 355,646 | 246,515 | 355,646 | **355,646** | 101.9 | 1.001 | 480 | 10 | 7 | 8.1 | 2.9 | 183,577 | 0 |
|  | **GAGE scaffolds** | GAGE-B(33,55,65,75,85,99) | 246,623 | 246,623 | 262,160 | 262,160 | **102.6** | 1.007 | 1,474 | 5 | 4 | 5.0 | 3.0 | 448,197 | 0 |
| **Velvet** | **Basic flow** | B(91, 100x) | 93,019 | 93,019 | 92,024 | 92,024 | 100.2 | 1.002 | 178 | 5 | 4 | 5.3 | 2.9 | 6,285 | 3,477 |
|  | **HGA Preprocessing** | AP(51, 2, 50x) | 44,637 | 44,064 | 43,586 | 42,681 | 99.0 | 1.001 | 258 | 8 | 9 | 8.2 | 3.1 | 6,649 | 3,359 |
|  |  | C(41, 4, 25x) | 110,407 | 110,407 | 97,303 | 97,303 | 99.9 | 1.004 | 190 | 4 | 9 | 9.1 | 3.2 | 6,426 | 3,427 |
|  | **HGA re-assembly Contigs** | HGA(81, C(71, 4, 25x)) | 243,230 | 127,745 | 243,230 | 127,745 | 101.9 | 1.002 | 399 | 13 | 7 | 9.5 | 3.0 | 120,450 | 3,544 |
|  |  | HGA(21, M(81, 8, 12x)) | 253,117 | 246,406 | 253,117 | 246,406 | 101.7 | 1.005 | 899 | 16 | 8 | 40.9 | 5.7 | 310,575 | 3,550 |
|  | **GAGE Contigs** | GAGE-B(97) | 92,036 | 67,096 | 92,036 | 63,574 | 101.7 | 1.003 | 201 | 14 | 2 | 4.1 | 2.8 | 6,703 | 3,501 |
|  | **HGA re-assembly scaffolds** | HGA(81, C(71, 4, 25x)) | 243,230 | 127,745 | 243,230 | 127,745 | 101.9 | 1.002 | 399 | 13 | 7 | 9.5 | 3.0 | 120,450 | 0 |
|  |  | HGA(21, M(81, 8, 12x)) | 253,117 | 246,406 | 253,117 | 246,406 | 101.7 | 1.005 | 899 | 16 | 8 | 40.9 | 5.7 | 310,575 | 0 |
|  | **GAGE scaffolds** | GAGE-B(97) | 109,996 | 67,096 | 109,996 | 63,574 | 101.7 | 1.003 | 181 | 27 | 8 | 4.1 | 2.9 | 6,324 | 0 |

**Supplementary Table 9:** 100bp HiSeq read assemblies of *V. cholera*, with reference genome size of 4,033,464bp and 3,693 genes. For metric and assembly flow descriptions see beginning of section.

| **Assembler** | **Type** | **Flow** | **N50** | **NA50** | **NG50** | **NGA50** | **Genome** | **Duplication** | **#** | **# mis-** | **Local mis-** | **# mismatches** | **# indels** | **Unaligned** | **#** |
| --- | --- | --- | --- | --- | --- | --- | --- | --- | --- | --- | --- | --- | --- | --- | --- |
|  |  |  |  |  |  |  | **fraction** | **ratio** | **contigs** | **assemblies** | **assemblies** | **per 100KB** | **per 100KB** | **length** | **Genes** |
| **ABySS** | **Basic flow** | B(51, 110x) | 198,183 | 198,183 | 198,183 | 198,183 | 98.5 | 1.007 | 98 | 2 | 7 | 6.9 | 5.3 | 0 | 3,532 |
|  | **HGA Preprocessing** | AP(41, 2, 55x) | 102,808 | 102,808 | 102,287 | 102,287 | 98.2 | 1.006 | 129 | 5 | 9 | 9.4 | 5.7 | 0 | 3,499 |
|  |  | C(31, 4, 27x) | 102,959 | 102,959 | 98,781 | 98,502 | 97.5 | 1.003 | 178 | 6 | 26 | 49.8 | 8.4 | 1,015 | 3,433 |
|  | **HGA re-assembly Contigs** | HGA(51, C(41, 4, 27x)) | 344,337 | 198,492 | 344,337 | 198,492 | 98.2 | 1.002 | 119 | 9 | 7 | 30.1 | 6.0 | 3,517 | 3,530 |
|  |  | HGA(41, M(51, 8, 13x)) | 351,785 | 246,372 | 351,785 | 246,372 | 98.6 | 1.004 | 105 | 17 | 11 | 15.6 | 4.3 | 3,715 | 3,565 |
|  | **GAGE Contigs** | GAGE-B(51) | 94,508 | 92,996 | 94,508 | 92,996 | 98.7 | 1.037 | 206 | 5 | 17 | 5.5 | 4.9 | 974 | 3,512 |
|  | **HGA re-assembly scaffolds** | HGA(51, C(41, 4, 27x)) | 344,337 | 246,263 | 344,337 | 246,263 | 98.3 | 1.002 | 117 | 10 | 8 | 30.4 | 6.2 | 3,517 | 0 |
|  |  | HGA(41, M(51, 8, 13x)) | 351,785 | 246,372 | 351,785 | 246,372 | 98.6 | 1.004 | 104 | 17 | 11 | 15.5 | 4.3 | 3,715 | 0 |
|  | **GAGE scaffolds** | GAGE-B(51) | 217,596 | 157,127 | 217,596 | 157,127 | 98.7 | 1.040 | 122 | 15 | 68 | 7.1 | 5.1 | 974 | 0 |
| **CABOG** | **Basic flow** | B(21, 110x) | 10,150 | 10,150 | 9,380 | 9,292 | 92.4 | 1.001 | 527 | 8 | 7 | 9.6 | 4.2 | 52 | 2,966 |
|  | **HGA Preprocessing** | AP(21, 2, 55x) | 8,039 | 8,032 | 7,321 | 7,297 | 91.3 | 1.001 | 663 | 8 | 5 | 7.6 | 3.1 | 263 | 2,811 |
|  |  | C(21, 2, 55x) | 13,610 | 13,395 | 11,851 | 11,597 | 91.5 | 1.000 | 449 | 6 | 5 | 5.4 | 2.9 | 424 | 2,986 |
|  | **HGA re-assembly Contigs** | HGA(41, C(21, 2, 55x)) | 246,451 | 184,389 | 246,451 | 184,389 | 98.4 | 1.001 | 136 | 14 | 8 | 14.6 | 3.6 | 3,715 | 3,540 |
|  |  | HGA(41, M(21, 4, 27x)) | 246,376 | 181,216 | 246,376 | 181,216 | 98.2 | 1.004 | 129 | 15 | 10 | 15.5 | 3.6 | 3,715 | 3,536 |
|  | **GAGE Contigs** | GAGE-B | 61,249 | 57,813 | 57,883 | 52,782 | 96.2 | 1.001 | 127 | 20 | 11 | 17.0 | 6.8 | 0 | 3,374 |
|  | **HGA re-assembly scaffolds** | HGA(41, C(21, 2, 55x)) | 251,410 | 198,497 | 251,410 | 198,497 | 98.4 | 1.006 | 132 | 15 | 9 | 15.4 | 3.6 | 3,715 | 0 |
|  |  | HGA(41, M(21, 4, 27x)) | 256,697 | 199,192 | 256,697 | 198,558 | 98.2 | 1.004 | 126 | 15 | 13 | 15.4 | 3.6 | 3,715 | 0 |
|  | **GAGE scaffolds** | GAGE-B | 67,078 | 63,201 | 67,009 | 62,912 | 96.2 | **1.001** | 108 | 21 | 23 | 17.3 | 7.8 | **0** | 0 |
| **MIRA** | **Basic flow** | B(21, 110x) | 8,219 | 8,213 | 15,206 | 15,206 | 98.8 | 2.025 | 2,576 | 118 | 10 | 20.6 | 4.9 | 8,339 | 3,351 |
|  | **HGA Preprocessing** | AP(21, 2, 55x) | 5,925 | 5,899 | 10,177 | 10,090 | 98.7 | 2.026 | 3,186 | 100 | 8 | 19.9 | 4.9 | 5,810 | 3,161 |
|  |  | C(21, 2, 55x) | 66,538 | 66,538 | 65,248 | 65,248 | 97.6 | 1.001 | 224 | 5 | 5 | 7.1 | 3.0 | 35 | 3,451 |
|  | **HGA re-assembly Contigs** | HGA(41, C(21, 2, 55x)) | 343,940 | 198,410 | 246,455 | 175,807 | 98.3 | 1.001 | 118 | 12 | 6 | 13.5 | 3.5 | 3,715 | 3,539 |
|  |  | HGA(51, M(21, 2, 55x)) | 258,705 | 199,146 | 258,705 | 199,146 | **98.8** | 1.004 | 105 | 30 | 9 | 18.4 | 4.0 | 3,777 | 3,571 |
|  | **GAGE Contigs** | GAGE-B | 89,511 | 87,069 | 92,000 | 89,505 | **98.8** | 1.038 | 733 | 54 | 12 | 11.5 | 3.5 | 15,709 | 3,533 |
|  | **HGA re-assembly scaffolds** | HGA(41, C(21, 2, 55x)) | 343,940 | 198,410 | 246,455 | 175,807 | 98.3 | 1.002 | 116 | 12 | 8 | 13.7 | 3.5 | 3,715 | 0 |
|  |  | HGA(51, M(21, 2, 55x)) | 258,705 | 199,146 | 258,705 | 199,146 | **98.8** | 1.004 | 105 | 30 | 9 | 18.4 | 4.0 | 3,777 | 0 |
|  | **GAGE scaffolds** | GAGE-B | - | - | - | - | - | - | - | - | - | - | - | - | - |
| **MaSuRCA** | **Basic flow** | B(81, 110x) | 24,550 | 24,545 | 24,550 | 24,545 | 97.9 | 1.025 | 345 | 5 | 5 | 23.8 | 3.9 | 79 | 3,400 |
|  | **HGA Preprocessing** | AP(51, 2, 55x) | 28,508 | 28,508 | 27,352 | 27,352 | 96.5 | 1.026 | 283 | 5 | 6 | 14.8 | 3.4 | 462 | 3,384 |
|  |  | C(41, 2, 55x) | 116,510 | 116,488 | 116,488 | 116,488 | 97.1 | 1.000 | 143 | 8 | 5 | 17.2 | 3.5 | 0 | 3,439 |
|  | **HGA re-assembly Contigs** | HGA(81, C(31, 2, 55x)) | 394,532 | 198,888 | **394,532** | 198,888 | 98.6 | **1.000** | 86 | 13 | 8 | 11.4 | 3.2 | 280 | 3,561 |
|  |  | HGA(71, M(21, 2, 55x)) | 353,628 | **353,590** | 351,565 | 246,360 | 98.5 | 1.001 | **84** | 11 | 9 | 7.9 | 3.2 | 615 | 3,560 |
|  | **GAGE Contigs** | GAGE-B(89) | 241,604 | 236,373 | 241,604 | 236,373 | **98.8** | 1.009 | 105 | 8 | 5 | 68.6 | 5.7 | 453 | **3,576** |
|  | **HGA re-assembly scaffolds** | HGA(81, C(31, 2, 55x)) | 394,532 | 198,888 | **394,532** | 198,888 | 98.7 | **1.001** | 85 | 14 | 8 | 11.4 | 3.2 | 280 | 0 |
|  |  | HGA(71, M(21, 2, 55x)) | 353,628 | **353,590** | 351,565 | 246,360 | 98.5 | **1.001** | **83** | 11 | 10 | 8.0 | 3.2 | 615 | 0 |
|  | **GAGE scaffolds** | GAGE-B(89) | 246,505 | 236,373 | 246,505 | 236,373 | **98.8** | 1.009 | 102 | 10 | 6 | 68.6 | 5.7 | 453 | 0 |
| **SGA** | **Basic flow** | B(31, 110x) | 25,145 | 25,145 | 24,586 | 24,586 | 97.3 | 1.002 | 452 | 3 | 0 | 3.1 | 2.6 | 817 | 3,339 |
|  | **HGA Preprocessing** | AP(31, 2, 55x) | 13,809 | 13,628 | 13,161 | 13,017 | 97.0 | 1.002 | 679 | 5 | 0 | 3.0 | 2.6 | 813 | 3,185 |
|  |  | C(21, 1, 110x) | 27,336 | 27,336 | 21,768 | 21,768 | 83.1 | 1.001 | 326 | 2 | 0 | 4.3 | 2.8 | 892 | 2,900 |
|  | **HGA re-assembly Contigs** | HGA(41, C(71, 8, 13x)) | 228,218 | 180,513 | 228,218 | 180,513 | 98.1 | 1.003 | 136 | 10 | 5 | 16.2 | 3.5 | 3,715 | 3,527 |
|  |  | HGA(31, M(71, 4, 27x)) | 191,933 | 175,787 | 175,787 | 175,787 | 98.1 | 1.002 | 129 | 9 | 9 | 20.6 | 4.2 | 4,684 | 3,538 |
|  | **GAGE Contigs** | GAGE-B(65) | 23,703 | 23,703 | 23,429 | 23,429 | 97.8 | 1.003 | 485 | **3** | **0** | **3.2** | **2.6** | 808 | 3,337 |
|  | **HGA re-assembly scaffolds** | HGA(41, C(71, 8, 13x)) | 228,218 | 192,230 | 228,218 | 192,230 | 98.1 | 1.003 | 133 | 10 | 7 | 16.6 | 3.7 | 3,715 | 0 |
|  |  | HGA(31, M(71, 4, 27x)) | 191,933 | 175,787 | 175,787 | 175,787 | 98.2 | 1.002 | 125 | 10 | 11 | 22.0 | 4.3 | 4,019 | 0 |
|  | **GAGE scaffolds** | GAGE-B(65) | 23,808 | 23,808 | 23,429 | 23,429 | 96.8 | 1.002 | 430 | **3** | **0** | **3.0** | **2.6** | 551 | 0 |
| **SOAPd2** | **Basic flow** | B(51, 110x) | 21,504 | 21,504 | 20,717 | 20,717 | 97.8 | 1.002 | 462 | 2 | 0 | 3.3 | 2.6 | 2,024 | 3,300 |
|  | **HGA Preprocessing** | AP(41, 2, 55x) | 17,770 | 17,770 | 17,562 | 17,545 | 97.5 | 1.002 | 541 | 2 | 0 | 3.4 | 2.6 | 1,447 | 3,235 |
|  |  | C(31, 4, 27x) | 33,159 | 33,097 | 32,119 | 32,119 | 97.3 | 1.006 | 359 | 2 | 0 | 4.0 | 2.7 | 1,796 | 3,380 |
|  | **HGA re-assembly Contigs** | HGA(61, C(31, 8, 13x)) | 350,677 | 180,774 | 350,677 | 180,774 | 98.3 | 1.001 | 119 | 7 | 2 | 8.8 | 3.0 | 3,342 | 3,539 |
|  |  | HGA(61, M(31, 4, 27x)) | 343,953 | 180,774 | 180,774 | 152,171 | 98.3 | 1.001 | 122 | 7 | 1 | 7.8 | 3.1 | 3,114 | 3,532 |
|  | **GAGE Contigs** | GAGE-B(51) | 135,118 | 106,454 | 125,939 | 106,454 | 98.1 | 1.003 | 139 | 21 | 38 | 12.5 | 3.7 | 2,076 | 3,491 |
|  | **HGA re-assembly scaffolds** | HGA(61, C(31, 8, 13x)) | 350,677 | 260,601 | 350,677 | 198,506 | 98.3 | **1.001** | 116 | 7 | 4 | 8.8 | 3.0 | 3,342 | 0 |
|  |  | HGA(61, M(31, 4, 27x)) | 343,953 | 198,506 | 302,854 | 198,506 | 98.3 | **1.001** | 120 | 7 | 3 | 7.9 | 3.0 | 3,114 | 0 |
|  | **GAGE scaffolds** | GAGE-B(51) | 200,529 | 181,109 | 181,109 | 181,109 | 98.1 | 1.004 | 114 | 21 | 64 | 12.5 | 3.7 | 2,494 | 0 |
| **SPAdes** | **Basic flow** | B(41, 110x) | 176,065 | 176,065 | 176,065 | 151,041 | 98.1 | 1.002 | 172 | 5 | 5 | 10.4 | 3.3 | 3,715 | 3,511 |
|  | **HGA Preprocessing** | AP(41, 2, 55x) | 103,942 | 97,862 | 98,645 | 96,551 | 98.1 | 1.003 | 187 | 6 | 4 | 11.4 | 3.2 | 1,843 | 3,499 |
|  |  | C(51, 4, 27x) | 245,959 | 245,959 | 245,959 | 245,959 | 97.9 | 1.001 | 120 | 4 | 6 | 7.2 | 3.3 | 808 | 3,505 |
|  | **HGA re-assembly Contigs** | HGA(51, C(31, 8, 13x)) | **572,670** | 199,693 | 265,745 | 199,693 | 98.3 | 1.003 | 119 | 11 | 7 | 12.1 | 3.9 | 3,349 | 3,544 |
|  |  | HGA(71, M(41, 4, 27x)) | 352,971 | 246,125 | 246,127 | 246,125 | 98.4 | 1.002 | 87 | 14 | 7 | 10.4 | 3.5 | 2,576 | 3,554 |
|  | **GAGE Contigs** | GAGE-B(33,55,65,75,85,99) | 83,518 | 83,518 | 77,074 | 77,065 | 98.3 | 1.003 | 205 | 4 | 2 | 5.5 | 2.9 | 4,085 | 3,504 |
|  | **HGA re-assembly scaffolds** | HGA(51, C(31, 8, 13x)) | **572,670** | 246,397 | 265,745 | 246,397 | 98.3 | 1.003 | 117 | 11 | 8 | 12.7 | 4.0 | 3,349 | 0 |
|  |  | HGA(71, M(41, 4, 27x)) | 352,971 | 246,125 | 246,127 | 246,125 | 98.4 | 1.002 | 86 | 14 | 8 | 10.5 | 3.5 | 2,576 | 0 |
|  | **GAGE scaffolds** | GAGE-B(33,55,65,75,85,99) | 98,274 | 94,762 | 98,274 | 94,762 | 98.7 | 1.016 | 120 | 20 | 16 | 8.9 | 5.2 | 3,819 | 0 |
| **Velvet** | **Basic flow** | B(41, 110x) | 46,929 | 46,923 | 46,456 | 45,371 | 96.6 | 1.001 | 238 | 8 | 13 | 13.1 | 3.4 | 954 | 3,385 |
|  | **HGA Preprocessing** | AP(41, 2, 55x) | 34,987 | 34,987 | 33,604 | 33,604 | 96.4 | 1.001 | 313 | 6 | 7 | 10.9 | 3.0 | 956 | 3,331 |
|  |  | C(21, 4, 27x) | 80,024 | 78,122 | 78,122 | 74,326 | 96.4 | 1.000 | 192 | 6 | 5 | 20.6 | 7.4 | 1,266 | 3,395 |
|  | **HGA re-assembly Contigs** | HGA(41, C(31, 8, 13x)) | 311,244 | 215,454 | 311,244 | 200,509 | 98.2 | 1.002 | 125 | 11 | 9 | 16.9 | 3.1 | 3,715 | 3,531 |
|  |  | HGA(51, M(31, 4, 27x)) | 356,011 | 246,499 | 356,011 | **246,499** | 98.3 | 1.002 | 108 | 14 | 8 | 12.0 | 3.2 | 3,267 | 3,541 |
|  | **GAGE Contigs** | GAGE-B(49) | 40,877 | 40,877 | 40,085 | 39,462 | 98.0 | 1.001 | 261 | 5 | 6 | 4.5 | 3.4 | 1,069 | 3,404 |
|  | **HGA re-assembly scaffolds** | HGA(41, C(31, 8, 13x)) | 311,244 | 246,407 | 311,244 | 246,407 | 98.2 | 1.006 | 121 | 12 | 11 | 18.5 | 3.1 | 3,715 | 0 |
|  |  | HGA(51, M(31, 4, 27x)) | 356,011 | 246,499 | 356,011 | **246,499** | 98.3 | 1.002 | 107 | 14 | 9 | 12.1 | 3.2 | 3,267 | 0 |
|  | **GAGE scaffolds** | GAGE-B(49) | 172,545 | 171,505 | 172,545 | 171,505 | 98.0 | 1.006 | 124 | 11 | 129 | 5.3 | 4.4 | 981 | 0 |

**Supplementary Table 10:** Results of assembling simulated error-free reads

| **K** | **Flow** | **NGA50** | **# contigs** | **# misassemblies** | **Local misassemblies** | **# mismatches per 100Kb** | **#**  **indels per 100Kb** | **Unaligned length** |
| --- | --- | --- | --- | --- | --- | --- | --- | --- |
| **21** | B(21, 110x) | 287,266 | 44 | 1 | 27 | 4.03 | 0.63 | 0 |
|  | C(21, 4, 27x) | 299,809 | 48 | 2 | 28 | 3.05 | 0.77 | 0 |
|  | HGA(21, C(21, 8, 13x)) | 339,413 | 40 | 1 | 32 | 2.93 | 0.69 | 0 |
|  | HGA(21, M(81, 4, 27x)) | 972,133 | 22 | 2 | 4 | 3.36 | 0.24 | 0 |
| **31** | B(31, 110x) | 392,422 | 38 | 0 | 17 | 2.08 | 0.41 | 0 |
|  | C(31, 4, 27x) | 383,207 | 44 | 0 | 19 | 1.76 | 0.4 | 0 |
|  | HGA(31, C(81, 8, 13x)) | 392,454 | 38 | 0 | 18 | 1.95 | 0.47 | 0 |
|  | HGA(31, M(81, 4, 27x)) | 972,187 | 22 | 1 | 4 | 3.03 | 0.24 | 0 |
| **41** | B(41, 110x) | 654,250 | 30 | 0 | 11 | 1.14 | 0.29 | 0 |
|  | C(41, 8, 13x) | 417,031 | 36 | 0 | 15 | 0.79 | 0.32 | 0 |
|  | HGA(41, C(91, 8, 13x)) | 654,250 | 30 | 0 | 11 | 1.14 | 0.29 | 0 |
|  | HGA(41, M(81, 4, 27x)) | 972,243 | 18 | 0 | 4 | 1.95 | 0.26 | 0 |
| **51** | B(51, 110x) | 654,246 | 28 | 0 | 8 | 0.39 | 0.18 | 0 |
|  | C(51, 2, 55x) | 654,260 | 32 | 0 | 9 | 0.63 | 0.24 | 0 |
|  | HGA(51, C(21, 4, 27x)) | 654,269 | 33 | 0 | 6 | 2.12 | 0.26 | 0 |
|  | HGA(51, M(81, 4, 27x)) | 972,263 | 18 | 0 | 4 | 1.75 | 0.26 | 0 |
| **61** | B(61, 110x) | 684,726 | 27 | 0 | 7 | 0.12 | 0.18 | 0 |
|  | C(61, 2, 55x) | 684,630 | 30 | 0 | 7 | 0.22 | 0.18 | 0 |
|  | HGA(61, C(91, 8, 13x)) | 684,726 | 27 | 0 | 7 | 0.12 | 0.18 | 0 |
|  | HGA(61, M(81, 4, 27x)) | 972,283 | 17 | 0 | 4 | 1.3 | 0.28 | 0 |
| **71** | B(71, 110x) | 684,901 | 25 | 0 | 2 | 0.06 | 0.06 | 0 |
|  | C(71, 2, 55x) | 684,630 | 30 | 0 | 4 | 0.1 | 0.1 | 0 |
|  | HGA(71, C(91, 8, 13x)) | 684,901 | 25 | 0 | 2 | 0.06 | 0.06 | 0 |
|  | HGA(71, M(81, 4, 27x)) | 972,458 | 17 | 0 | 4 | 1.26 | 0.28 | 0 |
| **81** | B(81, 110x) | 684,921 | 26 | 0 | 2 | 0.06 | 0.06 | 0 |
|  | C(81, 4, 27x) | 520,881 | 32 | 1 | 3 | 2.79 | 0.24 | 0 |
|  | HGA(81, C(91, 8, 13x)) | 684,921 | 26 | 0 | 2 | 0.06 | 0.06 | 0 |
|  | HGA(81, M(81, 4, 27x)) | 972,478 | 19 | 0 | 4 | 1.26 | 0.28 | 0 |
| **91** | B(91, 110x) | 41,858 | 204 | 0 | 2 | 0.02 | 0.06 | 0 |
|  | C(91, 1, 110x) | 74,259 | 209 | 0 | 0 | 0.36 | 0 | 0 |
|  | HGA(91, C(61, 8, 13x)) | 685,023 | 25 | 0 | 2 | 0.12 | 0.06 | 0 |
|  | HGA(91, M(81, 4, 27x)) | 972,580 | 20 | 0 | 4 | 1.26 | 0.26 | 0 |
| **Max** | **B(81, 110x)** | **684,921** | **26** | **0** | **2** | **0.06** | **0.06** | **0** |
|  | **C(71, 2, 55x)** | **684,630** | **30** | **0** | **4** | **0.1** | **0.1** | **0** |
|  | **HGA(91, C(61, 8, 13x))** | **685,023** | **25** | **0** | **2** | **0.12** | **0.06** | **0** |
|  | **HGA(91, M(81, 4, 27x))** | **972,580** | **20** | **0** | **4** | **1.26** | **0.26** | **0** |

Note that, even with error free reads, the repeats still be an assembly problem and will induce ambiguities pattern such as cycles and false branching; Now the flows of basic, combining, and HGA using combined contigs; all of them and even with long kmer sizes couldn’t perform similarly to the HGA method using merged contigs and long kmer size. For HGA using merged contigs; the contigs where of coverage P where P is the number of partitions, this is unlike HGA using combined contigs where the contigs is of coverage 1, furthermore when combining contigs of coverage P to create contigs of coverage 1, we may misassemble or disregard a true contigs. This justify why truly HGA using merged is outperform HGA using combined contigs. Laslty, HGA using merged contigs is unlike to the combining flow where the flow is involving assembling the whole reads, and unlike the basic flow where the flow involve just the whole reads with no contigs..

# Contigs combining experiments

As attempt to resolve this; we run minimus2 using large minimum-overlap value; but we noticed that the output of large overlap value has less genome fraction results; and this is explained because true contigs which overlap truly using low overlap value, will be ignored and not outputted. Moreover even with large overlap the duplication ratio and the misassemblies events didn’t decrease much, because we still have non-contiguous contigs that share large x-mer (if not in form of repeats; still some contigs may have assembled the same large region, that form large repeat). In conclusion, we found minimus2 on combining assembly contigs is not effective and misleading; and decided to switch to whether a string graph assembler or a de Bruijn graph assembler.

We run velvet using x-mer sizes 31, 51, 71, 91; hierarchically (binary tree model) and over all partitions' contigs (merging all contigs together). As well, most assembler output assembly with expected coverage equal to 1; so when combining 2, 4 and 8 partitions we input to velvet the expected coverage to be 2, 4 and 8; respectively; instead of the default value which velvet use, {\it auto}. This actually makes the assembly process informative in advance; and therefore helps in detecting repeats, errors and paths finding. When assembling contigs hierarchically in binary tree model, whether using velvet or minimus2; the results show less genome fraction than when assembling all partitions' contigs together; and this justified because some contigs may combine better with other partition's contigs. So we tend to do the contigs assembly by merging all partitions' contigs and then assemble them all. Moreover, all results of running velvet as combiner using 31, 51, 71, and 91 as x-mer length were varying; but the results with 31 value, mostly were the best; so, in this paper, all contigs assembly processes were computed using velvet with 31 as kmer length.

**Supplementary Table 11:** Sample results of contigs assembly, R. sphaeroides HiSeq, and using velvet. Second columns is the results of basic assembly flow; the following columns is the contigs assembly flow, and each column has a column for the tool used to assemble the partitions contigs, velv31 for velvet using kmer=31; Min20 & 80 for minimus2, with minimum overlap value=20 & 80.

| **Metrics** | **B(41, 210x)** | **C(41, 2, 105x)** | | | **C(41, 4, 52x)** | | | **C(41, 8, 26x)** | | |
| --- | --- | --- | --- | --- | --- | --- | --- | --- | --- | --- |
|  |  | **Velv31** | **Min20** | **Min80** | **Velv31** | **Min20** | **Min80** | **Velv31** | **Min20** | **Min80** |
| **Number of contigs** | 959 | 625 | 336 | 372 | 748 | 426 | 479 | 1127 | 630 | 740 |
| **NGA50** | 8,704 | 14,751 | 33,203 | 28,718 | 11,763 | 28,114 | 22,575 | 7,767 | 18,074 | 14,252 |
| **Genome fraction %** | 97.74 | 97.43 | 98.24 | 98.15 | 97.59 | 98.63 | 98.53 | 97.45 | 98.43 | 98.4 |
| **Duplication ratio** | 1.003 | 1.002 | 1.023 | 1.029 | 1.002 | 1.091 | 1.058 | 1.003 | 1.134 | 1.103 |
| **Global misassemblies** | 15 | 1 | 17 | 14 | 1 | 20 | 16 | 1 | 37 | 38 |
| **Local misassemblies** | 6 | 4 | 15 | 9 | 3 | 16 | 6 | 2 | 25 | 13 |
| **Mismatches per 100kb** | 17 | 10 | 11 | 11 | 7 | 15 | 13 | 5 | 22 | 21 |
| **Indels per 100kb** | 1 | 1 | 1 | 1 | 0 | 1 | 1 | 0 | 1 | 1 |
| **# of unaligned contigs** | 1 | 1 | 2 | 4 | 0 | 1 | 7 | 1 | 1 | 3 |

Table 11, shows sample result of contigs assembly using velvet, with 31 kmer; and using minimus2 with 20, 80 overlap values. Firstly, it's clear that the NGA50 values increase considerably with both tools; genome fraction results kept the same with velvet and increase slightly with minimus2. Although both tools increased the NGA50, but each tools show notable differences in the other metrics; namely, duplication ratio, using velvet, kept in similar range compared to the basic flow; while the duplication ratio for both minimus2's assembly (20 and 40 as overlap values) increased *dramatically*; in addition, errors in the contigs in terms of global misassemblies, local misassemblies, mismatches and indels; increase *considerably* with minimus2 while kept the same or even decreased using velvet.

As results, there are two important notes: firstly, the dramatic increase in the error metrics (global misassemblies, local misassemblies, mismatches, indels and unaligned contigs) as well as the duplication ratio, indicate that minimus2 might may have combined contigs falsely, such as two (or more) contigs that are true (aligned) but not contiguous, and share kmer of length 20 or 80; may connected using minimus2 and form a new single contigs; this actually may increase the NGA50 value but false positively. Secondly, it's important for the reassembly process, where we re-assemble a contigs with the reads; to have these contigs as correct as possible, in order to get better reassembly results. Thus, we ignored minimus2 and used velvet, for the contigs assembly process.

# Testing assemblers for the re-assembly step

For this step we mainly considered SPAdes and velvet, as both are de Bruijn graph based and both take long sequences (contigs) as input data. Because this step is intermediate step not final, we didn’t compare the results based on the NA50 correspondent to the highest N50, as the comparison tables above. For this comparison we compared the re-assembly results produced by using SPAdes or Velvet as re-assembler, directly based on the highest NA50.

The commands used to compute the re-assembly process using SPAdes and Velvet respectively are:

$spade_dir/spades.py -k $kmer --12 Whole_reads.fastq --trusted-contigs merged(or combined).fasta -o ./

$velvet_path/velveth ./ $kmer -fasta –long merged(or combined).fasta -fastq -shortPaired Whole_reads.fastq

$velvet_path/velvetg ./ -exp_cov auto -ins_length $mean -ins_length_sd $std -scaffolding no

Note that during the installation of Velvet, Velvet should be installed with option 'LONGSEQUENCES=1' in the make command, to allow Velvet to accept contigs (long sequences) as input; as well and 'MAXKMERLENGTH=111' as the default max kmer length in Velvet installation is 31.

**Supplementary Table 12:** 100bp HiSeq read of *M. abscessus*, with reference genome size of 5,090,401bp. The test results are reported based on the NA50 of the re-assembly process (using the combined contigs *CC* and the merged contigs *MC*) using SPAdes and Velvet as *re-assembler*. The test was conducted over all combinations of kmers used in the partitioning step *PK* and the kmers used in the re-assembly step *RK;* as well using 2, 4, and 8 partitions *P.*

|  |  | **Rk** | **21** | | **31** | | **41** | | **51** | | **61** | | **71** | | **81** | | **91** | |
| --- | --- | --- | --- | --- | --- | --- | --- | --- | --- | --- | --- | --- | --- | --- | --- | --- | --- | --- |
| **CT** | **P** | **Pk** | **SPAdes** | **Velvet** | **SPAdes** | **Velvet** | **SPAdes** | **Velvet** | **SPAdes** | **Velvet** | **SPAdes** | **Velvet** | **SPAdes** | **Velvet** | **SPAdes** | **Velvet** | **SPAdes** | **Velvet** |
| **CC** | **2** | **21** | 123,099 | 17,254 | 231,671 | 21,494 | 225,668 | 36,943 | 225,656 | 118,393 | 175,766 | 104,682 | 143,820 | 68,831 | 98,264 | 40,037 | 46,765 | 39,240 |
|  |  | **31** | 152,087 | 16,577 | 261,099 | 21,494 | 231,757 | 36,102 | 225,660 | 206,566 | 232,830 | 186,960 | 232,849 | 202,126 | 210,217 | 172,373 | 189,572 | 136,708 |
|  |  | **41** | 147,777 | 16,869 | 247,618 | 21,494 | 248,116 | 36,059 | 247,282 | 179,862 | 247,233 | 172,262 | 232,849 | 225,656 | 232,964 | 182,822 | 225,655 | 136,709 |
|  |  | **51** | 143,186 | 16,531 | 245,697 | 21,356 | 226,397 | 36,035 | 230,579 | 179,861 | 232,697 | 185,665 | 225,656 | 209,612 | 225,655 | 166,001 | 176,037 | 120,931 |
|  |  | **61** | 195,121 | 16,869 | 343,659 | 21,343 | 260,075 | 34,956 | 273,546 | 147,220 | 273,546 | 165,991 | 242,913 | 166,001 | 203,144 | 147,203 | 154,029 | 127,866 |
|  |  | **71** | 173,017 | 16,577 | 343,659 | 22,567 | 260,196 | 42,189 | 273,579 | 125,786 | 247,266 | 148,435 | 225,655 | 166,001 | 203,143 | 147,759 | 190,177 | 133,740 |
|  |  | **81** | 153,561 | 16,577 | 278,346 | 21,494 | 232,877 | 36,782 | 247,408 | 125,786 | 233,930 | 127,657 | 214,862 | 147,159 | 191,111 | 122,627 | 122,611 | 119,763 |
|  |  | **91** | 143,186 | 16,577 | 232,801 | 22,785 | 156,564 | 38,850 | 156,496 | 110,393 | 141,421 | 110,681 | 162,440 | 110,813 | 125,801 | 79,781 | 92,700 | 79,678 |
|  | **4** | **21** | 147,154 | 16,869 | 260,186 | 21,494 | 226,076 | 36,059 | 232,411 | 210,139 | 232,830 | 185,683 | 226,577 | 180,140 | 225,656 | 165,235 | 189,566 | 120,772 |
|  |  | **31** | 148,464 | 16,531 | 313,075 | 21,343 | 278,377 | 36,059 | 278,387 | 206,566 | 278,397 | 185,683 | 243,843 | 181,799 | 225,656 | 166,001 | 221,466 | 127,866 |
|  |  | **41** | 148,464 | 16,577 | 313,188 | 21,494 | 260,196 | 36,102 | 260,398 | 179,861 | 247,107 | 156,652 | 243,843 | 156,662 | 225,656 | 149,673 | 190,184 | 119,750 |
|  |  | **51** | 152,286 | 17,254 | 313,188 | 20,838 | 236,908 | 36,059 | 245,440 | 149,669 | 278,397 | 165,991 | 243,845 | 149,665 | 192,651 | 149,665 | 172,817 | 119,735 |
|  |  | **61** | 195,121 | 16,577 | 343,659 | 21,048 | 236,908 | 34,669 | 247,249 | 147,150 | 246,993 | 127,657 | 242,913 | 125,878 | 147,171 | 113,294 | 122,550 | 110,659 |
|  |  | **71** | 198,820 | 16,577 | 343,659 | 22,785 | 237,206 | 41,270 | 273,582 | 125,786 | 236,798 | 125,796 | 213,246 | 121,436 | 174,038 | 122,889 | 125,899 | 106,790 |
|  |  | **81** | 195,121 | 16,531 | 313,228 | 21,343 | 225,668 | 35,998 | 245,599 | 99,051 | 156,737 | 95,090 | 187,805 | 99,024 | 115,838 | 83,318 | 99,016 | 79,784 |
|  |  | **91** | 147,154 | 16,577 | 230,762 | 21,601 | 189,669 | 34,420 | 156,364 | 83,636 | 121,832 | 65,093 | 104,393 | 60,741 | 65,896 | 50,539 | 53,343 | 45,943 |
|  | **8** | **21** | 146,809 | 17,077 | 247,618 | 23,494 | 225,668 | 42,189 | 233,186 | 149,689 | 233,193 | 149,673 | 225,656 | 165,178 | 175,902 | 142,432 | 142,441 | 118,813 |
|  |  | **31** | 147,154 | 16,577 | 234,296 | 21,265 | 225,668 | 34,420 | 234,296 | 125,786 | 187,685 | 127,657 | 187,069 | 104,830 | 187,179 | 127,466 | 155,508 | 104,759 |
|  |  | **41** | 148,410 | 17,254 | 278,346 | 22,071 | 225,668 | 40,798 | 222,355 | 118,393 | 147,176 | 104,839 | 174,949 | 94,968 | 160,295 | 90,524 | 103,310 | 88,121 |
|  |  | **51** | 147,118 | 16,577 | 278,313 | 22,567 | 189,669 | 37,590 | 156,727 | 79,404 | 121,542 | 57,507 | 83,037 | 39,029 | 53,172 | 28,770 | 29,550 | 29,330 |
|  |  | **61** | 112,100 | 17,419 | 209,890 | 20,480 | 156,717 | 32,873 | 125,787 | 47,961 | 77,036 | 23,326 | 34,205 | 7,516 | 9,645 | 1,578 | 895 | 344 |
|  |  | **71** | 187,108 | 16,577 | 278,346 | 21,265 | 225,668 | 34,420 | 156,364 | 75,439 | 147,708 | 70,132 | 98,786 | 59,101 | 82,886 | 52,946 | 55,722 | 44,038 |
|  |  | **81** | 153,280 | 16,531 | 232,801 | 22,529 | 226,430 | 35,479 | 157,176 | 70,051 | 141,428 | 56,768 | 88,620 | 35,914 | 46,037 | 24,300 | 24,549 | 20,923 |
|  |  | **91** | 185,542 | 17,254 | 232,801 | 22,071 | 189,607 | 33,160 | 139,584 | 62,020 | 104,277 | 31,823 | 45,365 | 12,303 | 17,036 | 4,500 | 3,859 | 2,755 |
| ***Maxes*** | | | ***198,820*** | *17,419* | ***343,659*** | *23,494* | ***278,377*** | *42,189* | ***278,387*** | *210,139* | ***278,397*** | *186,960* | ***243,845*** | *225,656* | ***232,964*** | *182,822* | ***225,655*** | *136,709* |
| **MC** | **2** | **21** | 172,184 | 17,231 | 260,186 | 21,494 | 225,668 | 36,102 | 225,668 | 134,634 | 225,668 | 149,673 | 243,843 | 142,539 | 210,217 | 105,368 | 105,381 | 63,445 |
|  |  | **31** | 246,906 | 19,094 | 312,976 | 22,558 | 313,248 | 42,581 | 313,268 | 206,566 | 313,288 | 185,683 | 313,122 | 202,127 | 313,076 | 161,637 | 278,423 | 119,755 |
|  |  | **41** | 278,303 | 19,805 | 313,035 | 22,529 | 278,569 | 39,656 | 278,387 | 163,653 | 278,397 | 225,668 | 278,386 | 226,392 | 278,609 | 226,392 | 278,423 | 132,568 |
|  |  | **51** | 198,820 | 19,805 | 343,659 | 22,422 | 260,196 | 36,102 | 278,387 | 179,861 | 278,589 | 209,821 | 278,386 | 207,809 | 246,999 | 153,244 | 246,623 | 138,346 |
|  |  | **61** | 198,820 | 19,997 | 343,659 | 22,690 | 260,196 | 36,782 | 273,549 | 163,645 | 278,589 | 155,454 | 226,646 | 178,193 | 273,632 | 178,192 | 225,656 | 154,844 |
|  |  | **71** | 198,820 | 19,805 | 343,659 | 23,721 | 237,214 | 43,327 | 273,582 | 149,689 | 278,397 | 147,170 | 226,646 | 144,900 | 225,656 | 147,190 | 225,656 | 147,200 |
|  |  | **81** | 195,121 | 18,839 | 343,659 | 22,529 | 226,358 | 37,876 | 225,656 | 109,384 | 214,894 | 99,050 | 186,389 | 75,837 | 125,894 | 75,300 | 108,085 | 68,566 |
|  |  | **91** | 165,917 | 18,624 | 247,300 | 22,422 | 180,381 | 37,966 | 156,496 | 88,122 | 142,531 | 75,610 | 147,186 | 69,570 | 121,447 | 59,158 | 68,708 | 50,516 |
|  | **4** | **21** | 174,815 | 19,843 | 260,186 | 22,402 | 232,877 | 45,610 | 226,004 | 192,488 | 226,004 | 205,057 | 226,557 | 157,055 | 243,805 | 157,055 | 243,805 | 98,350 |
|  |  | **31** | 195,121 | 19,774 | 343,659 | 22,529 | 278,569 | 43,912 | 278,329 | 147,927 | 278,329 | 205,057 | 278,598 | 202,126 | 278,417 | 168,606 | 226,092 | 127,717 |
|  |  | **41** | 198,820 | 19,843 | 343,659 | 22,529 | 260,196 | 38,611 | 260,189 | 146,446 | 278,329 | 149,744 | 278,308 | 157,596 | 226,392 | 149,731 | 225,656 | 147,190 |
|  |  | **51** | 195,121 | 19,843 | 344,789 | 22,422 | 260,196 | 38,717 | 273,541 | 129,391 | 278,397 | 127,657 | 224,930 | 125,878 | 214,875 | 127,697 | 208,879 | 109,628 |
|  |  | **61** | 195,124 | 19,498 | 343,659 | 22,229 | 236,908 | 36,102 | 273,549 | 149,689 | 246,993 | 154,891 | 242,913 | 127,675 | 214,875 | 110,682 | 163,932 | 104,857 |
|  |  | **71** | 195,124 | 18,879 | 313,089 | 23,494 | 232,792 | 41,791 | 247,214 | 149,689 | 214,894 | 94,948 | 186,389 | 94,968 | 173,271 | 83,153 | 147,191 | 79,794 |
|  |  | **81** | 225,667 | 18,879 | 313,850 | 22,597 | 226,216 | 41,270 | 174,008 | 85,051 | 156,737 | 79,444 | 147,186 | 64,778 | 108,077 | 61,087 | 87,246 | 53,956 |
|  |  | **91** | 209,975 | 18,306 | 230,762 | 22,567 | 157,176 | 38,702 | 155,451 | 79,398 | 119,513 | 52,322 | 91,102 | 41,548 | 51,377 | 26,991 | 33,560 | 21,308 |
|  | **8** | **21** | 172,184 | 19,498 | 260,186 | 22,597 | 225,668 | 42,698 | 232,411 | 102,420 | 232,830 | 118,504 | 226,175 | 104,741 | 210,091 | 78,546 | 200,568 | 59,115 |
|  |  | **31** | 210,016 | 18,833 | 278,346 | 21,601 | 226,325 | 35,998 | 225,656 | 100,178 | 185,479 | 110,681 | 185,489 | 94,968 | 175,902 | 80,377 | 163,902 | 70,161 |
|  |  | **41** | 227,190 | 18,879 | 314,356 | 22,529 | 225,668 | 39,522 | 247,282 | 94,928 | 156,737 | 99,459 | 170,861 | 80,006 | 155,502 | 68,088 | 110,488 | 65,610 |
|  |  | **51** | 175,712 | 18,780 | 231,671 | 21,494 | 189,574 | 35,905 | 177,340 | 81,495 | 139,584 | 69,506 | 95,165 | 51,880 | 62,348 | 29,479 | 33,011 | 24,373 |
|  |  | **61** | 112,100 | 17,584 | 209,890 | 20,246 | 156,717 | 32,873 | 125,787 | 47,961 | 77,036 | 23,586 | 34,205 | 7,516 | 9,481 | 1,587 | 904 | 346 |
|  |  | **71** | 225,667 | 18,306 | 247,266 | 22,597 | 185,626 | 38,132 | 156,397 | 75,503 | 147,176 | 70,072 | 85,087 | 49,002 | 64,063 | 38,121 | 40,562 | 30,362 |
|  |  | **81** | 225,667 | 18,390 | 278,346 | 23,494 | 189,669 | 36,500 | 156,455 | 69,940 | 121,075 | 51,697 | 66,427 | 31,574 | 41,513 | 17,766 | 19,738 | 13,572 |
|  |  | **91** | 225,667 | 17,577 | 232,801 | 21,828 | 189,607 | 35,423 | 139,584 | 61,085 | 88,395 | 31,517 | 47,918 | 12,529 | 17,712 | 4,382 | 3,705 | - |
| ***Maxes*** | | | ***278,303*** | *19,997* | ***344,789*** | *23,721* | ***313,248*** | *45,610* | ***313,268*** | *206,566* | ***313,288*** | *225,668* | ***313,122*** | *226,392* | ***313,076*** | *226,392* | ***278,423*** | *154,844* |

**Supplementary Table 13:** 100bp HiSeq read of *V. cholera*, with reference genome size of 4,033,464bp. The test results are reported based on the NA50 of the re-assembly process (using the combined contigs *CC* and the merged contigs *MC*) using SPAdes and Velvet as *re-assembler*. The test was conducted over all combinations of kmers used in the partitioning step *PK* and the kmers used in the re-assembly step *RK;* as well using 2, 4, and 8 partitions *P.*

|  |  | **Rk** | **21** | | **31** | | **41** | | **51** | | **61** | | **71** | | **81** | | **91** | |
| --- | --- | --- | --- | --- | --- | --- | --- | --- | --- | --- | --- | --- | --- | --- | --- | --- | --- | --- |
| **CT** | **P** | **Pk** | **SPAdes** | **Velvet** | **SPAdes** | **Velvet** | **SPAdes** | **Velvet** | **SPAdes** | **Velvet** | **SPAdes** | **Velvet** | **SPAdes** | **Velvet** | **SPAdes** | **Velvet** | **SPAdes** | **Velvet** |
| **CC** | **2** | **21** | 60,417 | 16,994 | 137,240 | 35,171 | 198,468 | 72,349 | 246,268 | 95,621 | 246,417 | 106,179 | 246,360 | 140,210 | 246,381 | 151,581 | 98,744 | 66,247 |
|  |  | **31** | 61,392 | 16,551 | 106,150 | 33,716 | 198,848 | 72,349 | 199,733 | 97,164 | 199,311 | 110,792 | 225,933 | 126,179 | 198,888 | 129,194 | 135,310 | 81,800 |
|  |  | **41** | 60,567 | 16,719 | 107,884 | 33,716 | 198,492 | 61,861 | 187,797 | 91,944 | 187,895 | 102,721 | 187,781 | 135,138 | 246,591 | 152,441 | 140,875 | 116,602 |
|  |  | **51** | 68,319 | 19,742 | 129,123 | 42,104 | 188,115 | 75,457 | 180,764 | 112,883 | 198,512 | 113,741 | 213,452 | 125,294 | 213,395 | 125,302 | 135,085 | 111,932 |
|  |  | **61** | 68,319 | 16,498 | 129,147 | 34,264 | 198,492 | 72,349 | 199,637 | 90,974 | 201,613 | 94,635 | 163,446 | 81,845 | 127,866 | 88,571 | 105,380 | 75,819 |
|  |  | **71** | 68,319 | 19,343 | 127,817 | 40,280 | 246,377 | 75,468 | 246,397 | 86,148 | 201,646 | 81,069 | 200,332 | 67,944 | 114,971 | 89,132 | 92,002 | 75,748 |
|  |  | **81** | 71,631 | 19,725 | 124,943 | 40,218 | 199,187 | 74,732 | 199,637 | 87,800 | 206,201 | 70,048 | 151,486 | 66,733 | 102,129 | 68,163 | 85,213 | 66,563 |
|  |  | **91** | 68,323 | 16,461 | 109,139 | 34,029 | 175,807 | 72,349 | 198,505 | 80,945 | 199,311 | 66,712 | 109,010 | 63,881 | 95,057 | 51,714 | 59,995 | 43,296 |
|  | **4** | **21** | 65,962 | 21,176 | 130,154 | 42,104 | 198,821 | 76,413 | 199,600 | 97,164 | 191,851 | 112,903 | 216,591 | 112,934 | 124,685 | 112,943 | 99,673 | 71,778 |
|  |  | **31** | 68,319 | 17,358 | 114,798 | 34,029 | 162,815 | 77,422 | 180,696 | 90,974 | 167,903 | 91,964 | 151,486 | 98,700 | 126,782 | 96,302 | 98,584 | 94,782 |
|  |  | **41** | 60,512 | 16,815 | 129,147 | 33,716 | 152,314 | 62,973 | 199,757 | 90,974 | 199,323 | 91,964 | 132,786 | 91,984 | 124,685 | 98,700 | 113,860 | 90,399 |
|  |  | **51** | 71,631 | 19,672 | 143,792 | 40,218 | 199,722 | 72,805 | 180,696 | 86,148 | 180,716 | 90,445 | 127,940 | 82,806 | 115,871 | 75,317 | 85,213 | 71,439 |
|  |  | **61** | 60,567 | 19,725 | 136,129 | 39,781 | 224,346 | 63,686 | 199,712 | 80,945 | 199,311 | 70,242 | 136,986 | 71,416 | 96,380 | 59,062 | 69,903 | 58,902 |
|  |  | **71** | 77,183 | 20,405 | 168,338 | 35,768 | 197,503 | 55,498 | 198,858 | 75,614 | 210,179 | 64,833 | 117,445 | 66,733 | 102,635 | 53,630 | 58,193 | 42,794 |
|  |  | **81** | 72,656 | 16,498 | 168,338 | 32,712 | 192,987 | 59,585 | 192,953 | 63,214 | 180,716 | 63,224 | 117,445 | 50,913 | 90,439 | 39,077 | 46,220 | 36,659 |
|  |  | **91** | 72,656 | 19,343 | 143,808 | 35,327 | 167,847 | 63,202 | 198,505 | 65,786 | 126,399 | 51,287 | 100,643 | 37,769 | 64,783 | 25,329 | 29,641 | 20,597 |
|  | **8** | **21** | 71,631 | 20,405 | 136,492 | 38,041 | 199,590 | 70,443 | 343,935 | 81,818 | 180,716 | 71,396 | 153,810 | 74,893 | 81,084 | 67,036 | 62,655 | 46,009 |
|  |  | **31** | 65,501 | 18,120 | 102,639 | 38,030 | 199,554 | 55,953 | 208,395 | 65,971 | 126,764 | 61,180 | 117,439 | 62,568 | 78,018 | 54,554 | 55,905 | 47,572 |
|  |  | **41** | 70,631 | 16,719 | 129,147 | 32,712 | 180,988 | 59,301 | 180,696 | 63,212 | 152,103 | 51,646 | 109,549 | 45,170 | 68,199 | 32,513 | 37,132 | 31,763 |
|  |  | **51** | 90,617 | 16,994 | 143,746 | 32,711 | 198,477 | 58,416 | 152,969 | 48,727 | 108,757 | 32,527 | 94,604 | 20,328 | 31,485 | 9,667 | 9,424 | 9,845 |
|  |  | **61** | 52,111 | 16,994 | 92,125 | 32,985 | 176,065 | 51,596 | 135,971 | 36,198 | 79,284 | 18,398 | 59,574 | 8,771 | 16,717 | 2,683 | 1,692 | 447 |
|  |  | **71** | 83,702 | 16,551 | 137,067 | 32,712 | 197,840 | 52,911 | 202,016 | 56,578 | 190,593 | 45,959 | 108,767 | 33,735 | 62,386 | 23,154 | 22,640 | 17,103 |
|  |  | **81** | 88,178 | 19,698 | 141,523 | 32,968 | 180,676 | 62,745 | 152,161 | 55,599 | 159,284 | 41,120 | 92,721 | 29,496 | 47,084 | 16,503 | 17,482 | 11,999 |
|  |  | **91** | 77,151 | 16,994 | 92,042 | 35,247 | 176,065 | 51,596 | 135,971 | 39,468 | 92,241 | 26,389 | 90,503 | 13,499 | 24,223 | 5,605 | 4,474 | 2,342 |
| ***Maxes*** | | | ***90,617*** | *21,176* | ***168,338*** | *42,104* | ***246,377*** | *77,422* | ***343,935*** | *112,883* | ***246,417*** | *113,741* | ***246,360*** | *140,210* | ***246,591*** | *152,441* | ***140,875*** | *116,602* |
| **MC** | **2** | **21** | 95,555 | 22,719 | 140,075 | 37,179 | 199,163 | 64,314 | 246,465 | 87,298 | 199,201 | 106,179 | 353,590 | 126,179 | 246,243 | 97,421 | 171,655 | 63,641 |
|  |  | **31** | 86,581 | 20,582 | 129,983 | 35,142 | 174,109 | 62,897 | 199,757 | 87,277 | 207,823 | 101,302 | 207,833 | 104,871 | 198,888 | 109,976 | 174,535 | 90,285 |
|  |  | **41** | 95,555 | 22,023 | 135,629 | 36,056 | 156,235 | 61,861 | 199,200 | 91,944 | 199,210 | 101,357 | 201,656 | 129,184 | 152,355 | 129,194 | 174,535 | 129,204 |
|  |  | **51** | 93,352 | 22,023 | 153,000 | 45,895 | 181,141 | 75,468 | 199,757 | 113,692 | 207,823 | 91,964 | 246,125 | 91,984 | 152,127 | 95,008 | 135,211 | 102,299 |
|  |  | **61** | 82,046 | 21,721 | 165,986 | 34,029 | 165,996 | 64,545 | 152,161 | 90,974 | 199,210 | 68,980 | 198,525 | 72,838 | 142,280 | 74,386 | 107,841 | 72,672 |
|  |  | **71** | 93,352 | 22,827 | 151,742 | 42,027 | 181,035 | 72,719 | 198,505 | 90,624 | 152,335 | 66,753 | 108,489 | 64,828 | 95,940 | 59,817 | 68,941 | 51,953 |
|  |  | **81** | 106,146 | 23,920 | 151,742 | 42,121 | 180,794 | 72,349 | 174,185 | 85,054 | 153,030 | 64,828 | 108,767 | 57,659 | 79,566 | 47,610 | 68,181 | 42,410 |
|  |  | **91** | 102,474 | 22,402 | 198,754 | 35,871 | 198,495 | 62,913 | 174,300 | 71,856 | 152,090 | 59,299 | 126,774 | 45,703 | 71,438 | 38,748 | 42,140 | 27,040 |
|  | **4** | **21** | 95,555 | 23,439 | 168,042 | 44,321 | 198,468 | 75,457 | 344,908 | 107,308 | 199,323 | 96,281 | 216,591 | 107,431 | 216,538 | 89,890 | 113,860 | 63,641 |
|  |  | **31** | 88,259 | 24,917 | 137,122 | 33,716 | 152,668 | 62,957 | 207,813 | 89,964 | 172,778 | 79,489 | 152,698 | 81,086 | 132,867 | 79,004 | 124,757 | 79,998 |
|  |  | **41** | 89,822 | 22,481 | 153,275 | 34,029 | 137,109 | 71,603 | 156,160 | 90,854 | 199,323 | 75,926 | 152,100 | 83,609 | 124,685 | 83,628 | 124,695 | 69,130 |
|  |  | **51** | 103,944 | 23,439 | 156,140 | 42,121 | 156,150 | 63,507 | 156,160 | 87,277 | 156,204 | 71,504 | 137,253 | 70,013 | 115,871 | 66,732 | 85,213 | 53,666 |
|  |  | **61** | 98,525 | 23,439 | 156,122 | 40,155 | 201,593 | 59,374 | 156,142 | 84,131 | 152,171 | 66,940 | 108,834 | 66,939 | 92,147 | 51,646 | 64,899 | 48,191 |
|  |  | **71** | 126,698 | 22,023 | 156,122 | 37,232 | 199,283 | 62,897 | 156,142 | 75,630 | 144,061 | 66,945 | 104,108 | 63,984 | 85,213 | 33,670 | 53,813 | 34,854 |
|  |  | **81** | 126,698 | 21,815 | 151,761 | 30,960 | 180,754 | 62,092 | 152,274 | 63,582 | 144,061 | 57,398 | 92,093 | 40,325 | 68,199 | 31,010 | 45,246 | 26,137 |
|  |  | **91** | 128,460 | 22,976 | 129,147 | 35,247 | 198,492 | 60,193 | 166,580 | 58,106 | 98,701 | 43,090 | 92,721 | 30,521 | 58,988 | 18,929 | 25,502 | 12,899 |
|  | **8** | **21** | 106,146 | 21,434 | 130,164 | 33,971 | 167,863 | 52,263 | 199,303 | 62,790 | 166,947 | 49,665 | 126,808 | 47,103 | 90,439 | 33,167 | 68,181 | 31,858 |
|  |  | **31** | 106,605 | 21,815 | 146,550 | 31,624 | 156,147 | 58,698 | 152,274 | 62,758 | 126,764 | 55,690 | 95,213 | 50,068 | 81,245 | 33,670 | 62,686 | 32,691 |
|  |  | **41** | 126,698 | 22,481 | 152,274 | 31,633 | 175,807 | 62,712 | 198,505 | 66,133 | 152,103 | 43,424 | 104,108 | 39,678 | 69,598 | 30,552 | 42,210 | 29,420 |
|  |  | **51** | 95,885 | 21,816 | 177,014 | 32,740 | 165,996 | 52,263 | 152,103 | 51,629 | 135,555 | 32,790 | 92,721 | 25,148 | 41,066 | 13,199 | 12,247 | 9,754 |
|  |  | **61** | 53,982 | 16,994 | 92,143 | 35,247 | 176,065 | 52,263 | 135,971 | 36,847 | 79,284 | 18,733 | 59,574 | 8,829 | 17,098 | 2,727 | 1,708 | 453 |
|  |  | **71** | 198,477 | 22,408 | 197,767 | 31,624 | 207,776 | 51,515 | 151,466 | 59,764 | 116,287 | 51,211 | 91,988 | 30,969 | 58,779 | 20,087 | 21,829 | 12,631 |
|  |  | **81** | 136,067 | 21,812 | 136,129 | 33,101 | 198,497 | 62,094 | 151,051 | 54,856 | 130,126 | 40,158 | 90,533 | 26,857 | 52,350 | 13,939 | 15,262 | 8,313 |
|  |  | **91** | 91,986 | 19,672 | 105,866 | 30,948 | 167,847 | 51,935 | 130,716 | 36,198 | 92,102 | 26,389 | 70,334 | 13,608 | 24,807 | 5,592 | 4,667 | - |
| ***Maxes*** | | | ***198,477*** | *24,917* | ***198,754*** | *45,895* | ***207,776*** | *75,468* | ***344,908*** | *113,692* | ***207,823*** | *106,179* | ***353,590*** | *129,184* | ***246,243*** | *129,194* | ***174,535*** | *129,204* |

# Assembly commands:

The values of $mean and $std are reported in Table 1.

### ABySS

$abyss_dir/abyss-pe k=$kmer l=1 n=5 s=200 name=asm in=file.fastq

### CABOG

echo "unitigger = bog" > config

$cabog_dir/fastqToCA -insertsize $mean $std -libraryname reads -mates file.fastq > ./reads.frg

$cabog_dir/runCA -d ./ -p asm -s config ./reads.frg

### MIRA

echo project = MyFirstAssembly > config

echo job = genome,denovo,accurate >> config

echo readgroup = DataIlluminaPairedLib >> config

echo data =file1.fastq file2.fastq >> config

echo technology = solexa >> config

echo template_size = $mean $std >> config

echo "segment_placement = ---> <---" >> config

echo "parameters= -NW:cmrnl=warn" >> config

$mira_path/mira config

### MaSuRCA

echo PATHS > config

echo JELLYFISH_PATH=$MaSuRCA_path >> config

echo SR_PATH=$MaSuRCA_path >> config

echo CA_PATH=$cabog_path >> config

echo END >> config

echo DATA >> config

echo PE= p1 $mean $std file1.fastq file2.fastq >> config

echo END >> config

echo PARAMETERS >> config

echo GRAPH_KMER_SIZE=$kmer >> config

echo NUM_THREADS=8 >> config

echo JF_SIZE=2000000000 >> config

echo END >> config

perl $MaSuRCA_path/masurca config

./assemble.sh

### SGA

sga preprocess --pe-mode 1 -o reads.pp.fastq file1.fastq file2.fastq

sga index --algorithm=ropebwt -t 8 reads.pp.fastq

sga correct -k $k -t 8 -o reads.ec.fastq reads.pp.fastq

sga index --algorithm=ropebwt -t 8 reads.ec.fastq

sga filter -t 8 reads.ec.fastq

sga overlap -m $kmer -t 8 reads.ec.filter.pass.fa

sga assemble -o primary reads.ec.filter.pass.asqg.gz

### SOAPd2

echo [LIB] > config

echo avg_ins=$mean >> config

echo reverse_seq=0 >> config

echo asm_flags=1 >> config

echo rank=1 >> config

echo q1=file1.fastq >> config

echo q2=file2.fastq >> config

$soapdenov2_path/SOAPdenovo-127mer all -K $kmer -F -R -E -s config -o asm -p 4 >> SOAPdenovo.lo

### SPAdes

$spade_dir/spades.py -t 8 -k ${kmer} --12 file.fastq -o ./

### Velvet

During the installation of Velvet, Velvet should be installed with option 'LONGSEQUENCES=1' in the make command, to allow Velvet to accept contigs (long sequences) as input; as well and 'MAXKMERLENGTH=111' as the default max kmer length in Velvet installation is 31.

Now to run the assembly commands:

$velvet_path/velveth ./ $kmer -fastq -shortPaired file.fastq

$velvet_path/velvetg ./ -exp_cov auto -ins_length $mean -ins_length_sd $std -scaffolding no

# Interleaving the paired reads for the datasets tested in this paper

If a reader will test HGA method using the datasets in this paper, then in order to have the same results as the reported results, please note the following.

- Firstly in our tests we used the interleaved (interlaced) fastq file, rather than the 2 paired fastq files.
- Since HGA method involve a partitioning step, note that the order of the reads in the interleaved fastq file should be in the *same order* as was tested in this paper. So, after downloading a datasets from <http://ccb.jhu.edu/gage_b/> ; the downloaded pair fastq files *must* be interleaved using this script (not any other scripts to ensure the order of the reads is the same as was tested in this paper), <https://gist.github.com/ngcrawford/2232505> . The script runs as follow:

python [interleave_fastq.py](https://gist.github.com/ngcrawford/2232505#file-interleave_fastq-py) file_1.fastq file_2.fastq file.fastq

# Partitioning the reads sets:

We assumed the reads in the datasets are already randomized, so we sequentially selected the reads for each partition.

**Note:** If a reader will test HGA methods using the datasets in this paper, then in order to get the same reported results in this paper, reads datasets (fastq file) should be whether *raw* or *clean* as recommended in table 2 for the assembler that will be used to assemble the partitions.

# Combining contigs command

Firstly we merge all parts’ contigs into one file, e.g. merged_contigs.fa, and then we run the following command:

$velvet_path/velveth ./ 31 -fasta -long merged_contigs.fa

$velvet_path/velvetg ./ -exp_cov $parts -scaffolding no

**Note:** we input the *number of parts* as the -exp_cov value. Also, Velvet should be installed with option 'LONGSEQUENCES=1' in the make command, to allow Velvet to accept contigs (long sequences) as input; as well 'MAXKMERLENGTH=111' as the default max kmer length in Velvet installation is 31.

# Re-assembly command

We add the parameter --trusted-contigs and give it the fasta file of the merged or the combined contigs.

$spade_dir/spades.py -t 4 -k ${kmer} --12 file.fastq --trusted-contigs combined(or merged)_contigs.fa -o ./

**Note:** If a reader will test HGA methods using the datasets in this paper, then in order to get the same reported results in this paper, for the re-assembly step reads datasets (fastq file) should be whether *raw* or *clean* as recommended in table 2 for SPAdes assembler, as the re-assembly process was performed using SPAdes assembler.

# QUAST command:

quast.py -R genome.fasta --min-contig 200 contigs.fa -G genes.gff

# Platform:

We tested the method on Linux platform, AMD Opteron(tm) 2.4GH, 256GB Memory, 64 core. Python v2.7.6.
